# The Existence and Uniqueness of Solutions in the Leaf Photosynthesis‐Transpiration‐Stomatal Conductance Model for C3 Plants

**DOI:** 10.1111/gcb.70974

**Published:** 2026-07-24

**Authors:** Yuji Masutomi, Kazuhiko Kobayashi

**Affiliations:** ^1^ Center for Climate Change Adaptation, National Institute for Environmental Studies Tsukuba Japan; ^2^ Graduate School of Agricultural and Life Science, the University of Tokyo Bunkyoku Japan

**Keywords:** C3 plants, carbon and water fluxes, earth system modeling, leaf gas exchanges, mathematical modeling, photosynthesis, stomatal conductance, transpiration

## Abstract

The leaf photosynthesis–transpiration–stomatal conductance model, which consistently describes leaf photosynthesis, transpiration, and stomatal conductance, has been widely used as a standard for quantifying these processes in terrestrial plants. However, since its proposal more than 30 years ago, the model has faced a fundamental mathematical problem: Does a solution always exist? And even if a solution is obtained, can it be guaranteed to be the correct one among potentially multiple mathematical solutions—that is, the one actually realized in nature? Here, we resolve this problem by mathematically proving that the model always yields a unique solution satisfying biologically and physically meaningful criteria. This result establishes a rigorous mathematical theorem on the existence and uniqueness of solutions in the model, thereby ruling out concerns about the non‐existence of solutions and ensuring that past and future estimates satisfying the criteria are correct. These findings provide a robust theoretical foundation for the model and have far‐reaching implications for a broad range of fields, spanning plant and ecosystem research to climate and Earth system studies.

## Graphical abstract

The leaf photosynthesis–transpiration–stomatal conductance model has been widely used to quantify photosynthesis, transpiration, and stomatal conductance in terrestrial plants. However, since its proposal more than 30 years ago, it has remained unclear whether a solution always exists and, even if one is obtained, whether it is the correct one actually realized in nature. Here, by reducing the model to *Z*(*C*_*i*_) = *Z*_*c*_ and classifying all 10 geometrical patterns, we prove that the model always yields a unique biologically and physically meaningful solution, thereby establishing a theorem on the existence and uniqueness of solutions.

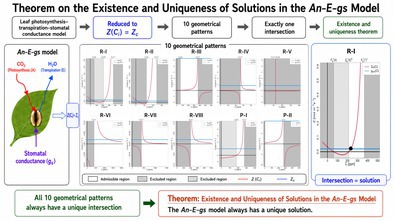

## 1 Introduction

Photosynthesis and transpiration are major components of terrestrial carbon and water cycles. Stomata regulate the exchange of carbon and water between the land surface and the atmosphere, thereby playing a crucial role in global carbon and water cycles. According to Friedlingstein et al. (2022), terrestrial plants absorb 130 PgC year^−1^ of carbon through photosynthesis, which is 12.3 times higher than the 10.9 PgC year^−1^ of carbon emissions caused by human activities, a major contributor to climate change. However, a nearly equivalent amount of carbon is released back into the atmosphere through respiration by plants, soil microbes, and other organisms. As a result, the net carbon uptake by land is only 2.4% (3.1 PgC year^−1^) of the gross carbon absorption. Nevertheless, even this small net uptake mitigates atmospheric CO_2_ increases, slowing climate change and its impacts (Shevliakova et al. 2013; Keenan et al. 2016).

In crops, photosynthesis directly correlates with food production. Even slight changes can impact food security in vulnerable countries, and climate change is expected to significantly affect crop production (Bezner Kerr et al. 2022; Masutomi et al. 2009, 2019; Jägermeyr et al. 2021). Additionally, most terrestrial water transport to the atmosphere occurs through transpiration facilitated by stomata (Jasechko et al. 2013; Schlesinger and Jasechko 2014). With the increasing frequency of water‐related disasters such as floods and droughts driven by climate change (Caretta et al. 2022), accurate estimation of transpiration and stomatal conductance, key factors in the water cycle, is critical for addressing these challenges. Thus, precise representation of photosynthesis, transpiration, and stomatal conductance is fundamental for understanding global carbon and water cycles, climate change, and its impacts.

The leaf photosynthesis ($A_{n}$)–stomatal conductance ($g_{s}$) model, first proposed by Collatz et al. (1991), consists of three coupled submodels: photosynthesis, stomatal conductance, and CO_2_ diffusion. It is widely used as a standard model for quantitatively predicting leaf photosynthesis and stomatal conductance. The $A_{n}$–$g_{s}$ model has been applied not only in ecosystem and vegetation models (Foley et al. 1996; Krinner et al. 2005; Longo et al. 2019; Lawrence et al. 2019) but also in crop, hydrological, land surface, climate, and Earth system models (Masutomi, Ono, Mano, et al. 2016; Masutomi, Ono, Takimoto, et al. 2016; Takata et al. 2003; Zhang et al. 1996; Tatebe et al. 2019; Watanabe et al. 2011; Danabasoglu et al. 2020; Arora 2002). For instance, out of the 16 dynamic global vegatation models employed in the “Global Carbon Project” by researchers worldwide to estimate Earth's carbon balance (Friedlingstein et al. 2022), at least 10 models adopt the $A_{n}$–$g_{s}$ model and its variant forms, with one additional model possibly doing so (Table 1).

**TABLE 1:** Dynamic global vegetation models used in the “Global Carbon Project” (Friedlingstein et al. 2022).

Type	Model			
Collatz et al. (1991)	CABLE‐POP	CLASSIC	CLM5.0	DLEM
	IBIS	JULES‐ES	OCNv2	ORCHIDEEv3
	SDGVM	YIBs	ISAM^a^	
$C_{a}=\lambda C_{i}$ (Haxeltine and Prentice 1996)	JSBACH	LPJ‐GUESS	LPJ	LPX‐Bern
Others	VISIT			

^a^ For ISAM, the use of the Farquhar photosynthesis model and a variant of the stomatal conductance model was confirmed. However, the presence of a CO_2_ diffusion model, and thus whether the components are fully coupled, could not be confirmed.

However, a significant mathematical problem–the existence and uniqueness of solutions, that is, whether a solution exists at all, and if so, whether that solution is the correct one among potentially multiple mathematical solutions–has persisted in the $A_{n}$–$g_{s}$ model for over 30 years since its proposal. Here, by the existence of a solution we mean not merely a mathematical solution, but a biologically and physically appropriate solution, such as those satisfying $g_{s}>0$ and $C_{i}>0$ (internal CO_2_ concentration) (Masutomi 2023). By the correct solution, we mean not just any mathematical solution, but specifically the one actually realized in nature when multiple mathematical solutions exist.

Because the $A_{n}$–$g_{s}$ model is a complex system of equations, the existence of such a solution is not guaranteed. If there are environmental conditions under which no solution exists, then attempts to obtain one through numerical iterative algorithms may fail to converge. In such cases, it becomes impossible to determine whether the failure is due to the absence of a solution or to shortcomings in the numerical algorithm itself.

Moreover, even if a solution exists, it remains uncertain whether it corresponds to the correct solution realized in nature. This difficulty arises because the model reduces to a polynomial equation in terms of the photosynthetic rate or the internal leaf CO_2_ concentration, which mathematically allows for the existence of multiple valid solutions (Baldocchi 1994; Masutomi 2023; Longo et al. 2019). The presence of multiple solutions makes it fundamentally unclear which one is actually realized in nature. As a result, any numerical solution obtained from the model cannot be definitively guaranteed as correct. Even studies using this model–including those contributing to the Global Carbon Project–could potentially be based on incorrect solutions, raising concerns about the validity of their conclusions and implications.

Several studies have addressed the existence and uniqueness problem in the $A_{n}$–$g_{s}$ model. For example, Baldocchi (1994) showed that it reduces to a cubic equation in the photosynthetic rate, yielding three analytical solutions. Baldocchi concluded that one of these three solutions was always appropriate, implying that the existence and uniqueness problem could be resolved in the model.

However, Masutomi (2023) extended the analysis of the model to a broader range of environmental conditions and demonstrated that the appropriate analytical solution is not fixed among the three analytical solutions, but rather varies depending on environmental conditions. Furthermore, the study numerically showed that only one biologically and physically appropriate solution always exists across the investigated range of environmental conditions. This finding provided numerical evidence for the existence and uniqueness of biologically and physically appropriate solutions in the model. Thus, the existence and uniqueness problem is resolved in the $A_{n}$–$g_{s}$ model, at least numerically, but not yet analytically.

In contrast, for an extended form of the $A_{n}$–$g_{s}$ model that couples an H_2_O diffusion submodel to simulate transpiration internally (hereafter referred to as the $A_{n}$–E–$g_{s}$ model), Longo et al. (2019) proposed an algorithm to extract a single solution from the $A_{n}$–E–$g_{s}$ model by analyzing the singularities of functions derived from the model. However, the existence of solutions and the correctness of the solution obtained through their algorithm have not been rigorously proven, and it remains possible that no solution exists or that another mathematically valid solution–different from theirs–is actually realized in nature. Thus, the existence and uniqueness problem remains unresolved for the $A_{n}$–E–$g_{s}$ model.

The objective of this study is to resolve this longstanding problem by analytically demonstrating the existence and uniqueness of biologically and physically appropriate solutions in the $A_{n}$–E–$g_{s}$ model for C_3_ plants. Given the existence and uniqueness of solutions, numerical algorithms can be applied with confidence, and the solution obtained is guaranteed to be the unique correct solution. Here, we focus on the $A_{n}$–E–$g_{s}$ model because it is a more comprehensive model that builds upon the $A_{n}$–$g_{s}$ model and incorporates transpiration as an internal variable. Our proof is inspired by the singularity‐based approach introduced by Longo et al. (2019), and extends it through a rigorous geometric analysis of the functions that comprise the model. By analytically examining the singularities, zeros, and derivatives of these functions, we prove that the model always yields a single biologically and physically appropriate solution–thereby resolving the existence and uniqueness problem.

## 2 Materials and Methods

### 2.1 The $A_{n}$–E–$g_{s}$ Model

The $A_{n}$–E–$g_{s}$ model explored in this study is based on the formulation proposed by Collatz et al. (1991) and explicitly described by Sellers et al. (1992). In this model, the net photosynthesis rate, $A_{n}\left(C_{i}\right)$ [mol(CO_2_) m^−2^ s^−1^], is given by the minimum of the carboxylation rates conrresponding to Rubisco‐limited, RuBP‐limited, and TPU‐limited conditions and the carboxylation rates of each condition follow the framework of Farquhar et al. (1980), as follows:

(1) $$A_{n}\left(C_{i}\right)=\min\left\{W_{c}\left(C_{i}\right),W_{j}\left(C_{i}\right),W_{p}\left(C_{i}\right)\right\}\left\{1-\frac{\Gamma^{*}}{C_{i}}\right\}-R_{L},$$

where

(2) $$\left\{\begin{array}{lll} W_{c}\left(C_{i}\right) & =\frac{V_{\text{cmax}}C_{i}}{C_{i}+K_{c}\left\{1+\frac{O_{2}/P_{a}}{K_{o}}\right\}}, & \left[\text{Rubisco}-\text{limited}\right]\\ W_{j}\left(C_{i}\right) & =\frac{J\;C_{i}}{4C_{i}+8\Gamma^{*}}, & \left[\text{RuBP}-\text{limited}\right]\\ W_{p}\left(C_{i}\right) & =\frac{3T_{p}C_{i}}{C_{i}-\Gamma^{*}}. & \left[\mathrm{TPU}-\text{limited}\right] \end{array}\right.$$

Here, $W_{c}\left(C_{i}\right)$, $W_{j}\left(C_{i}\right)$, and $W_{p}\left(C_{i}\right)$ [mol(CO_2_) m^−2^ s^−1^] represent the Rubisco‐limited, RuBP‐limited, and TPU‐limited carboxylation rates, respectively; $R_{L}$ [mol(CO_2_) m^−2^ s^−1^] is the rate of non‐photorespiratory CO_2_ release in the light (Xu et al. 2021); $V_{\text{cmax}}$ [mol m^−2^ s^−1^] is the maximum rate of carboxylation; J [mol m^−2^ s^−1^] is the electron transport rate; $T_{p}$ [mol m^−2^ s^−1^] is the maximum rate of triose phosphate utilization (TPU); $C_{i}$ [mol mol^−1^] is the internal leaf CO_2_ concentration; $O_{2}$ [Pa] is the atmospheric partial pressure of oxygen; $P_{a}$ [Pa] is the air pressure at the Earth's surface; $\Gamma^{*}$ [mol mol^−1^] is the CO_2_ compensation point where the rate of CO_2_ uptake by carboxylation is balanced by the rate of photorespiratory CO_2_ release; and $K_{c}$ and $K_{o}$ [mol mol^−1^] are the Michaelis–Menten constants for CO_2_ and O_2_, respectively. The mathematical notations used in this study are detailed in SI 0. Equation (3) under the TPU‐limited condition is valid in the range of $C_{i}>\Gamma^{*}$, since $W_{p}\left(C_{i}\right)$ should be positive (Lochocki and McGrath 2025). Thus $C_{i}>\Gamma^{*}$ is assumed under the TPU‐limited condition.

$A_{c}\left(C_{i}\right)$, $A_{j}\left(C_{i}\right)$, and $A_{p}$ [mol(CO_2_) m^−2^ s^−1^] are introduced to represent the gross CO_2_ assimilation rates limited by Rubisco, RuBP, and TPU, respectively, as follows:

(3) $$\left\{\begin{array}{lllll} & A_{c}\left(C_{i}\right) & =W_{c}\left(C_{i}\right)\left\{1-\frac{\Gamma^{*}}{C_{i}}\right\} & =\frac{V_{\text{cmax}}\left\{C_{i}-\Gamma^{*}\right\}}{C_{i}+K_{c}\left\{1+\frac{O_{2}/P_{a}}{K_{o}}\right\}} & \left[\text{Rubisco}-\text{limited}\right]\\ & A_{j}\left(C_{i}\right) & =W_{j}\left(C_{i}\right)\left\{1-\frac{\Gamma^{*}}{C_{i}}\right\} & =\frac{J\left\{C_{i}-\Gamma^{*}\right\}}{4C_{i}+8\Gamma^{*}} & \left[\text{RuBP}-\text{limited}\right]\\ & A_{p} & =W_{p}\left(C_{i}\right)\left\{1-\frac{\Gamma^{*}}{C_{i}}\right\} & =3T_{p} & \left[\mathrm{TPU}-\text{limited}\right] \end{array}\right.$$

A variety of models have been proposed for stomatal conductance during daytime for water vapor (Damour et al. 2010). In this study, we adopt Medlyn's model for daytime conditions ($A_{n}\left(C_{i}\right)>0$) (Medlyn et al. 2011), which is used in recent land surface models (Lawrence et al. 2019; Oliver et al. 2022). For nighttime conditions ($A_{n}\left(C_{i}\right)\leq 0$), $g_{s}$ is assumed to be at its minimum. Therefore, stomatal conductance $g_{s}$ [mol m^−2^ s^−1^] is given by:

(4) $$g_{s}=\left\{\begin{array}{ll} g_{\min}+\lambda_{s}\left\{1+\frac{g_{\mathrm{slp}}}{\sqrt{D}}\right\}\frac{A_{n}\left(C_{i}\right)}{C_{s}} & \text{for}\;A_{n}\left(C_{i}\right)>0\\ g_{\min} & \text{for}\;A_{n}\left(C_{i}\right)\leq 0 \end{array}\right.$$

where $C_{s}$ [mol mol^−1^] is the CO_2_ concentration at the leaf surface; $\lambda_{s}$ [−] is the ratio of the diffusivities of CO_2_ and water vapor through stomata, fixed at 1.6; $g_{\min}$ [mol m^−2^ s^−1^] is the minimum stomatal conductance, and $g_{\mathrm{slp}}$ [Pa^1/2^] represents the sensitivity of $g_{s}$ to variables such as $A_{n}\left(C_{i}\right)$, $C_{s}$, and D. Both $g_{\min}$ and $g_{\mathrm{slp}}$ are plant‐specific parameters. D [Pa] is the vapor pressure deficit at the leaf surface and is given by:

(5) $$D=e_{\mathrm{sat}}-e_{s},$$

where $e_{s}$ [Pa] represents the vapor pressure at the leaf surface, while $e_{\mathrm{sat}}$ [Pa] represents the saturated vapor pressure at the leaf surface and inside the leaf. Note that the equation for $g_{s}$ (Equation 4) is mathematically undefined at D=0 because $g_{s}$ diverges at this point. Therefore, the case of D=0 requires special treatment in the analysis.

The fluxes of CO_2_ and water vapor between the leaf and the environment are governed by standard mass transport relationships (Campbell and Norman 1998). For CO_2_ fluxes, we have:

(6) $$A_{\mathrm{ns}}=\frac{g_{s}}{\lambda_{s}}\left\{C_{s}-C_{i}\right\},$$

(7) $$A_{\mathrm{nb}}=\frac{g_{b}}{\lambda_{b}}\left\{C_{a}-C_{s}\right\},$$

where $A_{\mathrm{ns}}$ and $A_{\mathrm{nb}}$ are the CO_2_ fluxes from the leaf surface to the leaf interior and from the atmosphere to the leaf surface, respectively; $C_{a}$ [mol mol^−1^] is the atmospheric CO_2_ concentration; $g_{b}$ [mol m^−2^ s^−1^] is the conductance of the leaf boundary layer for water vapor; and $\lambda_{b}$ [−] is the ratio of the diffusivities of CO_2_ and water vapor at the leaf surface, fixed at 1.4. Under steady‐state conditions for $C_{i}$ and $C_{s}$, the fluxes $A_{\mathrm{ns}}$ and $A_{\mathrm{nb}}$ become:

(8) $$A_{\mathrm{ns}}=A_{n}\left(C_{i}\right),$$

(9) $$A_{\mathrm{nb}}=A_{n}\left(C_{i}\right).$$

Similarly, the transpiration rate is related to $g_{s}$ and $g_{b}$ as follows:

(10) $$E_{s}=g_{s}\frac{\left\{e_{i}-e_{s}\right\}}{P_{a}},$$

(11) $$E_{b}=g_{b}\frac{\left\{e_{s}-e_{a}\right\}}{P_{a}},$$

where $E_{s}$ and $E_{b}$ [mol (H_2_O) m^−2^ s^−1^] are the transpiration rates from the leaf interior to the surface, and from the surface to the surrounding air, respectively; and $e_{a}$ and $e_{i}$ [Pa] represent the vapor pressures in the air and inside the leaf, respectively. $e_{i}$ is assumed to be saturated and is expressed as $e_{i}=e_{\mathrm{sat}}$. If $e_{i}<e_{a}$ (and thus $e_{s}<e_{a}$), vapor flux occurs from the atmosphere to the leaf surface, causing $e_{s}$ to increase until it reaches saturation, i.e., $e_{s}=e_{\mathrm{sat}}=e_{i}$, resulting in D=0 (Equation 5). Consequently, special treatment is required for this case because $g_{s}$ diverges when D=0. Conversely, when $e_{i}>e_{a}$, vapor flux occurs outward from the leaf interior. Under steady‐state conditions for $e_{s}$, $E_{b}=E_{s}$ is achieved. Thus, we have:

(12) $$\left\{\begin{array}{ll} E_{b}=E_{s} & \text{if}\;e_{a}<e_{i},\\ e_{s}=e_{\mathrm{sat}} & \text{if}\;e_{a}\geq e_{i}. \end{array}\right.$$

The $A_{n}$–E–$g_{s}$ model comprises 12 equations (Equations (1), (2), (3), (4), (5), (6), (7), (8), (9), (10), (11), (12)) and 12 unknowns: $A_{n}$, $W_{c,j,p}$, $A_{c,j,p}$, $A_{\mathrm{ns}}$, $A_{\mathrm{nb}}$, $E_{b}$, $E_{s}$, $C_{i}$, $C_{s}$, $e_{s}$, D, and $g_{s}$. Consequently, these unknowns can be determined internally through the model equations and are referred to as *internal variables*. Other variables, including plant‐specific parameters, are provided externally and are termed *external variables*. Table 2 presents all the internal and external variables in the $A_{n}$–E–$g_{s}$ model, categorizing them based on their possible ranges.

**TABLE 2:** Internal and external variables.

Possible range	Internal variables (unknowns)	External variables
Positive, 0, negative	$A_{n}$, $W_{c,j,p}$, $A_{c,j,p}$, $A_{\mathrm{ns}}$, $A_{\mathrm{nb}}$, $E_{b}$, $E_{s}$	
Positive, 0	$C_{i}$, $C_{s}$, $e_{s}$, D	$O_{2}$, $C_{a}$, $e_{i}$, $e_{a}$, $e_{\mathrm{sat}}$, $P_{a}$, J
Positive	$g_{s}$	$\Gamma^{*}$, $K_{c}$, $K_{o}$, $R_{L}$, $V_{\text{cmax}}$, $g_{b}$, $g_{\mathrm{slp}}$, $g_{\min}$

Flux variables, such as $A_{n}$, $W_{c,j,p}$, $A_{c,j,p}$, $A_{\mathrm{ns}}$, $A_{\mathrm{nb}}$, $E_{s}$, and $E_{b}$, can take positive, negative, or zero values. The remaining variables must be non‐negative, as they represent biological or physical quantities. Furthermore, the non‐negative variables are divided into two categories: those that can be zero and those that cannot. We assume that physical variables can reach zero, whereas biological ones generally cannot, except for J, which can be zero under conditions such as darkness (e.g., at night).

In practice, the transition among $W_{c}$, $W_{j}$, and $W_{p}$ is generally regarded as smooth. To capture this behavior, a transition parameter θ (θ<1, typically close to 1) is often introduced (Collatz et al. 1991), instead of using the minimum function in Equation (1). Regardless of the formulation, determining $A_{n}$ requires the values of $W_{c}$, $W_{j}$, and $W_{p}$ under Rubisco‐limited, RuBP‐limited, and TPU‐limited conditions, respectively. Therefore, it is necessary to establish the existence and uniqueness of solutions under each condition.

As a note, in this model leaf temperature $T_{l}$ is not explicitly treated as an internal variable. A more integrated model, which incorporates the energy balance model (EBM) and treats $T_{l}$ as an internal variable, is discussed in the Section 4.

### 2.2 Net Photosynthesis Rate $A_{n}\left(C_{i}\right)$

As shown in Equation (3), the gross CO_2_ assimilation rates under Rubisco‐limited and RuBP‐limited conditions, $A_{c}$ and $A_{j}$, share the same functional form with respect to $C_{i}$. Therefore, by introducing a single general function, these two limiting cases can be treated simultaneously, which simplifies the subsequent analysis. In this section, we introduce this general function to reformulate the expression for the net photosynthesis rate, $A_{n}\left(C_{i}\right)$.

The expression for the net photosynthesis rate, $A_{n}\left(C_{i}\right)$, in Equation (1) can be reformulated for each limiting condition by combining it with Equation (3), as follows:

(13) $$A_{n}\left(C_{i}\right)=\left\{\begin{array}{lll} \frac{a\left\{C_{i}-\Gamma^{*}\right\}}{bC_{i}+c}-R_{L}\; & =A_{\mathrm{nR}}\left(C_{i}\right)\; & \left[a>0,\text{Rubisco}/\text{RuBP}-\text{limited}\right]\\ -R_{L}\; & =A_{\mathrm{nR}0}\; & \left[a=0,\text{Rubisco}/\text{RuBP}-\text{limited}\right]\\ 3T_{p}-R_{L}\; & =A_{\mathrm{nP}}\; & \left[\mathrm{TPU}-\text{limited}\right] \end{array}\right.$$

where the constants a, b, and c in Equation (13) are defined as:

(14) $$\left\{\begin{array}{ll} a=V_{\text{cmax}},\;b=1,\;c=K_{c}\left\{1+\frac{O_{2}/P_{a}}{K_{o}}\right\} & \left[\text{Rubisco}-\text{limited}\right]\\ a=J,\;b=4,\;c=8\Gamma^{*} & \left[\text{RuBP}-\text{limited}\right] \end{array}\right.$$

Here, we introduce three forms of $A_{n}\left(C_{i}\right)$: $A_{\mathrm{nR}}\left(C_{i}\right)$, $A_{\mathrm{nR}0}$, and $A_{\mathrm{nP}}$. The subscripts “R” and “P” in Equation (13) refer to the two limiting cases: Rubisco/RuBP‐limited and TPU‐limited photosynthesis. “R0” in Equation (13) represents the special case where a=0 under Rubisco/RuBP‐limited condition. This case occurs, for example, at night when J=0 due to the absence of light. Based on Equation (14) and possible range of variables shown in Table 2, we assume the following inequalities:

(15) a≥0,b>0,c>0.

The function $A_{\mathrm{nR}}\left(C_{i}\right)$ depends on $C_{i}$ and can take both positive and negative values. In contrast, $A_{\mathrm{nR}0}$ and $A_{\mathrm{nP}}$ are independent of $C_{i}$. Specifically, $A_{\mathrm{nR}0}$ is always negative (Equation 13), while $A_{\mathrm{nP}}$ is positive under a reasonable range of leaf temperatures (Equation 13). Thus, we assume the following inequalities:

(16) $$A_{\mathrm{nR}0}<0\;\text{and}\;A_{\mathrm{nP}}>0.$$

### 2.3 Two Functions for $g_{s}$: $F\left(C_{i}\right)$ and $G\left(C_{i}\right)$

As described in Section 2.1, the $A_{n}$–E–$g_{s}$ model consists of 12 unknown variables and 12 independent equations, making direct analytical treatment generally intractable. Therefore, it is necessary to introduce a reduced system with fewer variables by eliminating unknowns through substitution. In this section, we introduce a reduced‐order system equivalent to the model described in Section 2.1.

The $A_{n}$–E–$g_{s}$ model can be reduced to two independent equations for $g_{s}$, both expressed as functions of $C_{i}$. The first equation is derived from the CO_2_ flux equations (Equations (6), (7), (8), (9)) and is denoted by $F\left(C_{i}\right)$. The second equation comes from the conductance model (Equation 4) and is represented by $G\left(C_{i}\right)$.

#### 2.3.1 $F\left(C_{i}\right)$: $g_{s}$ From CO_2_ Flux

By eliminating $C_{s}$ from the CO_2_ flux equations (Equations (6), (7), (8), (9)), we can express $g_{s}$ as:

(17) $$g_{s}=F\left(C_{i}\right)=-\frac{\lambda_{s}g_{b}A_{n}\left(C_{i}\right)}{g_{b}\left\{C_{i}-C_{a}\right\}+\lambda_{b}A_{n}\left(C_{i}\right)}$$

Note that $F\left(C_{i}\right)$ has three different forms, corresponding to the three different equations for $A_{n}\left(C_{i}\right)$ (Equation 13), as follows:

(18) $$F\left(C_{i}\right)=\left\{\begin{array}{ll} -\frac{\lambda_{s}g_{b}A_{\mathrm{nR}}\left(C_{i}\right)}{g_{b}\left\{C_{i}-C_{a}\right\}+\lambda_{b}A_{\mathrm{nR}}\left(C_{i}\right)}=F_{R}\left(C_{i}\right) & \left[a>0,\text{Rubisco}/\text{RuBP}-\text{limited}\right]\\ -\frac{\lambda_{s}g_{b}A_{R0}}{g_{b}\left\{C_{i}-C_{a}\right\}+\lambda_{b}A_{\mathrm{nR}0}}=F_{R0}\left(C_{i}\right) & \left[a=0,\text{Rubisco}/\text{RuBP}-\text{limited}\right]\\ -\frac{\lambda_{s}g_{b}A_{P}}{g_{b}\left\{C_{i}-C_{a}\right\}+\lambda_{b}A_{\mathrm{nP}}}=F_{P}\left(C_{i}\right) & \left[\mathrm{TPU}-\text{limited}\right] \end{array}\right.$$

These forms are distinguished by the subscripts “R,” “R0,” and “P,” following the notation for $A_{n}\left(C_{i}\right)$, resulting in $F_{R}\left(C_{i}\right)$, $F_{R0}\left(C_{i}\right)$, and $F_{P}\left(C_{i}\right)$.

#### 2.3.2 $G\left(C_{i}\right)$: $g_{s}$ From the Conductance Model

By eliminating the unknown variables $E_{s}$, $E_{b}$, $e_{s}$, and $g_{s}$ from Equations (5), (10), (11), (12), and (17), the vapor pressure deficit D can be written as:

(19) $$D=\left\{\begin{array}{ll} \left\{e_{i}-e_{a}\right\}\left\{\frac{g_{b}\left\{C_{i}-C_{a}\right\}+\lambda_{b}A_{n}\left(C_{i}\right)}{g_{b}\left\{C_{i}-C_{a}\right\}+\left\{\lambda_{b}-\lambda_{s}\right\}A_{n}\left(C_{i}\right)}\right\} & \left[e_{a}<e_{i}\right]\\ 0 & \left[e_{a}\geq e_{i}\right] \end{array}\right.$$

Substituting Equations (7), (9), and (19) into Equation (4) to eliminate D and $C_{s}$, we obtain:

(20) $$g_{s}=G\left(C_{i}\right)=\left\{\begin{array}{ll} G_{R}\left(C_{i}\right)+g_{\min} & \left[a>0,A_{\mathrm{nR}}\left(C_{i}\right)>0\;\text{and}\;e_{a}<e_{i},\text{Rubisco}/\text{RuBP}-\text{limited}\right]\\ G_{P}\left(C_{i}\right)+g_{\min} & \left[e_{a}<e_{i},\mathrm{TPU}-\text{limited}\right]\\ \infty & \left[A_{n}\left(C_{i}\right)>0\;\text{and}\;e_{a}\geq e_{i}\right]\\ g_{\min} & \left[A_{n}\left(C_{i}\right)\leq 0\right] \end{array}\right.$$

Here, $G_{R}\left(C_{i}\right)$ and $G_{P}\left(C_{i}\right)$ are expressed as:

(21) $$G_{R}\left(C_{i}\right)=\left\{1+{\tilde{g}}_{\mathrm{slp}}\sqrt{\frac{g_{b}\left\{C_{i}-C_{a}\right\}+\left\{\lambda_{b}-\lambda_{s}\right\}A_{\mathrm{nR}}\left(C_{i}\right)}{g_{b}\left\{C_{i}-C_{a}\right\}+\lambda_{b}A_{\mathrm{nR}}\left(C_{i}\right)}}\right\}\left\{-\frac{\lambda_{s}g_{b}A_{\mathrm{nR}}\left(C_{i}\right)}{-g_{b}C_{a}+\lambda_{b}A_{\mathrm{nR}}\left(C_{i}\right)}\right\}$$

(22) $$G_{P}\left(C_{i}\right)=\left\{1+{\tilde{g}}_{\mathrm{slp}}\sqrt{\frac{g_{b}\left\{C_{i}-C_{a}\right\}+\left\{\lambda_{b}-\lambda_{s}\right\}A_{\mathrm{nP}}}{g_{b}\left\{C_{i}-C_{a}\right\}+\lambda_{b}A_{\mathrm{nP}}}}\right\}\left\{-\frac{\lambda_{s}g_{b}A_{\mathrm{nP}}}{-g_{b}C_{a}+\lambda_{b}A_{\mathrm{nP}}}\right\},$$

where ${\tilde{g}}_{\mathrm{slp}}$ is given as:

(23) $${\tilde{g}}_{\mathrm{slp}}=\frac{g_{\mathrm{slp}}}{\sqrt{e_{i}-e_{a}}}$$

### 2.4 Theorem and Criteria

In this paper, we establish the following theorem on the existence and uniqueness of biologically and physically appropriate solutions to the $A_{n}$–E–$g_{s}$ model. The description of the model is provided in Section 2.1.

Theorem 1 *(The Existence and Uniqueness of Biologically and Physically Appropriate Solutions for the* $A_{n}$ *–* E *–* $g_{s}$ *Model) For the* $A_{n}$ *–* E *–* $g_{s}$ *model, there always exists a unique biologically and physically appropriate solution*.

A solution is considered *biologically and physically appropriate* if it satisfies the following criteria:

**Criteria 1**. *(Criteria for Biologically and Physically Appropriate Solutions)* $g_{s}>0$ *(stomatal conductance) and* $C_{i}>0$ *(CO*_*2*_ *concentration inside the leaf)*.

The $A_{n}$–E–$g_{s}$ model may produce multiple solutions for variables such as leaf photosynthesis ($A_{n}$), stomatal conductance ($g_{s}$), and internal CO_2_ concentration ($C_{i}$) (Longo et al. 2019; Masutomi 2023). However, some of these solutions result in negative values for $g_{s}$ and/or $C_{i}$. $C_{i}$ represents the CO_2_ concentration and therefore cannot take negative values from a physical standpoint. As for $g_{s}$, under steady‐state conditions fluxes normally occur from regions of higher concentration to lower concentration, which also precludes negative values for $g_{s}$. Therefore, only solutions satisfying these criteria are classified as *biologically and physically appropriate*. In the following sections, the term “appropriate” exclusively refers to solutions that satisfy the criteria of “biologically and physically appropriate.”

The procedure for applying the theorem and its criteria is as follows. First, a numerical solution to the $A_{n}$–E–$g_{s}$ model should be obtained. It should be noted that the $A_{n}$–E–$g_{s}$ model can be reduced to a fifth‐degree equation (Longo et al. 2019, Masutomi 2023), and therefore its solution must necessarily be derived numerically. Subsequently, the obtained solution should be verified against the criteria ($g_{s}>0$ and $C_{i}>0$). If the solution satisfies these criteria, the theorem guarantees that it is the unique solution and hence the correct one. Conversely, if the solution fails to meet the criteria, another numerical solution satisfying the criteria must exist. In such a case, one should modify the initial conditions or computational settings to obtain this alternative solution. The essential point is that, by virtue of the theorem, the existence of at least one solution satisfying the criteria is mathematically assured.

### 2.5 Steps for Proof

We found that the $A_{n}$–E–$g_{s}$ model, which consists of 12 equations with 12 independent variables (see Section 2.1), can be reduced to two independent functions for stomatal conductance: $F\left(C_{i}\right)$ and $G\left(C_{i}\right)$ (Equations 17 and 20). The function $F\left(C_{i}\right)$ is derived from the CO_2_ flux equations, while $G\left(C_{i}\right)$ originates from the equation describing the stomatal response to environmental conditions. These functions are expressed as:

(24) $$\left\{\begin{array}{l} g_{s}=F\left(C_{i}\right),\\ g_{s}=G\left(C_{i}\right), \end{array}\right.$$

where both $F\left(C_{i}\right)$ and $G\left(C_{i}\right)$ express stomatal conductance, $g_{s}$, as functions of the internal leaf CO_2_ concentration, $C_{i}$. Thus, the value of $C_{i}$ that satisfies all the equations in the $A_{n}$–E–$g_{s}$ model is the one for which $F\left(C_{i}\right)=G\left(C_{i}\right)$ holds.

To facilitate further analysis, we reformulate the equation $F\left(C_{i}\right)=G\left(C_{i}\right)$ as a combination of a function of $C_{i}$ and a constant term:

(25) $$F\left(C_{i}\right)=G\left(C_{i}\right)\Leftrightarrow Z\left(C_{i}\right)=Z_{c},$$

where $Z\left(C_{i}\right)$ represents a function of $C_{i}$, and $Z_{c}$ is a constant. The solution to the $A_{n}$–E–$g_{s}$ model is thus defined as the value of $C_{i}$ that satisfies $Z\left(C_{i}\right)=Z_{c}$.

Using Equations (24) and (25), along with the Criteria for appropriate solutions in the previous section, the appropriate solutions to the $A_{n}$–E–$g_{s}$ model are defined as follows:

Definition 1 (Appropriate Solutions to the $A_{n}$ *–* E *–* $g_{s}$ Model) $C_{i}$ such that $Z\left(C_{i}\right)=Z_{c}$, $F\left(C_{i}\right)>0$, and $C_{i}>0$.

Based on this Definition 1, the proof of the Theorem 1 proceeds through the following steps:

**Step 1:** Identify the possible range of appropriate solutions by selecting the interval of $C_{i}$ where $F\left(C_{i}\right)>0$ and $C_{i}>0$.

**Step 2:** Determine the distinct geometric combinations of $F\left(C_{i}\right)$ and $G\left(C_{i}\right)$ to identify the corresponding distinct geometric combinations of $Z\left(C_{i}\right)$ and $Z_{c}$. Both $F\left(C_{i}\right)$ and $G\left(C_{i}\right)$ exhibit different geometries depending on external variables. Consequently, $Z\left(C_{i}\right)$ and $Z_{c}$ also take on distinct geometries. By identifying the distinct geometric combinations of $F\left(C_{i}\right)$ and $G\left(C_{i}\right)$, the corresponding distinct geometric combinations of $Z\left(C_{i}\right)$ and $Z_{c}$ can be determined.

**Step 3:** Analyze the geometry of $Z\left(C_{i}\right)$ for each combination identified in Step 2, within the possible range of appropriate solutions determined in Step 1.

**Step 4:** Determine the number of solutions for $C_{i}$ that satisfy $Z\left(C_{i}\right)=Z_{c}$, $F\left(C_{i}\right)>0$, and $C_{i}>0$, based on the geometries of $Z\left(C_{i}\right)$ analyzed in Step 3. This involves finding the intersections between $Z\left(C_{i}\right)$ and the line $Z_{c}$ within the possible range of appropriate solutions. If it can be demonstrated that such an intersection always exists and is unique, the Theorem 1 is proven.

## 3 Results

### 3.1 The Possible Range of Appropriate Solutions [Step 1]

The possible range of appropriate solutions, defined as the interval of $C_{i}$ where $F\left(C_{i}\right)>0$ and $C_{i}>0$, is identified in this step. To do so, we analyze the geometries of $F\left(C_{i}\right)$ (Equation 17) and its primary component, $A_{n}\left(C_{i}\right)$ (Equation 13). It should be noted that, in the $A_{n}$–E–$g_{s}$ model, solutions that are biologically or physically inappropriate, such as those with $C_{i}\leq 0$ or $g_{s}\leq 0$, may also exist mathematically.

The function $A_{n}\left(C_{i}\right)$ takes three distinct forms: $A_{\mathrm{nR}}\left(C_{i}\right)$, $A_{\mathrm{nR}0}$, and $A_{\mathrm{nP}}$ (Equation 13). Among these, $A_{\mathrm{nR}0}$ and $A_{\mathrm{nP}}$ are trivial functions, such as negative and positive constants (Equation 16), and therefore do not require further analysis. As a result, this section focuses on $A_{\mathrm{nR}}\left(C_{i}\right)$.

**FIGURE 1:**
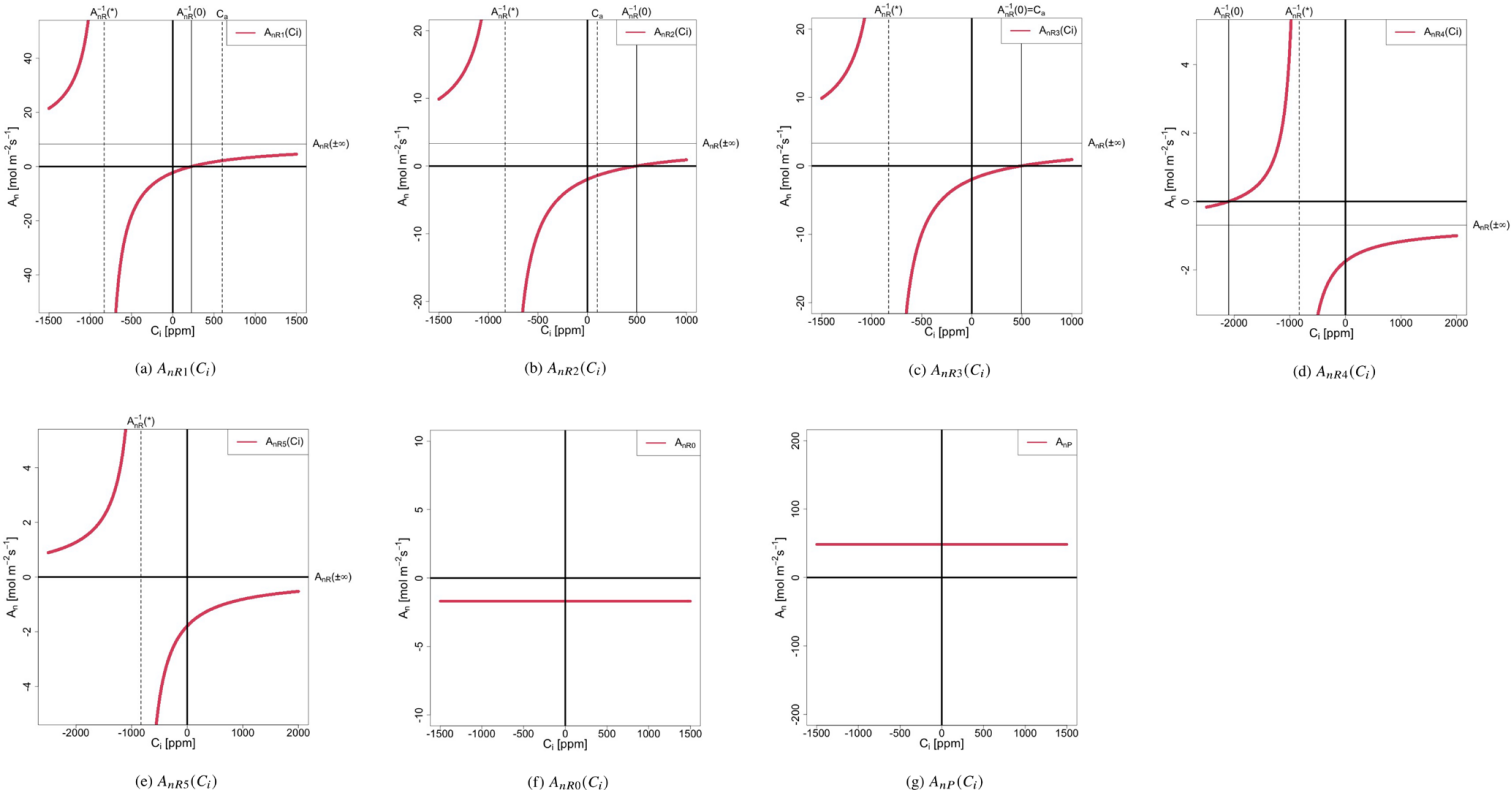
Geometries of $A_{n}\left(C_{i}\right)$. Geometries of $A_{\mathrm{nR}}\left(C_{i}\right)$ are shown in panels (a–e), together with the constant forms $A_{\mathrm{nR}0}$ and $A_{P}$ in panels (f) and (g), respectively.

Our analysis shows that $A_{\mathrm{nR}}\left(C_{i}\right)$ can exhibit five distinct geometries, denoted $A_{\mathrm{nR}1}\left(C_{i}\right)$ through $A_{\mathrm{nR}5}\left(C_{i}\right)$, depending on the external variables. Figure 1a–e illustrate these five geometries. Table 3 summarizes the conditions for these five geometries, along with their corresponding inequality relations among points on the $C_{i}$‐axis that together define the geometric structure of $A_{\mathrm{nR}}\left(C_{i}\right)$: the singularity $A_{\mathrm{nR}}^{-1}\left(*\right)$, the zero $A_{\mathrm{nR}}^{-1}\left(0\right)$ (the CO_2_ compensation point), the air CO_2_ concentration $C_{a}$, and 0. The five conditions are labeled [C1]–[C5]. The inequality relations enumerate all admissible orderings of $A_{\mathrm{nR}}^{-1}\left(*\right)$, $A_{\mathrm{nR}}^{-1}\left(0\right)$, $C_{a}$, and 0. Consequently, the five geometries $A_{\mathrm{nR}1}\left(C_{i}\right)$–$A_{\mathrm{nR}5}\left(C_{i}\right)$ provide an exhaustive classification.

**TABLE 3:** Five patterns of conditions and inequality relations for $A_{\mathrm{nR}}$.

Case	Condition	Inequality relation
[C1]	a>0, $A_{\mathrm{nR}}\left(\pm \infty \right)>0$, $C_{a}-A_{\mathrm{nR}}^{-1}\left(0\right)>0$	$A_{\mathrm{nR}}^{-1}\left(*\right)<0<A_{\mathrm{nR}}^{-1}\left(0\right)<C_{a}$
[C2]	a>0, $A_{\mathrm{nR}}\left(\pm \infty \right)>0$, $C_{a}-A_{\mathrm{nR}}^{-1}\left(0\right)<0$	$A_{\mathrm{nR}}^{-1}\left(*\right)<0\leq C_{a}<A_{\mathrm{nR}}^{-1}\left(0\right)$
[C3]	a>0, $A_{\mathrm{nR}}\left(\pm \infty \right)>0$, $C_{a}-A_{\mathrm{nR}}^{-1}\left(0\right)=0$	$A_{\mathrm{nR}}^{-1}\left(*\right)<0<C_{a}=A_{\mathrm{nR}}^{-1}\left(0\right)$
[C4]	a>0, $A_{\mathrm{nR}}\left(\pm \infty \right)<0$	$A_{\mathrm{nR}}^{-1}\left(0\right)<A_{\mathrm{nR}}^{-1}\left(*\right)<0\leq C_{a}$
[C5]	a>0, $A_{\mathrm{nR}}\left(\pm \infty \right)=0$	$A_{\mathrm{nR}}^{-1}\left(*\right)<0\leq C_{a}$

*Note:* a represents the maximum rate of carboxylation or the electron transport rate under Rubisco/RuBP‐limited conditions (Equation 14), and $A_{\mathrm{nR}}\left(\pm \infty \right)$ denotes the photosynthesis rate as $C_{i}\to \pm \infty$ (Equation S11).

Including the constant forms $A_{\mathrm{nR}0}$ and $A_{P}$, this leads to seven distinct geometries for $A_{n}\left(C_{i}\right)$. Figure 1f,g show the geometries of $A_{\mathrm{nR}0}$ and $A_{P}$.

**FIGURE 2:**
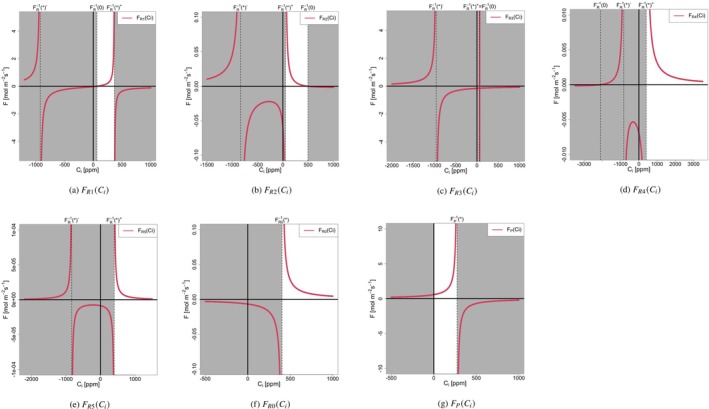
Geometry of $F\left(C_{i}\right)$. Regions outside the possible range of appropriate solutions are shaded in grey. $F_{R}^{-1}\left(0\right)$ represents the zero of $F_{R1}\left(C_{i}\right)$ through $F_{R4}\left(C_{i}\right)$ (Equation S93), while $F_{R}^{-1}{\left(*\right)}^{+}$ and $F_{R}^{-1}{\left(*\right)}^{-}$ represent the singularities of these same functions (Equation S86). Additionally, $F_{R0}^{-1}\left(*\right)$ and $F_{P}^{-1}\left(*\right)$ denote the singularities of $F_{R0}\left(C_{i}\right)$ and $F_{P}\left(C_{i}\right)$, respectively (Equations S120 and S136). Note that $F_{R3}\left(C_{i}\right)$ takes the form 0/0 at $C_{i}=F_{R}^{-1}\left(*\right)=F_{R}^{-1}\left(0\right)$, rendering it indeterminate at this point.

According to the seven distinct geometries of $A_{n}\left(C_{i}\right)$, the function $F\left(C_{i}\right)$ also exhibits seven distinct geometries (Equation 17). These geometries are denoted with the same subscripts as those for $A_{n}\left(C_{i}\right)$: $F_{R1}\left(C_{i}\right)$ through $F_{R5}\left(C_{i}\right)$, $F_{R0}\left(C_{i}\right)$, and $F_{P}\left(C_{i}\right)$. Figure 2 illustrates these geometries, explicitly indicating the zeros and singularities of $F\left(C_{i}\right)$. Note that $F_{R3}\left(C_{i}\right)$ takes the form 0/0 at $C_{i}=F_{R}^{-1}\left(*\right)=F_{R}^{-1}\left(0\right)$, rendering it indeterminate at this point. Table 4 summarizes the conditions defining the seven geometries of $A_{n}\left(C_{i}\right)$ and $F\left(C_{i}\right)$. These conditions include factors such as limiting conditions (Rubisco/RuBP‐limited or TPU‐limited) and the signs of a, $A_{\mathrm{nR}}\left(\pm \infty \right)$, and $C_{a}-A_{\mathrm{nR}}^{-1}\left(0\right)$. Here a represents the maximum rate of carboxylation or the electron transport rate under Rubisco/RuBP‐limited conditions (Equation 14), and $A_{\mathrm{nR}}\left(\pm \infty \right)$ denotes the photosynthesis rate as $C_{i}\to \pm \infty$ (Equation S11).

**TABLE 4:** Conditions and possible range of appropriate solutions for each geometry of $A_{n}\left(C_{i}\right)$ and $F\left(C_{i}\right)$.

$A_{n}\left(C_{i}\right)$	$F\left(C_{i}\right)$	Limitting	a	$A_{\mathrm{nR}}\left(\pm \infty \right)$	$C_{a}-A_{\mathrm{nR}}^{-1}\left(0\right)$	[C]	Range of appropriate solutions	$A_{n}\left(C_{i}\right)$
$A_{\mathrm{nR}1}\left(C_{i}\right)$	$F_{R1}\left(C_{i}\right)$	Rubisco/RuBP	a>0	$A_{\mathrm{nR}}\left(\pm \infty \right)>0$	$C_{a}-A_{\mathrm{nR}}^{-1}\left(0\right)>0$	[C1]	$F_{R}^{-1}\left(0\right)<C_{i}<F_{R}^{-1}{\left(*\right)}^{+}$	$A_{n}\left(C_{i}\right)>0$
$A_{\mathrm{nR}2}\left(C_{i}\right)$	$F_{R2}\left(C_{i}\right)$	Rubisco/RuBP	a>0	$A_{\mathrm{nR}}\left(\pm \infty \right)>0$	$C_{a}-A_{\mathrm{nR}}^{-1}\left(0\right)<0$	[C2]	$F_{R}^{-1}{\left(*\right)}^{+}<C_{i}<F_{R}^{-1}\left(0\right)$	$A_{n}\left(C_{i}\right)<0$
$A_{\mathrm{nR}3}\left(C_{i}\right)$	$F_{R3}\left(C_{i}\right)$	Rubisco/RuBP	a>0	$A_{\mathrm{nR}}\left(\pm \infty \right)>0$	$C_{a}-A_{\mathrm{nR}}^{-1}\left(0\right)=0$	[C3]	$C_{i}=F_{R}^{-1}{\left(*\right)}^{+}=F_{R}^{-1}\left(0\right)$	$A_{n}\left(C_{i}\right)=0$
$A_{\mathrm{nR}4}\left(C_{i}\right)$	$F_{R4}\left(C_{i}\right)$	Rubisco/RuBP	a>0	$A_{\mathrm{nR}}\left(\pm \infty \right)<0$		[C4]	$F_{R}^{-1}{\left(*\right)}^{+}<C_{i}$	$A_{n}\left(C_{i}\right)<0$
$A_{\mathrm{nR}5}\left(C_{i}\right)$	$F_{R5}\left(C_{i}\right)$	Rubisco/RuBP	a>0	$A_{\mathrm{nR}}\left(\pm \infty \right)=0$		[C5]	$F_{R}^{-1}{\left(*\right)}^{+}<C_{i}$	$A_{n}\left(C_{i}\right)<0$
$A_{\mathrm{nR}0}\left(C_{i}\right)$	$F_{R0}\left(C_{i}\right)$	Rubisco/RuBP	a=0				$F_{R0}^{-1}\left(*\right)<C_{i}$	$A_{n}\left(C_{i}\right)<0$
$A_{\mathrm{nP}}\left(C_{i}\right)$	$F_{P}\left(C_{i}\right)$	TPU					$0<C_{i}<F_{P}^{-1}\left(*\right)$	$A_{n}\left(C_{i}\right)>0$

*Note:* $A_{\mathrm{nR}}^{-1}\left(0\right)=F_{R}^{-1}\left(0\right)$ and $H_{R}^{-1}{\left(0\right)}^{+}=F_{R}^{-1}{\left(*\right)}^{+}$ (Equations S93 and S87). The superscripts “+” and “−” attached to $H_{R}^{-1}\left(0\right)$ and $F_{R}^{-1}\left(*\right)$ are used to indicate the positive and negative values of the functions, respectively.

From Figure 2, the possible range of appropriate solutions can be identified by selecting the interval where $F\left(C_{i}\right)>0$ and $C_{i}>0$. Table 4 summarizes the possible ranges of appropriate solutions for each geometry of $F\left(C_{i}\right)$. In Figure 2, regions outside the range are shaded in grey. It is important to note that the possible range of appropriate solutions for $F_{R3}\left(C_{i}\right)$ consists solely of the indeterminate point $C_{i}=F_{R}^{-1}\left(*\right)=F_{R}^{-1}\left(0\right)$. Therefore, special treatment is required for the analysis of $F_{R3}\left(C_{i}\right)$. Additionally, $F\left(C_{i}\right)$ does not have any zeros within the possible range of appropriate solutions, as only the intervals where $F\left(C_{i}\right)>0$ are selected.

Once the possible range of appropriate solutions is determined, the sign of $A_{n}\left(C_{i}\right)$ within that range can be analyzed using the geometries of $A_{n}\left(C_{i}\right)$ (Figure 1a–g). The sign of $A_{n}\left(C_{i}\right)$ is critical because it determines the specific form of $G\left(C_{i}\right)$ in the next step (Equation 20). Table 4 also summarizes the sign of $A_{n}\left(C_{i}\right)$ for each case.

### 3.2 Combination of $Z\left(C_{i}\right)$ and $Z_{c}$ [Step 2]

We explore the distinct geometric combinations of $F\left(C_{i}\right)$ and $G\left(C_{i}\right)$ to identify the corresponding distinct geometric combinations of $Z\left(C_{i}\right)$ and $Z_{c}$.

According to Table 4, only $F_{R1}\left(C_{i}\right)$ and $F_{P}\left(C_{i}\right)$ yield $A_{n}\left(C_{i}\right)>0$ under Rubisco/RuBP‐limited and TPU‐limited conditions, respectively. Based on these results and Equation (20), when $A_{n}\left(C_{i}\right)>0$, there are four possible combinations of $F\left(C_{i}\right)$ and $G\left(C_{i}\right)$: under the Rubisco/RuBP‐limited condition, $F_{R1}\left(C_{i}\right)$ can combine with either $G_{R}\left(C_{i}\right)+g_{\min}$ (when $e_{a}<e_{i}$) or ∞ (when $e_{a}\geq e_{i}$); under the TPU‐limited condition, $F_{P}\left(C_{i}\right)$ can combine with either $G_{P}\left(C_{i}\right)+g_{\min}$ (when $e_{a}<e_{i}$) or ∞ (when $e_{a}\geq e_{i}$). Here, $G_{R}\left(C_{i}\right)$ and $G_{P}\left(C_{i}\right)$ are given by Equations (21) and (22), respectively, while $g_{\min}$ represents the minimum stomatal conductance. The terms $e_{a}$ and $e_{i}$ denote the air and inner leaf vapor pressures, respectively.

For the other five geometries of $F\left(C_{i}\right)$, namely $F_{R2}\left(C_{i}\right)$ through $F_{R5}\left(C_{i}\right)$ and $F_{R0}\left(C_{i}\right)$, we have $A_{n}\left(C_{i}\right)\leq 0$, as indicated in Table 4. Since $G\left(C_{i}\right)=g_{\min}$ when $A_{n}\left(C_{i}\right)\leq 0$ (Equation 20), these five forms of $F\left(C_{i}\right)$ combine only with $g_{\min}$.

Consequently, there are nine possible distinct geometric combinations between $F\left(C_{i}\right)$ and $G\left(C_{i}\right)$, summarized in Table 5. This table also shows the corresponding inequalities between $e_{a}$ and $e_{i}$, and the cases [C1] to [C5] for the conditions on $A_{\mathrm{nR}1}\left(C_{i}\right)$ to $A_{\mathrm{nR}5}\left(C_{i}\right)$ (Table 4).

**TABLE 5:** Distinct geometric combinations between $Z\left(C_{i}\right)$ and $Z_{c}$.

$F\left(C_{i}\right)$	$G\left(C_{i}\right)$	[C]	$e_{a}$ and $e_{i}$	$Z\left(C_{i}\right)$	$Z_{c}$
$F_{R1}\left(C_{i}\right)$	$G_{R}\left(C_{i}\right)+g_{\min}$	[C1]	$e_{a}<e_{i}$	$F_{R1}\left(C_{i}\right)-G_{R}\left(C_{i}\right)\left(=Z_{R}\left(C_{i}\right)\right)$	$g_{\min}$
$F_{R1}\left(C_{i}\right)$	+∞	[C1]	$e_{a}\geq e_{i}$	$F_{R1}\left(C_{i}\right)$	+∞
$F_{R2}\left(C_{i}\right)$	$g_{\min}$	[C2]		$F_{R2}\left(C_{i}\right)$	$g_{\min}$
$F_{R3}\left(C_{i}\right)$	$g_{\min}$	[C3]		$F_{R3}\left(C_{i}\right)$	$g_{\min}$
$F_{R4}\left(C_{i}\right)$	$g_{\min}$	[C4]		$F_{R4}\left(C_{i}\right)$	$g_{\min}$
$F_{R5}\left(C_{i}\right)$	$g_{\min}$	[C5]		$F_{R5}\left(C_{i}\right)$	$g_{\min}$
$F_{R0}\left(C_{i}\right)$	$g_{\min}$			$F_{R0}\left(C_{i}\right)$	$g_{\min}$
$F_{P}\left(C_{i}\right)$	$G_{P}\left(C_{i}\right)+g_{\min}$		$e_{a}<e_{i}$	$F_{P}\left(C_{i}\right)-G_{P}\left(C_{i}\right)\left(=Z_{P}\left(C_{i}\right)\right)$	$g_{\min}$
$F_{P}\left(C_{i}\right)$	+∞		$e_{a}\geq e_{i}$	$F_{P}\left(C_{i}\right)$	+∞

Once the combinations of $F\left(C_{i}\right)$ and $G\left(C_{i}\right)$ are identified, the corresponding distinct geometric combinations of $Z\left(C_{i}\right)$ and $Z_{c}$ can be derived using Equation (25). Table 5 summarizes these combinations of $Z\left(C_{i}\right)$ and $Z_{c}$. Among the nine possible patterns, in two cases $Z\left(C_{i}\right)$ is expressed as the difference between the two functions $F\left(C_{i}\right)$ and $G\left(C_{i}\right)$, whereas in the other cases it is expressed solely by $F\left(C_{i}\right)$. To simplify the notation in the following analysis, the functions expressed by the combination of $F\left(C_{i}\right)$ and $G\left(C_{i}\right)$ are denoted as $Z_{R}\left(C_{i}\right)$ and $Z_{P}\left(C_{i}\right)$, respectively.

(26) $$Z_{R}\left(C_{i}\right)=F_{R1}\left(C_{i}\right)-G_{R}\left(C_{i}\right)$$

(27) $$Z_{P}\left(C_{i}\right)=F_{P}\left(C_{i}\right)-G_{P}\left(C_{i}\right)$$

These two functions, $Z_{R}\left(C_{i}\right)$ and $Z_{P}\left(C_{i}\right)$, are also included in Table 5.

### 3.3 Geometry of $Z\left(C_{i}\right)$ [Step 3]

We examine the geometries of the functions $Z\left(C_{i}\right)$ identified in Step 2, focusing on the possible range of appropriate solutions identified in Step 1. Among the distinct forms of $Z\left(C_{i}\right)$ listed in Table 5, the geometries of the functions $F_{R1}\left(C_{i}\right)$ through $F_{R5}\left(C_{i}\right)$, $F_{R0}\left(C_{i}\right)$, and $F_{P}\left(C_{i}\right)$ have already been identified in Step 1. Therefore, we will focus on $Z_{R}\left(C_{i}\right)$ and $Z_{P}\left(C_{i}\right)$, introduced in Step 2.

**FIGURE 3:**
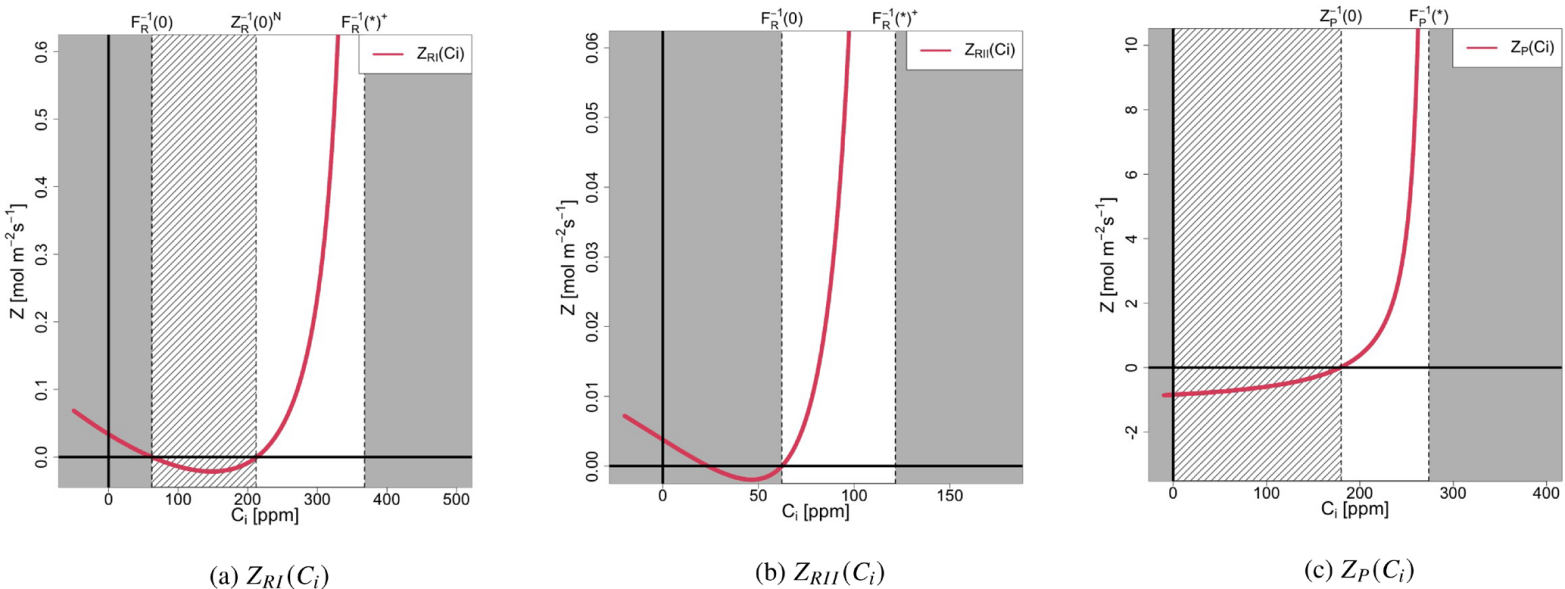
Geometry of $Z_{\mathrm{RI}}\left(C_{i}\right)$, $Z_{\mathrm{RII}}\left(C_{i}\right)$, and $Z_{P}\left(C_{i}\right)$. Regions outside the possible range of appropriate solutions are shaded in grey, while those additionally excluded by the modified range are hatched. $Z_{R}^{-1}{\left(0\right)}^{N}$ and $Z_{P}^{-1}\left(0\right)$ respectively denote zeros of $Z_{\mathrm{RI}}\left(C_{i}\right)$ and $Z_{P}\left(C_{i}\right)$ within the possible range of appropriate solutions.

We find that $Z_{R}\left(C_{i}\right)$ exhibits two distinct geometries within the possible range of appropriate solutions, depending on external variables (the sign of $C_{a}-\left\{A_{\mathrm{nR}}^{-1}\left(0\right)+\frac{A_{\mathrm{nR}}^{-1}\left(0\right)}{{\tilde{g}}_{\mathrm{slp}}}\right\}$ (Equation S412)). One geometry always has a zero within this range, while the other does not. These geometries are denoted as $Z_{\mathrm{RI}}\left(C_{i}\right)$ and $Z_{\mathrm{RII}}\left(C_{i}\right)$, and these two cases are classified as [ZI] and [ZII]. Figures 3a,b display the curves of $Z_{\mathrm{RI}}\left(C_{i}\right)$ and $Z_{\mathrm{RII}}\left(C_{i}\right)$, respectively. The zero of $Z_{\mathrm{RI}}\left(C_{i}\right)$ within the possible range of appropriate solutions is denoted as $Z_{R}^{-1}{\left(0\right)}^{N}$ (Equation S439) and is shown in Figure 3a.

For $Z_{P}\left(C_{i}\right)$, we find that it exhibits only one geometry, which always has a zero within the possible range of appropriate solutions. Figure 3c shows the curve of $Z_{P}\left(C_{i}\right)$. The zero of $Z_{P}\left(C_{i}\right)$ is denoted as $Z_{P}^{-1}\left(0\right)$ (Equation S509) and is also shown in Figure 3c.

Finally, there are ten distinct geometric combinations of $Z\left(C_{i}\right)$ and $Z_{c}$, denoted as [R‐I] to [R‐VIII] for Rubisco/RuBP‐limited conditions and [P‐I] to [P‐II] for TPU‐limited conditions. Tables 6, 7, 8 summarizes these combinations and their associated conditions. Note that there is a zero within the possible range of appropriate solutions only for cases [R‐I] and [P‐I], with no zeros in the other cases. In addition, since the minimal stomatal conductance satisfies $g_{\min}>0$, it follows that $Z_{c}>0$ for all cases (Table 5).

**TABLE 6:** Combinations of $F\left(C_{i}\right)$, $G\left(C_{i}\right)$, $Z\left(C_{i}\right)$, and $Z_{c}$, and their conditions.

	$F\left(C_{i}\right)$	$G\left(C_{i}\right)$	$Z\left(C_{i}\right)$	$Z_{c}$	Limited conditions	a	$A_{\mathrm{nR}}\left(\pm \infty \right)$	$C_{a}-A_{\mathrm{nR}}^{-1}\left(0\right)$	$e_{a}$ and $e_{i}$	$C_{a}-A_{\mathrm{nR}}^{-1}\left(0\right)\left\{1+\frac{1}{{\tilde{g}}_{\mathrm{slp}}}\right\}$	[C]	[Z]
R‐I	$F_{R1}\left(C_{i}\right)$	$G_{R}\left(C_{i}\right)+g_{\min}$	$Z_{\mathrm{RI}}\left(C_{i}\right)$	$g_{\min}$	Rubisco/RuBP	a>0	$A_{\mathrm{nR}}\left(\pm \infty \right)>0$	$C_{a}-A_{\mathrm{nR}}^{-1}\left(0\right)>0$	$e_{a}<e_{i}$	$C_{a}-A_{\mathrm{nR}}^{-1}\left(0\right)\left\{1+\frac{1}{{\tilde{g}}_{\mathrm{slp}}}\right\}>0$	[C1]	[ZI]
R‐II	$F_{R1}\left(C_{i}\right)$	$G_{R}\left(C_{i}\right)+g_{\min}$	$Z_{\mathrm{RII}}\left(C_{i}\right)$	$g_{\min}$	Rubisco/RuBP	a>0	$A_{\mathrm{nR}}\left(\pm \infty \right)>0$	$C_{a}-A_{\mathrm{nR}}^{-1}\left(0\right)>0$	$e_{a}<e_{i}$	$C_{a}-A_{\mathrm{nR}}^{-1}\left(0\right)\left\{1+\frac{1}{{\tilde{g}}_{\mathrm{slp}}}\right\}\leq 0$	[C1]	[ZII]
R‐III	$F_{R1}\left(C_{i}\right)$	+∞	$F_{R1}\left(C_{i}\right)$	+∞	Rubisco/RuBP	a>0	$A_{\mathrm{nR}}\left(\pm \infty \right)>0$	$C_{a}-A_{\mathrm{nR}}^{-1}\left(0\right)>0$	$e_{a}\geq e_{i}$		[C1]	
R‐IV	$F_{R2}\left(C_{i}\right)$	$g_{\min}$	$F_{R2}\left(C_{i}\right)$	$g_{\min}$	Rubisco/RuBP	a>0	$A_{\mathrm{nR}}\left(\pm \infty \right)>0$	$C_{a}-A_{\mathrm{nR}}^{-1}\left(0\right)<0$			[C2]	
R‐V	$F_{R3}\left(C_{i}\right)$	$g_{\min}$	$F_{R3}\left(C_{i}\right)$	$g_{\min}$	Rubisco/RuBP	a>0	$A_{\mathrm{nR}}\left(\pm \infty \right)>0$	$C_{a}-A_{\mathrm{nR}}^{-1}\left(0\right)=0$			[C3]	
R‐VI	$F_{R4}\left(C_{i}\right)$	$g_{\min}$	$F_{R4}\left(C_{i}\right)$	$g_{\min}$	Rubisco/RuBP	a>0	$A_{\mathrm{nR}}\left(\pm \infty \right)<0$				[C4]	
R‐VII	$F_{R5}\left(C_{i}\right)$	$g_{\min}$	$F_{R5}\left(C_{i}\right)$	$g_{\min}$	Rubisco/RuBP	a>0	$A_{\mathrm{nR}}\left(\pm \infty \right)=0$				[C5]	
R‐VIII	$F_{R0}\left(C_{i}\right)$	$g_{\min}$	$F_{R0}\left(C_{i}\right)$	$g_{\min}$	Rubisco/RuBP	a=0						
P‐I	$F_{P}\left(C_{i}\right)$	$G_{P}\left(C_{i}\right)+g_{\min}$	$Z_{P}\left(C_{i}\right)$	$g_{\min}$	TPU				$e_{a}<e_{i}$			
P‐II	$F_{P}\left(C_{i}\right)$	+∞	$F_{P}\left(C_{i}\right)$	+∞	TPU				$e_{a}\geq e_{i}$			

**TABLE 7:** Possible range of appropriate solutions.

	Range	Modified range
R‐I	$F_{R}^{-1}\left(0\right)<C_{i}<F_{R}^{-1}{\left(*\right)}^{+}$	$Z_{R}^{-1}{\left(0\right)}^{N}<C_{i}<F_{R}^{-1}{\left(*\right)}^{+}$
R‐II	$F_{R}^{-1}\left(0\right)<C_{i}<F_{R}^{-1}{\left(*\right)}^{+}$	
R‐III	$F_{R}^{-1}\left(0\right)<C_{i}<F_{R}^{-1}{\left(*\right)}^{+}$	
R‐IV	$F_{R}^{-1}{\left(*\right)}^{+}<C_{i}<F_{R}^{-1}\left(0\right)$	
R‐V	$C_{i}=F_{R}^{-1}{\left(*\right)}^{+}=F_{R}^{-1}\left(0\right)$	
R‐VI	$F_{R}^{-1}{\left(*\right)}^{+}<C_{i}$	
R‐VII	$F_{R}^{-1}{\left(*\right)}^{+}<C_{i}$	
R‐VIII	$F_{R0}^{-1}\left(*\right)<C_{i}$	
P‐I	$0<C_{i}<F_{P}^{-1}\left(*\right)$	$Z_{P}^{-1}\left(0\right)<C_{i}<F_{P}^{-1}\left(*\right)$
P‐II	$0<C_{i}<F_{P}^{-1}\left(*\right)$	

**TABLE 8:** Geometry of $Z\left(C_{i}\right)$.

Combination	$Z\left(C_{i}\right)$ at the left end		$Z\left(C_{i}\right)$ at the right end		$Z^{\prime }\left(C_{i}\right)$ within the range	
R‐I	$Z_{R}\left(Z_{R}^{-1}{\left(0\right)}^{N}\right)=0$	Equation (S437)	$Z_{R}\left(F_{R}^{-1}{\left(*\right)}^{+}\right)=+\infty$	Equation (S435)	$Z_{R}^{\prime }\left(Z_{R}^{-1}{\left(0\right)}^{N}<C_{i}<F_{R}^{-1}{\left(*\right)}^{+}\right)>0$	Equation (S491)
R‐II	$Z_{R}\left(F_{R}^{-1}\left(0\right)\right)=0$	Equation (S437)	$Z_{R}\left(F_{R}^{-1}{\left(*\right)}^{+}\right)=+\infty$	Equation (S435)	$Z_{R}^{\prime }\left(F_{R}^{-1}\left(0\right)<C_{i}<F_{R}^{-1}{\left(*\right)}^{+}\right)>0$	Equation (S491)
R‐III	$F_{R}\left(F_{R}^{-1}\left(0\right)\right)=0$	Equation (S93)	$F_{R}\left(F_{R}^{-1}{\left(*\right)}^{+}\right)=+\infty$	Equation (S87)	$F_{R}^{\prime }\left(F_{R}^{-1}\left(0\right)<C_{i}<F_{R}^{-1}{\left(*\right)}^{+}\right)>0$	Equation (S113)
R‐IV	$F_{R}\left(F_{R}^{-1}{\left(*\right)}^{+}\right)=+\infty$	Equation (S87)	$F_{R}\left(F_{R}^{-1}\left(0\right)\right)=0$	Equation (S93)	$F_{R}^{\prime }\left(F_{R}^{-1}{\left(*\right)}^{+}<C_{i}<F_{R}^{-1}\left(0\right)\right)<0$	Equation (S114)
R‐V	$F_{R}\left(F_{R}^{-1}{\left(*\right)}^{+}\right)=F_{R}\left(F_{R}^{-1}\left(0\right)\right)=\frac{0}{0}$	Equation (S92)				
R‐VI	$F_{R}\left(F_{R}^{-1}{\left(*\right)}^{+}\right)=+\infty$	Equation (S87)	$F_{R}\left(+\infty \right)=0$	Equation (S96)	$F_{R}^{\prime }\left(F_{R}^{-1}{\left(*\right)}^{+}<C_{i}\right)<0$	Equation (S115)
R‐VII	$F_{R}\left(F_{R}^{-1}{\left(*\right)}^{+}\right)=+\infty$	Equation (S87)	$F_{R}\left(+\infty \right)=0$	Equation (S96)	$F_{R}^{\prime }\left(F_{R}^{-1}{\left(*\right)}^{+}<C_{i}\right)<0$	Equation (S117)
R‐VIII	$F_{R0}\left(F_{R0}^{-1}\left(*\right)\right)=+\infty$	Equation (S435)	$F_{R0}\left(+\infty \right)=0$	Equation (S96)	$F_{R0}^{\prime }\left(F_{R0}^{-1}{\left(*\right)}^{+}<C_{i}\right)<0$	Equation (S119)
P‐I	$Z_{P}\left(Z_{P}^{-1}\left(0\right)\right)=0$	Equation (S509)	$Z_{P}\left(F_{P}^{-1}\left(*\right)\right)=+\infty$	Equation (S508)	$Z_{P}^{\prime }\left(Z_{P}^{-1}\left(0\right)<C_{i}<F_{P}^{-1}\left(*\right)\right)>0$	Equation (S513)
P‐II	$F_{P}\left(0\right)>0$	Equation (S138)	$F_{P}\left(F_{P}^{-1}\left(*\right)\right)=+\infty$	Equation (S136)	$F_{P}^{\prime }\left(0<C_{i}<F_{P}^{-1}\left(*\right)\right)>0$	Equation (S135)

If $Z\left(C_{i}\right)$ has zeros within the possible range of appropriate solutions, the range can be refined by excluding the interval of $C_{i}$ where $Z\left(C_{i}\right)<0$. This is because the solutions for $Z\left(C_{i}\right)=Z_{c}$ (Equation 25) must exist within the range of $C_{i}$ where $Z\left(C_{i}\right)>0$, given that $Z_{c}>0$. Tables 6, 7, 8 presents the modified range of possible solutions for cases [R‐I] and [P‐I].

To identify the geometry of $Z\left(C_{i}\right)$ within the possible range of appropriate solutions, we calculated the values of $Z\left(C_{i}\right)$ at the endpoints of these ranges (Tables 6, 7, 8) and the derivatives of $Z\left(C_{i}\right)$ within the ranges. The results of these calculations are summarized in Tables 6, 7, 8. Note that for the cases [R‐I] and [P‐I], the modified possible range of appropriate solutions was used (Tables 6, 7, 8). Additionally, for the case [R‐V], these calculations could not be performed as the range of appropriate solutions consists only of the single indeterminate point $C_{i}=F_{R}^{-1}\left(*\right)=F_{R}^{-1}\left(0\right)$.

We find that the derivatives of $Z\left(C_{i}\right)$ are non‐zero within the possible range of appropriate solutions, except for the case of [R‐V], which indicates that $Z\left(C_{i}\right)$ is either monotonically increasing or decreasing within the range. Furthermore, for all cases except [R‐V] and [P‐II], one end of the possible range of appropriate solutions is a zero of $Z\left(C_{i}\right)$, while the other end is a singularity.

### 3.4 Number of Intersections Between $Z\left(C_{i}\right)$ and $Z_{c}$ [Step 4]

**FIGURE 4:**
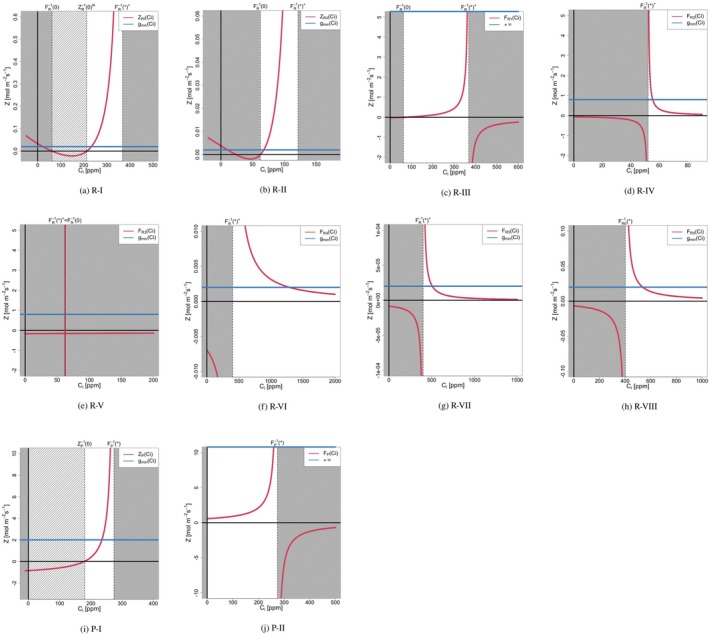
Geometry of $Z\left(C_{i}\right)$ and $Z_{c}$. Regions outside the possible range of appropriate solutions are shaded in grey, while those additionally excluded by the modified range are hatched. $F_{R}^{-1}\left(0\right)$ represents the zero of $F_{R1}\left(C_{i}\right)$ through $F_{R4}\left(C_{i}\right)$ (Equation (S93)), while $F_{R}^{-1}{\left(*\right)}^{+}$ and $F_{R}^{-1}{\left(*\right)}^{-}$ represent the singularities of these same functions (Equation (S86)). Additionally, $F_{R0}^{-1}\left(*\right)$ and $F_{P}^{-1}\left(*\right)$ denote the singularities of $F_{R0}\left(C_{i}\right)$ and $F_{P}\left(C_{i}\right)$, respectively (Equations S120 and S136).

For cases [R‐I, II, IV, VI to VIII, and P‐I], $Z\left(C_{i}\right)=Z_{c}\Leftrightarrow Z\left(C_{i}\right)=g_{\min}$ (Tables 6, 7, 8). At one end of the possible range of appropriate solutions, $Z\left(C_{i}\right)$ is zero or approaches zero, while at the other end, $Z\left(C_{i}\right)$ approaches +∞ (Tables 6, 7, 8). Note that the modified possible range of appropriate solutions is applied for cases [R‐I] and [P‐I] (Tables 6, 7, 8). Since $g_{\min}>0$, $Z\left(C_{i}\right)$ satisfies $Z\left(C_{i}\right)<g_{\min}$ at one end and $Z\left(C_{i}\right)>g_{\min}$ at the other end. Furthermore, within this range, $Z\left(C_{i}\right)$ is either monotonically increasing or monotonically decreasing, depending on the inequality between $Z\left(C_{i}\right)$ and $g_{\min}$ at both ends (Tables 6, 7, 8). Based on these geometric characteristics, cases [R‐I, II, IV, VI to VIII, and P‐I] can be divided into two distinct geometric groups. Table 9 summarizes these groups. From the geometric properties of both groups, we find that $Z\left(C_{i}\right)$ and $g_{\min}$ intersect exactly once within the range of appropriate solutions. Therefore, we have proven that there is always a unique appropriate solution for cases [R‐I, II, IV, VI to VIII, and P‐I]. Figure 4a,b,d,f through i illustrate the single intersection between $Z\left(C_{i}\right)$ and $Z_{c}$ within the possible range of appropriate solutions for these cases.

**TABLE 9:** $Z\left(C_{i}\right)$ and $Z^{\prime }\left(C_{i}\right)$ for cases [R‐I, II, IV, VI to VIII, and P‐I].

Group	Left end	Right end	$Z^{\prime }\left(C_{i}\right)$
R‐I, II, and P‐I	$Z\left(C_{i}\right)<g_{\min}$	$Z\left(C_{i}\right)>g_{\min}$	$Z^{\prime }\left(C_{i}\right)>0$
R‐IV, VI, VII, and VIII	$Z\left(C_{i}\right)>g_{\min}$	$Z\left(C_{i}\right)<g_{\min}$	$Z^{\prime }\left(C_{i}\right)<0$

For [R‐V], $Z\left(C_{i}\right)=Z_{c}\Leftrightarrow F_{R}\left(C_{i}\right)=g_{\min}$. According to Table 4, the only possible solution is $C_{i}=F_{R}^{-1}{\left(*\right)}^{+}=F_{R}^{-1}\left(0\right)$. However, at $C_{i}=F_{R}^{-1}{\left(*\right)}^{+}=F_{R}^{-1}\left(0\right)$, $F_{R}\left(C_{i}\right)$ is indeterminate. Therefore, special treatment is required for [R‐V]. In this case, if the only possible solution is substituted into the model equations (Equations (1), (2), (3), (4), (5), (6), (7), (8), (9), (10), (11), (12)) and all other internal variables are thereby uniquely determined, the possible solution is regarded as the unique solution. Specifically, substituting $C_{i}=C_{s}=C_{a}=F_{R}^{-1}\left(0\right)=A_{\mathrm{nR}}^{-1}\left(0\right)$ into the model yields $g_{s}=g_{\min}=Z_{c}$, together with $e_{s}=g_{\min}e_{s}+g_{b}e_{a}/g_{\min}+g_{b}$ (for $e_{a}<e_{i}$) or $e_{s}=e_{i}$ (for $e_{a}\geq e_{i}$), both of which are uniquely determined. Therefore, $C_{i}=F_{R}^{-1}{\left(*\right)}^{+}=F_{R}^{-1}\left(0\right)$ represents the unique solution for [R‐V]. Thus, we have proven that there is always a unique solution for [R‐V]. Figure 4e illustrates the single intersection between $Z\left(C_{i}\right)$ and $Z_{c}$ at the only possible solution for this case.

For the remaining cases [R‐III] and [P‐II], $Z\left(C_{i}\right)=Z_{c}\Leftrightarrow F_{R}\left(C_{i}\right)=+\infty$ for [R‐III] and $F_{P}\left(C_{i}\right)=+\infty$ for [P‐II], as indicated in Tables 6, 7, 8. The only $C_{i}$ that satisfies $F_{R}\left(C_{i}\right)=+\infty$ or $F_{P}\left(C_{i}\right)=+\infty$ within the range of $C_{i}>0$ corresponds to their respective singularities: $F_{R}^{-1}{\left(*\right)}^{+}$ for [R‐III] and $F_{P}^{-1}\left(*\right)$ for [P‐II], as shown in Figure 4c,j. Thus, $F_{R}^{-1}{\left(*\right)}^{+}$ for [R‐III] and $F_{P}^{-1}\left(*\right)$ for [P‐II] are the unique solutions for these cases. Therefore, we have proven that there is always a unique appropriate solution for the remaining cases [R‐III] and [P‐II].

It is important to note that $F_{R}\left(C_{i}\right)$ and $F_{P}\left(C_{i}\right)$ are strictly mathematically undefined at their respective singularities. These singular points nevertheless serve as solutions because Medlyn's stomatal conductance equation diverges when the vapor pressure deficit is zero (D=0) (Equation 4). To address this, stomatal resistance $r_{s}$, expressed as the inverse of $g_{s}$, can be used when D=0. By using $r_{s}$, we rigorously mathematically obtain the unique solution $C_{i}=C_{s}=F_{R}^{-1}{\left(*\right)}^{+}$ or $F_{P}^{-1}\left(*\right)$ from Equations (6), (7), (9), and (18) when D=0 and $r_{s}=0$. Thus the mathematically rigorous solution obtained using $r_{s}$ is identical to the solution obtained through the less rigorous treatment that directly considers $g_{s}$ at D=0 for the cases [R‐III] and [P‐II].

Thus, as demonstrated above, for all cases, there is always exactly one solution satisfying $Z\left(C_{i}\right)=Z_{c}$ within the possible range of appropriate solutions. Consequently, the Theorem 1 is proven.

## 4 Discussion and Challenges

### 4.1 The Existence and Uniqueness of Solutions

In this study, we mathematically proved the existence and uniqueness of biologically and physically appropriate solutions for the $A_{n}$–E–$g_{s}$ model, which is explicitly described in Sellers et al. (1992) and represents a more comprehensive form of the $A_{n}$–$g_{s}$ model (Collatz et al. 1991), a standard model for estimating leaf photosynthesis and stomatal conductance. This theorem resolves the longstanding problem of existence and uniqueness of solutions, which has persisted for over 30 years since the model was proposed: Does a solution always exist? And even if a solution is obtained, can it be guaranteed to be the correct one among potentially multiple mathematical solutions–specifically, the one actually realized in nature?

The theorem guarantees both existence and uniqueness. By proving existence, it ensures that a biologically and physically meaningful solution always exists, allowing numerical iterative algorithms to be safely applied. In the absence of such a guarantee, convergence failures could not be distinguished from the non‐existence of solutions. Indeed, several studies have reported that numerical solutions do not always converge (Baldocchi 1994; Sun et al. 2012). With this proof, any difficulty in convergence can now be attributed to the numerical algorithm.

By proving uniqueness, the theorem shows that there is only one solution that satisfies the criteria (i.e., $C_{i}>0$ and $g_{s}>0$). Thus, if a numerical algorithm yields a solution that meets these conditions, that solution can be confidently regarded as the correct one. Without this assurance, it would have been impossible to rule out the existence of another solution that could actually be realized in nature, meaning that the validity of solutions obtained in past or future studies could not be guaranteed.

Past estimates obtained using the $A_{n}$–E–$g_{s}$ model that satisfy these conditions can now be retrospectively assessed. We recommend verifying whether these criteria were met in prior model applications. Given that many past estimates likely satisfied these conditions, they were unlikely to be in error. Nevertheless, the significance of this theorem remains, because even if criteria such as $C_{i}>0$ and $g_{s}>0$ were confirmed in earlier studies, there had previously been no guarantee that the selected solution was the correct one. This study is the first to provide a formal mathematical assurance that the solution satisfying these criteria is indeed the correct one. Similarly, future studies can ensure the validity of their results by applying these criteria. Accordingly, this study's theorem will have significant benefits for research in related fields, spanning from past to future endeavors.

The criteria proposed in this study consist of two conditions, $C_{i}>0$ and $g_{s}>0$, both of which are required to ensure the existence and uniqueness of solutions. Furthermore, depending on environmental conditions, the admissible range may be further restricted. The necessity of these conditions and the possible restrictions on the admissible range are discussed in the Appendix S1: SI7.

Resolving the existence and uniqueness problem has far‐reaching implications for a broad range of fields that rely on the $A_{n}$–E–$g_{s}$ model, including plant, ecosystem, agriculture, hydrology, climate, and Earth system sciences (Table 1) and (Foley et al. 1996; Krinner et al. 2005; Longo et al. 2019; Lawrence et al. 2019; Masutomi, Ono, Mano, et al. 2016; Masutomi, Ono, Takimoto, et al. 2016; Takata et al. 2003; Zhang et al. 1996; Tatebe et al. 2019; Watanabe et al. 2011; Danabasoglu et al. 2020; Arora 2002). It enhances the model's reliability for estimating fundamental leaf processes in terrestrial plants, which are critical to understanding global carbon and water cycles (Friedlingstein et al. 2022; Jasechko et al. 2013; Schlesinger and Jasechko 2014), assessing climate change and its impacts, and improving the management of agricultural and natural resources.

### 4.2 Challenges

The $A_{n}$–E–$g_{s}$ has several variants, with numerous models proposed, such as those for stomatal conductance (Damour et al. 2010), for TPU‐limited photosynthesis (von Caemmerer 2000; Busch et al. 2018), and for integrated approaches incorporating an energy balance model (Nikolov et al. 1995). Proving the existence and uniqueness of solutions for variants other than the one examined in this study remains a significant challenge. This study specifically provides a proof for one variant. Nevertheless, many variants of the $A_{n}$–E–$g_{s}$ model share common structural components. In particular, the photosynthesis submodel is typically based on the Farquhar model, and the diffusion processes of CO_2_ and H_2_O are formulated in a similar manner across different variants. In such cases, the function $F\left(C_{i}\right)$ remains essentially the same, while the function $G\left(C_{i}\right)$ varies depending on the choice of stomatal conductance model. This suggests that the analytical framework introduced in this study, based on the functions $F\left(C_{i}\right)$ and $G\left(C_{i}\right)$, can be extended to a wide range of model variants. In many cases, this framework is expected to enable the proof of existence and uniqueness of solutions, although the specific form of $G\left(C_{i}\right)$ may require case‐by‐case analysis.

If the existence and uniqueness of solutions cannot be demonstrated for a variant, it may indicate that the model requires additional criteria or considerations. For example, under TPU‐limited conditions, a more complex alternative model–which accounts for glycolate carbon leaving the photorespiratory pathway (von Caemmerer 2000, Busch et al. 2018)–yields no solutions under certain environmental conditions (see Appendix S1: SI8). From a broader perspective, identifying a general form of models that guarantees the existence and uniqueness of solutions is an important challenge. Once identified, such a general form could impose constraints on models and clarify whether modifications to equations or additional conditions are necessary for the many existing models.

In this study, leaf temperature ($T_{l}$) is not explicitly treated as an internal variable. In practice, $\Gamma^{*}$, $K_{c}$, $K_{o}$, $R_{L}$, $V_{\text{cmax}}$, and $T_{p}$ are all functions of $T_{l}$. To include $T_{l}$ as an internal variable, it would be necessary to add an independent equation–namely, an energy balance model (EBM)–to determine $T_{l}$. A fundamental challenge with this integrated model lies in the mathematical proof of the existence and uniqueness of solutions. As in the case of the $A_{n}$–E–$g_{s}$ model, unless the existence and uniqueness of solutions are mathematically guaranteed, the numerical iterative algorithm for obtaining solutions may fail to converge or the solution obtained may not be valid. Establishing such a proof would require a considerably more extensive mathematical derivation and therefore remains an important challenge for future research.

As pointed out in Masutomi (2023), it has been recently understood that nocturnal stomatal conductance is not constantly minimum assumed in this study (Equation 4) and seems to be correlated with respiration rate (Yu et al. 2019; Wang et al. 2021; Zhang et al. 2021). However, the relationship with environmental factors such as humidity and CO_2_ during nighttime is not well understood, and the final model equation incorporating these environmental variables remains unknown. Once this becomes clear, proving the existence and uniqueness of solutions for the $A_{n}$–E–$g_{s}$ model incorporating such nocturnal stomatal conductance models will become an important future challenge.

As described in the Appendix S1, the proof presented in this study is highly lengthy and complex. While we expect that a simpler proof exists, we were unable to find one. This challenge remains unresolved and will be passed on to the next generation of researchers to tackle.

This study focused on C3 plants, but similar research could be conducted for C4 plants. For C4 plants, using relatively simple models (Collatz et al. 1992; Lawrence et al. 2019) might not be overly challenging for this proof. However, if more complex models like those proposed by Yin and Struik (2009) are used, the task might be somewhat more difficult. Nevertheless, the approach for C3 plants has been established, so it is likely only a matter of time before this is also proven for C4 plants. If this cannot be proven for C4 plants, conversely, it would be a very intriguing finding.

In this study, we focused on the $A_{n}$–E–$g_{s}$ model, which deals with only steady‐state photosynthesis, transpiration, and stomatal conductance. However, more general models that describe non‐steady states can also be considered. These models are expected to be formulated as dynamic systems of differential equations involving time, offering a much richer and more intricate landscape. Nevertheless, in the real world, a single value is ultimately selected at a time, and the concept of existence and uniqueness of solutions demonstrated in this study undoubtedly remains important in such dynamic systems of differential equations as well.

## Author Contributions

**Yuji Masutomi:** conceptualization, methodology, funding acquisition, writing – original draft, writing – review and editing, formal analysis, validation, visualization, investigation, software, resources, project administration, data curation. **Kazuhiko Kobayashi:** supervision, writing – review and editing, conceptualization.

## Funding

This study was partly supported by the Environment Research and Technology Development Fund (JPMEERF21S12020 and JPMEERF20252002) of the Environmental Restoration and Conservation Agency (ERCA), and JSPS KAKENHI Grant Number JP23H00351.

## Conflicts of Interest

The authors declare no conflicts of interest.

## Supporting information


**Appendix S1:** gcb70974‐sup‐0001‐supinfo.pdf.

## Data Availability

This study is based on theoretical and mathematical analyses and did not generate, analyze, or use any datasets.
