# Supplementary material for: The Existence and Uniqueness of Solutions in the Leaf Photosynthesis‐Transpiration‐Stomatal Conductance Model for C3 Plants
Source: Glob Chang Biol. 2026 Jul 24;32(7):e70974. doi: 10.1111/gcb.70974 (PMC13397073; doi:10.1111/gcb.70974)
Supplement: Supplementary file 1 — Appendix S1: gcb70974‐sup‐0001‐supinfo.pdf. [file GCB-32-e70974-s001.pdf]

Supplementary Information for

Yuji Masutomi*, Kazuhiko Kobayashi

*Corresponding author. Email: masutomi.yuji@nies.go.jp

Contents of Supplementary Information

SI 0 **Mathematical notations and conventions** **S8**

SI 1 $A_{nR}(C_i)\,[a > 0,$ **Rubisco/RuBP-limited**$]$ **S9**

SI 2 $F_R(C_i)\,[a > 0,$ **Rubisco/RuBP-limited**$]$ **S14**

SI 2.1 Expression of $F_R(C_i)$ S14

SI 2.2 $H_R(C_i)$ S14

SI 2.3 $H'_R(C_i)$ S21

SI 2.4 $F_R(C_i)$ S22

SI 2.5 $F'_R(C_i)$ S30

SI 2.5.1 $\tilde{H}_R(C_i)$ S30

SI 2.5.2 Sign of $F'_R(C_i)$ S32

SI 3 $F_{R0}(C_i)$ $[a = 0$**, Rubisco/RuBP-limited]** **S34**

SI 4 $F_P(C_i)$ **[TPU-limited]** **S36**

SI 4.1 $H_P(C_i)$ S36

SI 4.2 $F_P(C_i)$ S37

SI 5 $Z_R(C_i)$ **[C1,** $e_a < e_i$**]** **S39**

SI 5.1 $I_R(C_i)$ S39

SI 5.2 $K_R(C_i)$ S47

SI 5.3 $N_R(C_i) = 0$ S52

SI 5.3.1 $L_R(C_i)$ S52

SI 5.3.2 $M_R(C_i)$ S53

SI 5.3.3 Sign of $L_R(C_i)$ and $M_R(C_i)$ S55

SI 5.4 $\tilde{N}_R(C_i)$ S55

SI 5.4.1 $\tilde{L}_R(C_i)$ S56

SI 5.4.2 $\tilde{L}'_R(C_i)$ S57

SI 5.4.3 $\tilde{L}''_R(C_i)$ S59

SI 5.4.4 $\tilde{L}'''_R(C_i)$ S61

SI 5.4.5 $\tilde{M}_R(C_i)$ . . . S63
SI 5.4.6 $\tilde{M}'_R(C_i)$ . . . S72
SI 5.4.7 $\tilde{M}''_R(C_i)$ . . . S80
SI 5.4.8 $\tilde{M}'''_R(C_i)$ . . . S83
SI 5.4.9 $\tilde{L}'_R(C_i)$ and $\tilde{M}'_R(C_i)$ . . . S84
SI 5.4.10 $\tilde{N}_R(C_i) = \tilde{L}_R(C_i) - \tilde{M}_R(C_i) = 0$ . . . S95
SI 5.5 $N_R(C_i)$ . . . S108
SI 5.6 $N'_R(C_i)$ and $N''_R(C_i)$ . . . S111
SI 5.7 $Z_R(C_i)$ . . . S118
SI 5.8 $Z'_R(C_i)$ . . . S120
SI 5.8.1 $X_{R1}(C_i)$ . . . S120
SI 5.8.2 $X_{R2}(C_i)$ . . . S121
SI 5.8.3 $\tilde{X}_{R1}(C_i)$ . . . S121
SI 5.8.4 $\tilde{X}'_{R2}(C_i)$ . . . S121
SI 5.8.5 $\tilde{X}_{R2}(C_i)$ . . . S122
SI 5.8.6 Sign of $Z'_R(C_i)$ . . . S127

SI 6 $Z_P(C_i)$ [TPU-limited] S128
SI 6.1 $I_P(C_i)$ . . . S128
SI 6.2 $N_P(C_i)$ . . . S128
SI 6.3 $Z_P(C_i)$ . . . S130
SI 6.4 $Z'_P(C_i)$ . . . S131

SI 7 Two criteria $C_i > 0$ and $g_s > 0$ S132

SI 8 Non-existence of Solutions for $A_{np}(C_i)$ S133

List of Figures

S1 $H_R(C_i)$ [C1] . . . S20
S2 $I_R(C_i)$ [C1, $\tilde{\gamma} < 0$] . . . S43
S3 $K_R(C_i)$ for [C1, $\phi < 0$] . . . S49
S4 $K_R(C_i)$ for [C1, $\phi > 0$] . . . S50
S5 $K_R(C_i)$ for [C1, $\phi = 0$] . . . S51
S6 $L_R(C_i)$ . . . S53
S7 $M_R(C_i)$ [C1, $\tilde{\gamma} < 0$] . . . S54
S8 $\tilde{L}_R(C_i)$ . . . S57
S9 $\tilde{L}'_R(C_i)$ . . . S59
S10 $\tilde{L}''_R(C_i)$ . . . S61
S11 $\tilde{L}'''_R(C_i)$ . . . S63
S12 $\tilde{M}_R(C_i)$ [C1, $I_R^{-1}(0)^S < 0$] . . . S66
S13 $\tilde{M}_R(C_i)$ [C1, $I_R^{-1}(0)^S > 0$] . . . S67
S14 $\tilde{M}_R(C_i)$ [C1, $I_R^{-1}(0)^S = 0$] . . . S68
S15 $\tilde{M}'_R(C_i)$ [C1, $A_{nR}^{-1}(0) + \frac{\eta}{4} > 0$] . . . S74
S16 $\tilde{M}'_R(C_i)$ [C1, $A_{nR}^{-1}(0) + \frac{\eta}{4} < 0$] . . . S75
S17 $\tilde{M}'_R(C_i)$ [C1, $A_{nR}^{-1}(0) + \frac{\eta}{4} = 0$] . . . S76
S18 $\nu(C_a)$ . . . S80
S19 $\tilde{L}_R(C_i)$ and $\tilde{M}_R(C_i)$ for [P1] . . . S97
S20 $\tilde{N}_R(C_i)$ for [P1] . . . S98
S21 $\tilde{L}_R(C_i)$ and $\tilde{M}_R(C_i)$ for [P2] . . . S99
S22 $\tilde{N}_R(C_i)$ for [P2] . . . S100
S23 $\tilde{L}_R(C_i)$ and $\tilde{M}_R(C_i)$ for [P3] . . . S103
S24 $\tilde{N}_R(C_i)$ for [P3] . . . S104
S25 $\tilde{L}_R(C_i)$ and $\tilde{M}_R(C_i)$ for [P4] . . . S105
S26 $\tilde{N}_R(C_i)$ for [P4] . . . S106
S27 $\tilde{N}_R(C_i)$ at $-100 < C_i < 200$ for [P4] . . . S107
S28 $N_R(C_i)$ for [ZI] . . . S110

S29 $N_R(C_i)$ for [ZII] . . . S111
S30 $N''_R(C_i)$ for [ZI] . . . S113
S31 $N''_R(C_i)$ for [ZII] . . . S114
S32 $N'_R(C_i)$ for [ZI] . . . S117
S33 $N'_R(C_i)$ for [ZII] . . . S118

List of Tables

S1 Five patterns of conditions and inequality relations S11
S2 Sign chart for $A_{nR1}(C_i)$ S12
S3 Sign chart for $A_{nR2}(C_i)$ S12
S4 Sign chart for $A_{nR3}(C_i)$ S12
S5 Sign chart for $A_{nR4}(C_i)$ S12
S6 Sign chart for $A_{nR5}(C_i)$ S13
S7 Inequalities for $H_R(C_i)$ S19
S8 Sign chart of $H_R(C_i)$ [C1] S20
S9 Sign chart of $H_R(C_i)$ [C2] S20
S10 Sign chart of $H_R(C_i)$ [C3] S20
S11 Sign chart of $H_R(C_i)$ [C4] S21
S12 Sign chart of $H_R(C_i)$ [C5] S21
S13 Sign chart of $F_{R1}(C_i)$ S25
S14 Sign chart of $F_{R2}(C_i)$ S26
S15 Sign chart of $F_{R3}(C_i)$ S27
S16 Sign chart of $F_{R4}(C_i)$ S28
S17 Sign chart of $F_{R5}(C_i)$ S29
S18 Possible range of appropriate solutions for $F_R(C_i)$ S30
S19 Sign chart of $F_{R0}(C_i)$ S35
S20 Possible range of appropriate solutions for $F_{R0}(C_i)$ S35
S21 Geometry of $F_P(C_i)$ S38
S22 Possible range of appropriate solutions for $F_P(C_i)$ S38
S23 Sign Chart of $I_R(C_i)$ under [C1, $\tilde{\gamma} < 0$] S44
S24 Sign Chart of $I_R(C_i)$ under [C1, $\tilde{\gamma} > 0$] S45
S25 Sign Chart of $I_R(C_i)$ under [C1, $\tilde{\gamma} = 0$] S46
S26 Sign Chart of $K_R(C_i)$ [C1, $\phi < 0$] S51
S27 Sign Chart of $K_R(C_i)$ [C1, $\phi > 0$] S51
S28 Sign Chart of $K_R(C_i)$ [C1, $\phi = 0$] S51

S29 Sign chart of $L_R(C_i)$ S53
S30 Sign chart of $M_R(C_i)$ [C1] S55
S31 Sign chart of $\tilde{L}_R(C_i)$ S57
S32 Sign chart of $\tilde{L}'_R(C_i)$ S59
S33 Sign chart of $\tilde{L}''_R(C_i)$ S61
S34 Sign chart of $\tilde{L}'''_R(C_i)$ S63
S35 Sign chart of $\tilde{M}_R(C_i)$ [C1, $I_R^{-1}(0)^S < 0$] S69
S36 Sign chart of $\tilde{M}_R(C_i)$ [C1, $I_R^{-1}(0)^S > 0$] S70
S37 Sign chart of $\tilde{M}_R(C_i)$ [C1, $I_R^{-1}(0)^S = 0$] S71
S38 Sign chart of $\tilde{M}'_R(C_i)$ [C1, $A_{nR}^{-1}(0) + \frac{\eta}{4} > 0$] S76
S39 Sign chart of $\tilde{M}'_R(C_i)$ [C1, $A_{nR}^{-1}(0) + \frac{\eta}{4} < 0$] S76
S40 Sign chart of $\tilde{M}'_R(C_i)$ [C1, $A_{nR}^{-1}(0) + \frac{\eta}{4} = 0$] S76
S41 Four patterns for $\tilde{L}_R(C_i)$ and $\tilde{M}_R(C_i)$ S95
S42 The number of intersections between $\tilde{L}_R(C_i)$ and $\tilde{M}_R(C_i)$ at $A_{nR}^{-1}(0) < C_i < H_R^{-1}(0)$ S107
S43 Sign chart of $N_R(C_i)$ for ZI S111
S44 Sign chart of $N_R(C_i)$ for ZII S111
S45 Sign chart of $Z_R(C_i)$ for [ZI] S119
S46 Sign chart of $Z_R(C_i)$ for [ZII] S120

SI 0 Mathematical notations and conventions

In this study, parenthesis "()" is used for denoting the argument of functions. For example, a function "F" of C_i is expressed as $F(C_i)$. When an inequality is used as the argument of a function, it represents a condition. For example, $F(a < C_i < b) < 0$ means that $F(C_i) < 0$ for $a < C_i < b$. Curly brackets "{}" is used for separating a group of equations, such as $a\{b + c\}$ indicating $ab + ac$. Square brackets "[ ]" is used for expressing conditions under which the respective equation is valid.

Suppose that $\mathcal{F}(C_i)$ is a function of C_i. The following notations are used in the present study

$$\mathcal{F}'(C_i) \quad : \quad \text{the derivative of } \mathcal{F}(C_i) \text{ with respect to } C_i \tag{S1}$$

$$\mathcal{F}^{-1}(C_i) \quad : \quad \text{the inverse function of } \mathcal{F}(C_i) \tag{S2}$$

$$\mathcal{F}^{-1}(0) \quad : \quad \text{the roots of } \mathcal{F}(C_i) \tag{S3}$$

$$\mathcal{F}^{-1}(*) \quad : \quad \text{the singularities of } \mathcal{F}(C_i) \tag{S4}$$

We use the following definition for singularity: C_i satisfying $\mathcal{F}(C_i) = \infty, -\infty$, or $0/0$ at $-\infty < C_i < \infty$. The superscripts "+" and "−" attached to $\mathcal{F}^{-1}(0)$ and $\mathcal{F}^{-1}(*)$, such as $\mathcal{F}^{-1}(0)^+$ and $\mathcal{F}^{-1}(*)^-$, denote positive and negative values, respectively.

SI 1 $A_{nR}(C_i)[a > 0,$ Rubisco/RuBP-limited]

In this chapter, we examine the geometry of $A_{nR}(C_i)$, which is defined by Eq. (13) as follows:

$$A_{nR}(C_i) = \frac{a\{C_i - \Gamma^*\}}{bC_i + c} - R_L \quad [a > 0] \tag{S5}$$

(a) Derivative: $A'_{nR}(C_i)$

By differentiating Eq. (S5) with respect to C_i, we obtain

$$A'_{nR}(C_i) = \frac{a\{b\Gamma^* + c\}}{\{bC_i + c\}^2} \quad (> 0) \tag{S6}$$

Therefore, $A_{nR}(C_i)$ increases monotonically with C_i except at the singularity derived in the next section.

(b) Singularity: $A_{nR}^{-1}(*)$

From Eq. (S5), the singularity is given by:

$$A_{nR}^{-1}(*) = -\frac{c}{b} \quad (< 0) \tag{S7}$$

To clarify the signs of infinity on both sides of the singularity, an infinitesimal positive quantity, "$\epsilon(> 0)$", is introduced. Using "ϵ", the neighborhoods on both sides of $A_{nR}^{-1}(*)$ are expressed as:

$$A_{nR}(A_{nR}^{-1}(*) \pm \epsilon) = \mp\infty \tag{S8}$$

(c) Root: $A_{nR}^{-1}(0)$

Setting $A_{nR}(C_i) = 0$ yields

$$A_{nR}^{-1}(0) = \frac{a\Gamma^* + cR_L}{a - bR_L} \tag{S9}$$

Therefore,

$$A_{nR}^{-1}(0) \begin{cases} > 0 & [a > 0, a - bR_L > 0] \\ < 0 & [a > 0, a - bR_L < 0] \\ \text{not exist} & [a > 0, a - bR_L = 0] \end{cases} \tag{S10}$$

(d) $A_{nR}(\pm\infty)$

From Eq. (S5), $A_{nR}\ (\pm\infty)$ is given as:

$$A_{nR}\ (\pm\infty) = \frac{a - bR_L}{b} \tag{S11}$$

Therefore,

$$A_{nR}\,(\pm\infty) \begin{cases} > 0 & [a > 0, a - bR_L > 0] \\ < 0 & [a > 0, a - bR_L < 0] \\ = \mp 0 & [a > 0, a - bR_L = 0] \end{cases} \tag{S12}$$

Eq. (S12) leads to:

$$\mathrm{sgn}(A_{nR}(\pm\infty)) = \mathrm{sgn}(a - bR_L) \quad [a > 0] \tag{S13}$$

(e) Inequality relations among $A_{nR}^{-1}(0)$, $A_{nR}^{-1}(*)$, C_a, and 0

In the case where $a - bR_L > 0$, Eqs. (S7) and (S10) yield the following inequality:

$$A_{nR}^{-1}(*) < 0 < A_{nR}^{-1}(0) \quad [a > 0,\ a - bR_L > 0] \tag{S14}$$

Thus, when $a > 0$, $a - bR_L > 0$ and $C_a > A_{nR}^{-1}(0)$, we have

$$A_{nR}^{-1}(*) < 0 < A_{nR}^{-1}(0) < C_a \quad [a > 0,\ a - bR_L > 0 \text{ and } C_a > A_{nR}^{-1}(0)]. \tag{S15}$$

Similarly, when $a > 0$, $a - bR_L > 0$ and $C_a < A_{nR}^{-1}(0)$, or when $a > 0$, $a - bR_L > 0$ and $C_a = A_{nR}^{-1}(0)$, we have

$$A_{nR}^{-1}(*) < 0 \le C_a < A_{nR}^{-1}(0) \quad [a > 0,\ a - bR_L > 0 \text{ and } C_a < A_{nR}^{-1}(0)], \tag{S16}$$

$$A_{nR}^{-1}(*) < 0 \le C_a = A_{nR}^{-1}(0) \quad [a > 0,\ a - bR_L > 0 \text{ and } C_a = A_{nR}^{-1}(0)], \tag{S17}$$

In the case where $a > 0$ and $a - bR_L < 0$, $A_{nR}(-\infty)$ is negative (Eq. (S12)), while $A_{nR}(A_{nR}^{-1}(*) - \epsilon)$ is positive (Eq. (S8)), indicating opposite signs at these two points. Since $A_{nR}(C_i)$ increases monotonically with C_i (Eq. (S6)), $A_{nR}^{-1}(0)$ must exist between $-\infty$ and $A_{nR}^{-1}(*)$. By combining this with $C_a \ge 0$, we have

$$A_{nR}^{-1}(0) < A_{nR}^{-1}(*) < 0 \le C_a \quad [a > 0,\ a - bR_L < 0] \tag{S18}$$

In the case where $a > 0$ and $a - bR_L = 0$, Eqs. (S7) and (S10), combined with $C_a \ge 0$, yield

$$A_{nR}^{-1}(*) < 0 \le C_a \quad [a > 0,\ a - bR_L = 0]. \tag{S19}$$

In summary, there are five patterns of inequality relations among $A_{nR}^{-1}(0)$, $A_{nR}^{-1}(*)$, C_a, and 0 (Eqs. (S15) to (S19)). Table 3 summarizes these five patterns of inequality relations and

the associated conditions. For the conditions, $A_{nR}(\pm\infty)$ is used instead of $a - bR_L$ since the signs are identical under [$a > 0$] (Eq. (S13)). To distinguish the conditions, the symbols [C1] to [C5] are used hereafter.

Table S1: Five patterns of conditions and inequality relations

Case	Condition	Inequality relation
[C1]	$a > 0$, $A_{nR}(\pm\infty) > 0$, $C_a - A_{nR}^{-1}(0) > 0$	$A_{nR}^{-1}(*) < 0 < A_{nR}^{-1}(0) < C_a$
[C2]	$a > 0$, $A_{nR}(\pm\infty) > 0$, $C_a - A_{nR}^{-1}(0) < 0$	$A_{nR}^{-1}(*) < 0 \le C_a < A_{nR}^{-1}(0)$
[C3]	$a > 0$, $A_{nR}(\pm\infty) > 0$, $C_a - A_{nR}^{-1}(0) = 0$	$A_{nR}^{-1}(*) < 0 < C_a = A_{nR}^{-1}(0)$
[C4]	$a > 0$, $A_{nR}(\pm\infty) < 0$	$A_{nR}^{-1}(0) < A_{nR}^{-1}(*) < 0 \le C_a$
[C5]	$a > 0$, $A_{nR}(\pm\infty) = 0$	$A_{nR}^{-1}(*) < 0 \le C_a$

(f) $A_{nR}(0)$

From Eq. (S5), $A_{nR}(0)$ is given by:

$$A_{nR}(0) = -\frac{a\Gamma^* + cR_L}{c}(< 0) \tag{S20}$$

(g) Expression of $A_{nR}(C_i)$

Using $A_{nR}^{-1}(0)$, $A_{nR}^{-1}(*)$, and $A_{nR}(0)$, $A_{nR}(C_i)$ can be represented as follows:

$$A_{nR}(C_i) = \begin{cases} \frac{A_{nR}(\pm\infty)\{C_i - A_{nR}^{-1}(0)\}}{C_i - A_{nR}^{-1}(*)} & \text{[C1 to C4]} \\ \frac{A_{nR}(0)}{C_i - A_{nR}^{-1}(*)} & \text{[C5]} \end{cases} \tag{S21}$$

(h) $A_{nR}(C_a)$

From Eq. (S5), $A_{nR}(C_a)$ is given by:

$$A_{nR}(C_a) = \frac{a(C_a - \Gamma^*)}{bC_a + c} - R_L = \begin{cases} \frac{A_{nR}(\pm\infty)\{C_a - A_{nR}^{-1}(0)\}}{C_a - A_{nR}^{-1}(*)} & \text{[C1 to C4]} \\ \frac{A_{nR}(0)}{C_a - A_{nR}^{-1}(*)} & \text{[C5]} \end{cases} \tag{S22}$$

By using the signs of $A_{nR}(\pm\infty)$, $A_{nR}^{-1}(0)$, and $A_{nR}(0)$ (Eqs. (S12), (S10), and (S20)), along with the inequalities for each case (Table 3), we can determine the signs of $A_{nR}(C_a)$ as

follows:

$$A_{nR}(C_a)\begin{cases} > 0 & [\text{C1}] \\ = 0 & [\text{C3}] \\ < 0 & [\text{C2, C4 and C5}] \end{cases} \tag{S23}$$

(i) Geometry of $A_{nR}(C_i)$

According to cases [C1] to [C5], $A_{nR}(C_i)$ exhibits distinct geometries for each case. To differentiate these, we denote them as $A_{nR1}(C_i)$ to $A_{nR5}(C_i)$ for cases [C1] to [C5], respectively. Figures 1a to 1e display the curves of $A_{nR1}(C_i)$ to $A_{nR5}(C_i)$, and Tables S2 to S6 provide the sign charts of $A_{nR1}(C_i)$ to $A_{nR5}(C_i)$.

Table S2: Sign chart for $A_{nR1}(C_i)$

C_i	$-\infty$		$A_{nR}^{-1}(*)$		0		$A_{nR}^{-1}(0)$		C_a		$+\infty$
$A_{nR1}(C_i)$	+	+	$+\infty, -\infty$	-	-	-	0	+	+	+	+

Table S3: Sign chart for $A_{nR2}(C_i)$

C_i	$-\infty$		$A_{nR}^{-1}(*)$		0		C_a		$A_{nR}^{-1}(0)$		$+\infty$
$A_{nR2}(C_i)$	+	+	$+\infty, -\infty$	-	-	-	-	-	0	+	+

Table S4: Sign chart for $A_{nR3}(C_i)$

C_i	$-\infty$		$A_{nR}^{-1}(*)$		0		$C_a = A_{nR}^{-1}(0)$		$+\infty$
$A_{nR3}(C_i)$	+	+	$+\infty, -\infty$	-	-	-	0	+	+

Table S5: Sign chart for $A_{nR4}(C_i)$

C_i	$-\infty$		$A_{nR}^{-1}(0)$		$A_{nR}^{-1}(*)$		0		C_a		$+\infty$
$A_{nR4}(C_i)$	-	-	0	+	$+\infty, -\infty$	-	-	-	-	-	-

Table S6: Sign chart for $A_{nR5}(C_i)$

C_i	$-\infty$		$A_{nR}^{-1}(*)$		0		C_a		$+\infty$
$A_{nR5}(C_i)$	+0	+	$+\infty, -\infty$	-	-	-	-	-	-0

SI 2 $F_R(C_i)\,[a > 0,$ Rubisco/RuBP-limited]

We examine the geometry of the function $F_R(C_i)$, which is used when $a > 0$ under Rubisco/RuBP-limited conditions (Eq. (18)). When $a > 0$, it has been shown that there are five different cases, labeled [C1] through [C5] (Table 3). Accordingly, in this chapter, we analyze the geometries of $F_R(C_i)$ for cases [C1] to [C5].

From Eq. (18), the function $F_R(C_i)$ is given by :

$$F_R(C_i) = -\frac{\lambda_s g_b A_{nR}(C_i)}{g_b(C_i - C_a) + \lambda_b A_{nR}(C_i)} \quad [a > 0] \tag{S24}$$

SI 2.1 Expression of $F_R(C_i)$

Using Eq. (S21), $F_R(C_i)$ can be rewritten based on Eq. (S24) as follows:

$$F_R(C_i) = \begin{cases} -\frac{\lambda_s A_{nR}(\pm\infty)\{C_i - A_{nR}^{-1}(0)\}}{H_R(C_i)} & \text{[C1 to C4]} \\ -\frac{\lambda_s A_{nR}(0)}{H_R(C_i)} & \text{[C5]} \end{cases} \tag{S25}$$

where $H_R(C_i)$ is given by a quadratic equation in terms of C_i, as follows:

$$H_R(C_i) = \begin{cases} C_i^2 - \left\{C_a + A_{nR}^{-1}(*) - \lambda_b \frac{A_{nR}(\pm\infty)}{g_b}\right\} C_i + C_a A_{nR}^{-1}(*) - \lambda_b \frac{A_{nR}(\pm\infty)}{g_b} A_{nR}^{-1}(0) & \text{[C1 to C4]} \\ C_i^2 - \left\{C_a + A_{nR}^{-1}(*)\right\} C_i + C_a A_{nR}^{-1}(*) + \lambda_b \frac{A_{nR}(0)}{g_b} & \text{[C5]} \end{cases} \tag{S26}$$

SI 2.2 $H_R(C_i)$

In this section, we investigate $H_R(C_i)$ as defined in Eq. (S26).

(a) Root: $H_R^{-1}(0)$

The equation $H_R(C_i) = 0$ (Eq. (S26)) is a quadratic polynomial in C_i, expressed as $\alpha C_i^2 + \beta C_i + \gamma = 0$, where the coefficients are given by:

$$\alpha = 1 \quad (> 0), \tag{S27}$$

$$\beta = \begin{cases} -\left\{C_a + A_{nR}^{-1}(*) - \lambda_b \frac{A_{nR}(\pm\infty)}{g_b}\right\} & \text{[C1 to C4],} \\ -\left\{C_a + A_{nR}^{-1}(*)\right\} & \text{[C5],} \end{cases} \tag{S28}$$

$$\gamma = \begin{cases} C_a A_{nR}^{-1}(*) - \lambda_b \frac{A_{nR}(\pm\infty)}{g_b} A_{nR}^{-1}(0) & \text{[C1 to C4]}, \\ C_a A_{nR}^{-1}(*) + \lambda_b \frac{A_{nR}(0)}{g_b} & \text{[C5]}. \end{cases} \tag{S29}$$

Table 3 shows $A_{nR}^{-1}(*) < 0$ under [C1] to [C5]; $A_{nR}^{-1}(0) > 0$ and $A_{nR}(\pm\infty) > 0$ under [C1] to [C3]; $A_{nR}^{-1}(0) < 0$ and $A_{nR}(\pm\infty) < 0$ under [C4]; and $A_{nR}(0) < 0$ under [C5] (Eq. (S20)). Based on Eq. (S29), the sign of γ is therefore determined as:

$$\gamma < 0. \tag{S30}$$

Given $\alpha > 0$ (Eq. (S27)) and $\gamma < 0$ (Eq. (S30)), $H_R(C_i) = 0$ has two real roots, one positive and one negative, given by:

$$H_R^{-1}(0)^+ = \frac{-\beta + \sqrt{\beta^2 - 4\alpha\gamma}}{2\alpha} \quad (> 0), \tag{S31}$$

$$H_R^{-1}(0)^- = \frac{-\beta - \sqrt{\beta^2 - 4\alpha\gamma}}{2\alpha} \quad (< 0). \tag{S32}$$

The superscripts \ + " and \ − " denote the positive and negative roots, respectively.

Using $H_R^{-1}(0)^+$ and $H_R^{-1}(0)^-$, $H_R(C_i)$ can be factored as

$$H_R(C_i) = \{C_i - H_R^{-1}(0)^+\}\{C_i - H_R^{-1}(0)^-\}. \tag{S33}$$

From the relationships between roots and coefficients, we obtain

$$H_R^{-1}(0)^+ + H_R^{-1}(0)^- = -\beta, \tag{S34}$$

$$H_R^{-1}(0)^+ H_R^{-1}(0)^- = \gamma \quad (< 0). \tag{S35}$$

Note that $\alpha = 1$ (Eq. (S27)) is assumed in Eqs. (S34) and (S35).

Since $H_R(C_i)$ is a convex quadratic function ($\alpha > 0$ (Eq. (S27))), we have:

$$H_R(C_i < H_R^{-1}(0)^-) > 0, \tag{S36}$$

$$H_R(H_R^{-1}(0)^- < C_i < H_R^{-1}(0)^+) < 0, \tag{S37}$$

$$H_R(H_R^{-1}(0)^+ < C_i) > 0. \tag{S38}$$

(b) $H_R(\pm\infty)$, $H_R(A_{nR}^{-1}(*))$, $H_R(A_{nR}^{-1}(0))$, $H_R(C_a)$, and $H_R(0)$

$$H_R(\pm\infty) = \infty \quad (>0) \quad \text{[C1 to C5]} \tag{S39}$$

$$H_R(A_{nR}^{-1}(*)) = \begin{cases} -\lambda_b \frac{A_{nR}(\pm\infty)}{g_b} \left\{A_{nR}^{-1}(0) - A_{nR}^{-1}(*)\right\} & \text{[C1 to C4]} \\ \lambda_b \frac{A_{nR}(0)}{g_b} \quad (<0) & \text{[C5]} \end{cases} \tag{S40}$$

$$H_R(A_{nR}^{-1}(0)) = \left\{A_{nR}^{-1}(0) - C_a\right\} \left\{A_{nR}^{-1}(0) - A_{nR}^{-1}(*)\right\} \quad \text{[C1 to C4]} \tag{S41}$$

$$H_R(C_a) = \begin{cases} -\lambda_b \frac{A_{nR}(\pm\infty)}{g_b} \left\{A_{nR}^{-1}(0) - C_a\right\} & \text{[C1 to C4]} \\ \lambda_b \frac{A_{nR}(0)}{g_b} \quad (<0) & \text{[C5]} \end{cases} \tag{S42}$$

$$H_R(0) = \gamma \quad (<0) \quad \text{[C1 to C5]} \tag{S43}$$

(c) Inequality relation among $H_R^{-1}(0)^{\pm}$, $A_{nR}^{-1}(*)$, 0, $A_{nR}^{-1}(0)$, and C_a

We examine inequality relations among $H_R^{-1}(0)^{\pm}$, $A_{nR}^{-1}(*)$, 0, $A_{nR}^{-1}(0)$, and C_a for each case from [C1] to [C5].

In case [C1], we have $A_{nR}^{-1}(*) < 0 < A_{nR}^{-1}(0) < C_a$ (Table 3). From Eqs. (S39) to (S43), along with the inequality for case [C1], it follows that:

$$H_R(-\infty) > 0 \quad \text{[C1]}, \tag{S44}$$

$$H_R(A_{nR}^{-1}(*)) < 0 \quad \text{[C1]}, \tag{S45}$$

$$H_R(0) < 0 \quad \text{[C1]}, \tag{S46}$$

$$H_R(A_{nR}^{-1}(0)) < 0 \quad \text{[C1]}, \tag{S47}$$

$$H_R(C_a) > 0 \quad \text{[C1]}, \tag{S48}$$

$$H_R(\infty) > 0 \quad \text{[C1]}. \tag{S49}$$

Since $H_R^{-1}(0)^-$ and $H_R^{-1}(0)^+$ must lie between points where $H_R(C_i)$ changes sign, we derive the following inequality relation:

$$H_R^{-1}(0)^- < A_{nR}^{-1}(*) < 0 < A_{nR}^{-1}(0) < H_R^{-1}(0)^+ < C_a \quad \text{[C1]} \tag{S50}$$

In case [C2], we have $A_{nR}^{-1}(*) < 0 \leq C_a < A_{nR}^{-1}(0)$ (Table 3). From Eqs. (S39) to (S43), along

with the inequality for case [C2], it follows that:

$$H_R(-\infty) > 0 \quad [\text{C2}], \tag{S51}$$

$$H_R(A_{nR}^{-1}(*)) < 0 \quad [\text{C2}], \tag{S52}$$

$$H_R(0) < 0 \quad [\text{C2}], \tag{S53}$$

$$H_R(C_a) < 0 \quad [\text{C2}], \tag{S54}$$

$$H_R(A_{nR}^{-1}(0)) > 0 \quad [\text{C2}], \tag{S55}$$

$$H_R(\infty) > 0 \quad [\text{C2}]. \tag{S56}$$

Following the same reasoning, we obtain the following inequality relation:

$$H_R^{-1}(0)^- < A_{nR}^{-1}(*) < 0 \leq C_a < H_R^{-1}(0)^+ < A_{nR}^{-1}(0) \quad [\text{C2}] \tag{S57}$$

In case [C3], we have $A_{nR}^{-1}(*) < 0 < C_a = A_{nR}^{-1}(0)$ (Table 3). From Eqs. (S39) to (S43), along with the inequality for case [C3], it follows that:

$$H_R(-\infty) > 0 \quad [\text{C3}], \tag{S58}$$

$$H_R(A_{nR}^{-1}(*)) < 0 \quad [\text{C3}], \tag{S59}$$

$$H_R(0) < 0 \quad [\text{C3}], \tag{S60}$$

$$H_R(A_{nR}^{-1}(0)) = 0 \quad [\text{C3}], \tag{S61}$$

$$H_R(C_a) = 0 \quad [\text{C3}], \tag{S62}$$

$$H_R(\infty) > 0 \quad [\text{C3}]. \tag{S63}$$

Since $H_R(H_R^{-1}(0)^+) = 0$, we find $H_R^{-1}(0)^+ = A_{nR}^{-1}(0) = C_a$. Thus, we have:

$$H_R^{-1}(0)^- < A_{nR}^{-1}(*) < 0 < C_a = A_{nR}^{-1}(0) = H_R^{-1}(0)^+ \quad [\text{C3}] \tag{S64}$$

In case [C4], we have $A_{nR}^{-1}(0) < A_{nR}^{-1}(*) < 0 \leq C_a$ (Table 3). From Eqs. (S39) to (S43), along

with the inequality for case [C4], it follows that:

$$H_R(-\infty) > 0 \quad [C4], \tag{S65}$$

$$H_R(A_{nR}^{-1}(0)) > 0 \quad [C4], \tag{S66}$$

$$H_R(A_{nR}^{-1}(*)) < 0 \quad [C4], \tag{S67}$$

$$H_R(0) < 0 \quad [C4], \tag{S68}$$

$$H_R(C_a) < 0 \quad [C4], \tag{S69}$$

$$H_R(\infty) > 0 \quad [C4]. \tag{S70}$$

Therefore, following the same reasoning, we derive the inequality relation:

$$A_{nR}^{-1}(0) < H_R^{-1}(0)^- < A_{nR}^{-1}(*) < 0 \leq C_a < H_R^{-1}(0)^+ \quad [C4] \tag{S71}$$

In case [C5], we have $A_{nR}^{-1}(*) < 0 \leq C_a$ (Table 3). From Eqs. (S39) to (S43), along with the inequality for case [C5], it follows that:

$$H_R(-\infty) > 0 \quad [C5], \tag{S72}$$

$$H_R(A_{nR}^{-1}(*)) < 0 \quad [C5], \tag{S73}$$

$$H_R(0) < 0 \quad [C5], \tag{S74}$$

$$H_R(C_a) < 0 \quad [C5], \tag{S75}$$

$$H_R(\infty) > 0 \quad [C5]. \tag{S76}$$

Thus, from the same discussion as above, we have the following inequality relation:

$$H_R^{-1}(0)^- < A_{nR}^{-1}(*) < 0 \leq C_a < H_R^{-1}(0)^+ \quad [C5] \tag{S77}$$

Table S7 summarizes the inequality relationships for $H_R(C_i)$ across cases [C1] to [C5].

Table S7: Inequalities for $H_R(C_i)$

Cases	Inequality
[C1]	$H_R^{-1}(0)^- < A_{nR}^{-1}(*) < 0 < A_{nR}^{-1}(0) < H_R^{-1}(0)^+ < C_a$
[C2]	$H_R^{-1}(0)^- < A_{nR}^{-1}(*) < 0 \leq C_a < H_R^{-1}(0)^+ < A_{nR}^{-1}(0)$
[C3]	$H_R^{-1}(0)^- < A_{nR}^{-1}(*) < 0 < C_a = A_{nR}^{-1}(0) = H_R^{-1}(0)^+$
[C4]	$A_{nR}^{-1}(0) < H_R^{-1}(0)^- < A_{nR}^{-1}(*) < 0 \leq C_a < H_R^{-1}(0)^+$
[C5]	$H_R^{-1}(0)^- < A_{nR}^{-1}(*) < 0 \leq C_a < H_R^{-1}(0)^+$

(d) Geometry of $H_R(C_i)$

Figure S1 shows the curve of $H_R(C_i)$ for case [C1]. The shapes of the curves for the other cases are similar to that of [C1], but the inequalities among points such as $H_R^{-1}(0)^{\pm}$, $A_{nR}^{-1}(*)$, $A_{nR}^{-1}(0)$, and C_a vary for each case. Tables S8 to S12 present the sign charts of $H_R(C_i)$ for cases [C1] to [C5].

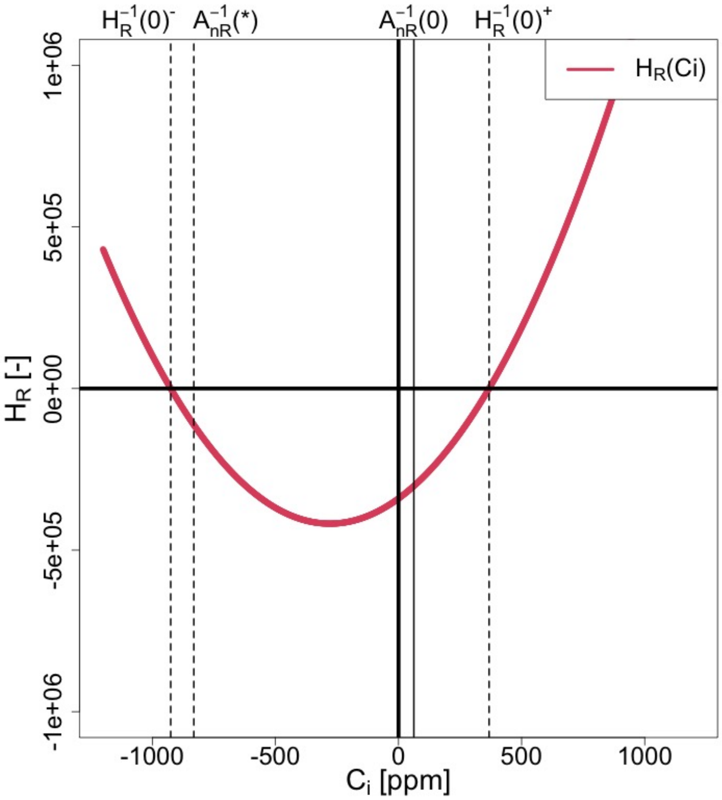

Figure S1: $H_R(C_i)$ [C1]

Table S8: Sign chart of $H_R(C_i)$ [C1]

C_i	$-\infty$		$H_R^{-1}(0)^-$		$A_{nR}^{-1}(*)$		0		$A_{nR}^{-1}(0)$		$H_R^{-1}(0)^+$		C_a		$+\infty$
H_R	$+\infty$	+	0	-	-	-	-	-	-	-	0	+	+	+	$+\infty$

Table S9: Sign chart of $H_R(C_i)$ [C2]

C_i	$-\infty$		$H_R^{-1}(0)^-$		$A_{nR}^{-1}(*)$		0		C_a		$H_R^{-1}(0)^+$		$A_{nR}^{-1}(0)$		$+\infty$
H_R	$+\infty$	+	0	-	-	-	-	-	-	-	0	+	+	+	$+\infty$

Table S10: Sign chart of $H_R(C_i)$ [C3]

C_i	$-\infty$		$H_R^{-1}(0)^-$		$A_{nR}^{-1}(*)$		0		$C_a = H_R^{-1}(0)^+ = A_{nR}^{-1}(0)$		$+\infty$
H_R	$+\infty$	+	0	-	-	-	-	-	0	+	$+\infty$

Table S11: Sign chart of $H_R(C_i)$ [C4]

C_i	$-\infty$		$A_{nR}^{-1}(0)$		$H_R^{-1}(0)^-$		$A_{nR}^{-1}(*)$		0		C_a		$H_R^{-1}(0)^+$		$+\infty$
H_R	$+\infty$	+	+	+	0	-	-	-	-	-	-	-	0	+	$+\infty$

Table S12: Sign chart of $H_R(C_i)$ [C5]

C_i	$-\infty$		$H_R^{-1}(0)^-$		$A_{nR}^{-1}(*)$		0		C_a		$H_R^{-1}(0)^+$		$+\infty$
H_R	$+\infty$	+	0	-	-	-	-	-	-	-	0	+	$+\infty$

SI 2.3 $H'_R(C_i)$

By differentiating $H_R(C_i)$ with respect to C_i under cases [C1] to [C5] (Eq. (S26)), we have:

$$H'_R(C_i) = 2C_i + \beta = \begin{cases} 2C_i - \left\{C_a + A_{nR}^{-1}(*) - \lambda_b \frac{A_{nR}(\pm\infty)}{g_b}\right\} & \text{[C1 to C4]} \\ 2C_i - \left\{C_a + A_{nR}^{-1}(*)\right\} & \text{[C5]} \end{cases} \tag{S78}$$

(a) $H_R'^{-1}(0)$

From Eq. (S78), we obtain

$$H_R'^{-1}(0) = -\frac{\beta}{2} = \begin{cases} \frac{\left\{C_a + A_{nR}^{-1}(*) - \lambda_b \frac{A_{nR}(\pm\infty)}{g_b}\right\}}{2} & \text{[C1 to C4]} \\ \frac{\left\{C_a + A_{nR}^{-1}(*)\right\}}{2} & \text{[C5]} \end{cases} \tag{S79}$$

$H_R'^{-1}(0)$ represents the value of C_i at which the slope of $H_R(C_i)$ is zero. Since $H_R'^{-1}(0)$ must lie between the two roots of $H_R(C_i) = 0$, it satisfies the following inequality:

$$H_R^{-1}(0)^- < H_R'^{-1}(0) < H_R^{-1}(0)^+ \tag{S80}$$

(b) Signs of $H'_R(C_i)$

Since $H_R(C_i)$ is a convex quadratic function of C_i, the sign of $H'_R(C_i)$ changes from negative to positive at $H_R'^{-1}(0)$. Hence, we have:

$$H'_R(H_R'^{-1}(0) < C_i) > 0 \tag{S81}$$

$$H'_R(C_i < H_R'^{-1}(0)) < 0 \tag{S82}$$

From Eqs. (S80) to (S82), we obtain:

$$H'_R(H_R^{-1}(0)^+ \leq C_i) > 0 \tag{S83}$$

$$H'_R(C_i \leq H_R^{-1}(0)^-) < 0 \tag{S84}$$

SI 2.4 $F_R(C_i)$

$F_R(C_i)$ under cases [C1] to [C5] is given by Eq. (S25):

$$F_R(C_i) = \begin{cases} -\frac{\lambda_s A_{nR}(\pm\infty)\{C_i - A_{nR}^{-1}(0)\}}{H_R(C_i)} & \text{[C1 to C4]} \\ -\frac{\lambda_s A_{nR}(0)}{H_R(C_i)} & \text{[C5]} \end{cases} \tag{S85}$$

(a) Singularities: $F_R^{-1}(*)$

Eq. (S85) shows that $F_R(C_i)$ has singularities at points C_i where $H_R(C_i) = 0$. Thus, we can express the singularities as:

$$F_R^{-1}(*) = H_R^{-1}(0)^{\pm} \tag{S86}$$

To differentiate between the two singularities, we denote them as:

$$F_R^{-1}(*)^+ = H_R^{-1}(0)^+ \tag{S87}$$

$$F_R^{-1}(*)^- = H_R^{-1}(0)^- \tag{S88}$$

The behavior of $F_R(C_i)$ in the neighborhood of $F_R^{-1}(*)^+$ is expressed as

$$F_R(F_R^{-1}(*)^+ \pm \epsilon) = F_R(H_R^{-1}(0)^+ \pm \epsilon) = \begin{cases} -\frac{\lambda_s A_{nR}(\pm\infty)\{H_R^{-1}(0)^+ - A_{nR}^{-1}(0) \pm \epsilon\}}{\pm\epsilon} & \text{[C1 to C4]} \\ -\frac{\lambda_s A_{nR}(0)}{\pm\epsilon} & \text{[C5]} \end{cases} \tag{S89}$$

In the case of [C1], $A_{nR}(\pm\infty) > 0$ (Eq. (S12) and Table 3) and $H_R^{-1}(0)^+ > A_{nR}^{-1}(0)$ (Eq. (S50)). Therefore, we obtain $F_R(F_R^{-1}(*)^+ \pm \epsilon) = \mp\infty$ for [C1]. Similarly, for cases [C2] to [C5], we have:

$$F_R(F_R^{-1}(*)^+ \pm \epsilon) = \begin{cases} \mp\infty & [\text{C1}] \\ \pm\infty & [\text{C2, C4, and C5}] \\ -\epsilon/\epsilon & [\text{C3}] \end{cases} \tag{S90}$$

Following similar reasoning, we find:

$$F_R(F_R^{-1}(*)^- \pm \epsilon) = \mp\infty \quad [\text{C1 to C5}] \tag{S91}$$

In case [C3], $F_R(F_R^{-1}(*)^+) = 0/0$ (Eq. (S90)), indicating that $F_R(C_i)$ at $F_R^{-1}(*)^+$ is indeterminate under [C3], as follows:

$$F_R(F_R^{-1}(0)^+) = \text{indeterminate} \quad [\text{C3}] \tag{S92}$$

(b) Roots: $F_R^{-1}(0)$

From Eqs. (S85), we have:

$$F_R^{-1}(0) = \begin{cases} A_{nR}^{-1}(0) & [\text{C1 to C4}] \\ \text{does not exist} & [\text{C5}] \end{cases} \tag{S93}$$

In case [C3], $C_a = H_R^{-1}(0)^+ = A_{nR}^{-1}(0)$ under [C3] (S64). Combining this with Eqs. (S87) and (S93), we have:

$$C_a = H_R^{-1}(0)^+ = A_{nR}^{-1}(0) = F_R^{-1}(*)^+ = F_R^{-1}(0) \quad [\text{C3}] \tag{S94}$$

(c) $F_R(\pm\infty)$ and $F_R(A_{nR}^{-1}(*))$

$$F_R(\pm\infty) = \begin{cases} -\frac{\lambda_s A_{nR}(\pm\infty)\{\pm\infty - A_{nR}^{-1}(0)\}}{H_R(\pm\infty)} & [\text{C1 to C4}] \\ -\frac{\lambda_s A_{nR}(0)}{H_R(\pm\infty)} & [\text{C5}] \end{cases} \tag{S95}$$

Noting that the denominator of $F_R(C_i)$ is a quadratic function of C_i (Eq. (S26)), while the numerator is linear for [C1 to C4] or constant for [C5], we find:

$$F_R(\pm\infty) = \begin{cases} \mp 0 & \text{[C1 to C3]} \\ \pm 0 & \text{[C4]} \\ +0 & \text{[C5]} \end{cases} \tag{S96}$$

By using Eqs. (S25) and (S40), $F_R(A_{nR}^{-1}(*))$ is given by

$$F_R(A_{nR}^{-1}(*)) = -\frac{\lambda_s g_b}{\lambda_b} \quad (< 0) \tag{S97}$$

(d) Geometry of $F_R(C_i)$

According to the distinct geometries of $A_{nR}(C_i)$ and $H_R(C_i)$ for cases [C1] to [C5] (Sections SI 1 and SI 2.2), $F_R(C_i)$ also exhibits distinct geometries for each case. To differentiate these, we denote them as $F_{R1}(C_i)$ to $F_{R5}(C_i)$ for cases [C1] to [C5], respectively. Figure 2 displays the curves of $F_{R1}(C_i)$ to $F_{R5}(C_i)$, and Tables S13 to S17 provide the sign charts of $F_{R1}(C_i)$ to $F_{R5}(C_i)$ as well as the signs of the numerator and denominator. In the figures and tables, we use the inequality relations between the points for cases [C1] to [C5] (Table S7), and two equations: $F_R^{-1}(*)^{\pm} = H_R^{-1}(0)^{\pm}$ (Eqs. (S87) and (S88)) and $F_R^{-1}(0) = A_{nR}^{-1}(0)$ for cases [C1] to [C4] (Eq. (S93)).

Table S13: Sign chart of $F_{R1}(C_i)$

C_i	$-\infty$		$F_R^{-1}(*)^-$		$A_{nR}^{-1}(*)$		0		$F_R^{-1}(0)$		$F_R^{-1}(*)^+$		C_a		$+\infty$
$-\lambda_s A_{nR}(\pm\infty)\{C_i - A_{nR}^{-1}(0)\}$	$+\infty$	+	+	+	+	+	+	+	0	-	-	-	-	-	$-\infty$
H_R	$+\infty$	+	0	-	-	-	-	-	-	-	0	+	+	+	$+\infty$
F_R	$+0$	+	$+\infty, -\infty$	-	-	-	-	-	0	+	$+\infty, -\infty$	-	-	-	-0

Table S14: Sign chart of $F_{R2}(C_i)$

C_i	$-\infty$		$F_R^{-1}(*)^-$		$A_{nR}^{-1}(*)$		0		C_a		$F_R^{-1}(*)^+$		$F_R^{-1}(0)$		$+\infty$
$-\lambda_s A_{nR}(\pm\infty)\{C_i - A_{nR}^{-1}(0)\}$	$+\infty$	+	+	+	+	+	+	+	+	+	+	+	0	-	$-\infty$
H_R	$+\infty$	+	0	-	-	-	-	-	-	-	0	+	+	+	$+\infty$
F_R	$+0$	+	$+\infty, -\infty$	-	-	-	-	-	-	-	$-\infty, +\infty$	+	0	-	-0

Table S15: Sign chart of $F_{R3}(C_i)$

C_i	$-\infty$		$F_R^{-1}(*)^-$		$A_{nR}^{-1}(*)$		0		$C_a = F_R^{-1}(*)^+ = F_R^{-1}(0)$		$+\infty$
$-\lambda_s A_{nR}(\pm\infty)\{C_i - A_{nR}^{-1}(0)\}$	$+\infty$	+	+	+	+	+	+	+	0	-	$-\infty$
H_R	$+\infty$	+	0	-	-	-	-	-	0	+	$+\infty$
F_R	$+0$	+	$+\infty, -\infty$	-	-	-	-	-	$-0/0$	-	-0

Table S16: Sign chart of $F_{R4}(C_i)$

C_i	$-\infty$		$F_R^{-1}(0)$		$F_R^{-1}(*)^-$		$A_{nR}^{-1}(*)$		0		C_a		$F_R^{-1}(*)^+$		$+\infty$
$-\lambda_s A_{nR}(\pm\infty)\{C_i - A_{nR}^{-1}(0)\}$	$-\infty$	-	0	+	+	+	+	+	+	+	+	+	+	+	$+\infty$
H_R	$+\infty$	+	+	+	0	-	-	-	-	-	-	-	0	+	$+\infty$
F_R	-0	-	0	+	$+\infty, -\infty$	-	-	-	-	-	-	-	$-\infty, +\infty$	+	$+0$

Table S17: Sign chart of $F_{R5}(C_i)$

C_i	$-\infty$		$F_R^{-1}(*)^-$		$A_{nR}^{-1}(*)$		0		C_a		$F_R^{-1}(*)^+$		$+\infty$
$-\lambda_s A_{nR}(0)$	+	+	+	+	+	+	+	+	+	+	+	+	+
H_R	$+\infty$	+	0	-	-	-	-	-	-	-	0	+	$+\infty$
F_R	$+0$	+	$+\infty, -\infty$	-	-	-	-	-	-	-	$-\infty, +\infty$	+	$+0$

(e) Possible Range of Appropriate Solutions for $F_R(C_i)$

Based on the geometries of $F_R(C_i)$ identified in the previous section, the possible range of appropriate solutions is determined by selecting the interval of C_i where $F_R(C_i) > 0$ and $C_i > 0$. Table S18 summarizes the possible ranges of appropriate solutions for $F_R(C_i)$. In Figure 2, the regions outside the possible ranges of appropriate solutions are shaded in gray.

Table S18: Possible range of appropriate solutions for $F_R(C_i)$

[C]	$F_R(C_i)$	Range
[C1]	$F_{R1}(C_i)$	$F_R^{-1}(0) < C_i < F_R^{-1}(*)^+$
[C2]	$F_{R2}(C_i)$	$F_R^{-1}(*)^+ < C_i < F_R^{-1}(0)$
[C3]	$F_{R3}(C_i)$	$C_i = C_a = F_R^{-1}(*)^+ = F_R^{-1}(0)$
[C4]	$F_{R4}(C_i)$	$F_R^{-1}(*)^+ < C_i$
[C5]	$F_{R5}(C_i)$	$F_R^{-1}(*)^+ < C_i$

SI 2.5 $F'_R(C_i)$

Differentiating $F_R(C_i)$ with respect to C_i for cases [C1] to [C5] (Eq. (S85)) gives the derivative of $F_R(C_i)$ as follows:

$$F'_R(C_i) = \begin{cases} -\frac{\lambda_s A_{nR}(\pm\infty)\tilde{H}_R(C_i)}{H_R^2(C_i)} & \text{[C1 to C4]} \\ \frac{\lambda_s A_{nR}(0)H'_R(C_i)}{H_R^2(C_i)} & \text{[C5]} \end{cases} \tag{S98}$$

where $\tilde{H}_R(C_i)$ is defined as:

$$\tilde{H}_R(C_i) = H_R(C_i) - \{C_i - A_{nR}^{-1}(0)\}H'_R(C_i). \quad \text{[C1 to C4]} \tag{S99}$$

SI 2.5.1 $\tilde{H}_R(C_i)$

Using Eqs. (S26) and (S78), $\tilde{H}_R(C_i)$ can be rewritten as follows:

$$\tilde{H}_R(C_i) = H_R(C_i) - \{C_i - A_{nR}^{-1}(0)\}H'_R(C_i) \tag{S100}$$

$$= -\{C_i - A_{nR}^{-1}(0)\}^2 + \{C_a - A_{nR}^{-1}(0)\}\{A_{nR}^{-1}(*) - A_{nR}^{-1}(0)\} \tag{S101}$$

Eq. (S101) shows that $\tilde{H}_R(C_i)$ is a concave quadratic function of C_i and attains its maximum at $C_i = A_{nR}^{-1}(0)$.

(a) $\tilde{H}_R(C_a)$

$$\tilde{H}_R(C_a) = -\{C_a - A_{nR}^{-1}(0)\}\{C_a - A_{nR}^{-1}(*)\} \tag{S102}$$

Considering the inequalities in Table S7, Eq. (S102) gives the following inequalities:

$$\tilde{H}_R(C_a) \begin{cases} < 0 & \text{[C1 and C4]} \\ > 0 & \text{[C2]} \\ = 0 & \text{[C3]} \end{cases} \tag{S103}$$

(b) $\tilde{H}_R(A_{nR}^{-1}(0))$

$$\tilde{H}_R(A_{nR}^{-1}(0)) = \{C_a - A_{nR}^{-1}(0)\}\{A_{nR}^{-1}(*) - A_{nR}^{-1}(0)\} \tag{S104}$$

By using the inequalities in Table S7, we obtain

$$\tilde{H}_R(A_{nR}^{-1}(0)) \begin{cases} > 0 & \text{[C2 and C4]} \\ < 0 & \text{[C1]} \\ = 0 & \text{[C3]} \end{cases} \tag{S105}$$

(c) $\tilde{H}_R(H_R^{-1}(0)^+)$

$$\tilde{H}_R(H_R^{-1}(0)^+) = -\{H_R^{-1}(0)^+ - A_{nR}^{-1}(0)\}H'_R(H_R^{-1}(0)^+) \tag{S106}$$

By using the inequalities in Table S7 and $H'_R(H_R^{-1}(0)^+) > 0$ (Eq. (S83)), we obtain:

$$\tilde{H}_R(H_R^{-1}(0)^+) \begin{cases} > 0 & \text{[C2]} \\ < 0 & \text{[C1 and C4]} \\ = 0 & \text{[C3]} \end{cases} \tag{S107}$$

(d) Sign of $\tilde{H}_R(C_i)$

We examine the sign of $\tilde{H}_R(C_i)$, focusing on the possible range of appropriate solutions (Table S18). Note that $\tilde{H}_R(C_i)$ is a concave quadratic function of C_i, which attains its maximum at $C_i = A_{nR}^{-1}(0)$ (Eq. S101).

In case [C1], since $\tilde{H}_R(A_{nR}^{-1}(0)) < 0$ (Eq. (S105)), we have:

$$\tilde{H}_R(C_i) < 0 \quad [\text{C1}]. \tag{S108}$$

For case [C2], given that $H_R^{-1}(0)^+ < C_i < A_{nR}^{-1}(0)$ (Table S7), $\tilde{H}_R(A_{nR}^{-1}(0)) > 0$ (Eq. (S105)), and $\tilde{H}_R(H_R^{-1}(0)^+) > 0$ (Eq. (S107)), it follows from Eq. (S101) that:

$$\tilde{H}_R(H_R^{-1}(0)^+ < C_i < A_{nR}^{-1}(0)) > 0 \quad [\text{C2}]. \tag{S109}$$

By combining Eq. (S109) with $F_R^{-1}(*)^+ = H_R^{-1}(0)^+$ (Eq. (S87)) and $F_R^{-1}(0) = A_{nR}^{-1}(0)$ (Eq. (S93)), we have:

$$\tilde{H}_R(F_R^{-1}(*)^+ < C_i < F_R^{-1}(0)) > 0 \quad [\text{C2}]. \tag{S110}$$

For case [C4], given that $A_{nR}^{-1}(0) < C_i < H_R^{-1}(0)^+$ (Table S7) and $\tilde{H}_R(H_R^{-1}(0)^+) < 0$ (Eq. (S107)), it follows from Eq. (S99) that:

$$\tilde{H}_R(H_R^{-1}(0)^+ < C_i) < 0 \quad [\text{C4}]. \tag{S111}$$

Similarly to case [C2], by combining Eq. (S111) with $F_R^{-1}(*)^+ = H_R^{-1}(0)^+$ (Eq. (S87)), we have:

$$\tilde{H}_R(F_R^{-1}(*)^+ < C_i) < 0 \quad [\text{C4}]. \tag{S112}$$

SI 2.5.2 Sign of $F_R'(C_i)$

We examine the sign of $F_R'(C_i)$, by using the results of the previous section.

For case [C1], since $A_{nR}(\pm\infty) > 0$ (Eq. (S12) and Table 3) and $\tilde{H}_R(C_i) < 0$ (Eq. S108), Eq. (S98) gives:

$$F_R'(F_R^{-1}(0) < C_i < F_R^{-1}(*)^+) > 0 \quad [\text{C1}]. \tag{S113}$$

For case [C2], given that $A_{nR}(\pm\infty) > 0$ (Eq. (S12) and Table 3) and $\tilde{H}_R(F_R^{-1}(*)^+ < C_i < F_R^{-1}(0)) > 0$ (Eq. S110), Eq. (S98) indicates:

$$F_R'(F_R^{-1}(*)^+ < C_i < F_R^{-1}(0)) < 0 \quad [C2]. \tag{S114}$$

For case [C4], given that $A_{nR}(\pm\infty) < 0$ (Eq. (S12) and Table 3) and $\tilde{H}_R(F_R^{-1}(0)^+ < C_i) < 0$ (Eq. S112), Eq. (S98) results in:

$$F_R'(F_R^{-1}(*)^+ < C_i) < 0 \quad [C4]. \tag{S115}$$

For case [C5], since $A_{nR}(0) < 0$ (Eq. S20) and $H_R'(H_R^{-1}(0)^+ \leq C_i) > 0$ (Eq. S83), Eq. (S98) leads to:

$$F_R'(H_R^{-1}(0)^+ < C_i) < 0 \quad [C5]. \tag{S116}$$

By combining Eq. (S116) with $F_R^{-1}(*)^+ = H_R^{-1}(0)^+$ (Eq. S87), we obtain:

$$F_R'(F_R^{-1}(*)^+ < C_i) < 0 \quad [C5]. \tag{S117}$$

SI 3 $F_{R0}(C_i)$ [$a = 0$, Rubisco/RuBP-limited]

In this chapter, we explore $F_{R0}(C_i)$, which is given by:

$$F_{R0}(C_i) = \frac{\lambda_s g_b R_L}{\{C_i - C_a\}g_b - \lambda_b R_L} \tag{S118}$$

(a) Derivative: $F'_{R0}(C_i)$

$$F'_{R0}(C_i) = -\frac{\lambda_s g_b^2 R_L}{\{\{C_i - C_a\}g_b - \lambda_b R_L\}^2} \quad (< 0) \tag{S119}$$

Thus, $F_{R0}(C_i)$ monotonically decreases with C_i, except at the singularity obtained in the next section.

(b) Singularity: $F^{-1}_{R0}(*)$

From Eq. (S118), $F^{-1}_{R0}(*)$ is given by:

$$F^{-1}_{R0}(*) = C_a + \frac{\lambda_b R_L}{g_b} \quad (> C_a) \tag{S120}$$

The behavior of $F_{R0}(C_i)$ in the neighborhood of $F^{-1}_{R0}(*)$ is given by:

$$F_{R0}(F^{-1}_{R0}(*) \pm \epsilon) = \frac{\lambda_s g_b R_L}{\pm\epsilon} = \pm\infty \tag{S121}$$

(c) $F_{R0}(\pm\infty)$ and $F_{R0}(C_a)$

$$F_{R0}(\pm\infty) = \pm 0 \tag{S122}$$

$$F_{R0}(C_a) = -\frac{\lambda_s g_b}{\lambda_b} \quad (< 0) \tag{S123}$$

(d) Geometry of $F_{R0}(C_i)$

Figure 2 and Table S19 show the curve and sign chart of $F_{R0}(C_i)$.

Table S19: Sign chart of $F_{R0}(C_i)$

	$-\infty$		C_a		$F_{R0}^{-1}(*)$		$+\infty$
$F_{R0}(C_i)$	-0	-	-	-	$-\infty, +\infty$	+	+0

(e) Possible range of appropriate solutions for $F_{R0}(C_i)$

Based on the geometries of $F_{R0}(C_i)$ identified in the previous section, the possible range of appropriate solutions can be determined by selecting the interval of C_i where $F_{R0}(C_i) > 0$ and $C_i > 0$. Table S20 summarizes the possible range of appropriate solutions for $F_{R0}(C_i)$. In Figure 2, the regions outside the possible ranges of appropriate solutions are shaded in gray.

Table S20: Possible range of appropriate solutions for $F_{R0}(C_i)$

$F_{R0}(C_i)$	Range
$F_{R0}(C_i)$	$F_{R0}^{-1}(*) < C_i$

SI 4 $F_P(C_i)$ [TPU-limited]

In this chapter, we investigate the geometry of $F_P(C_i)$, which is given by:

$$F_P(C_i) = -\frac{\lambda_s g_b A_{nP}}{\{C_i - C_a\}g_b + \lambda_b A_{nP}} = -\frac{\lambda_s g_b A_{nP}}{H_P(C_i)} \tag{S124}$$

where $H_P(C_i)$ is given by:

$$H_P(C_i) = \{C_i - C_a\}g_b + \lambda_b A_{nP} \tag{S125}$$

SI 4.1 $H_P(C_i)$

(a) Root: $H_P^{-1}(0)$

Setting $H_P(C_i) = 0$, we obtain:

$$H_P^{-1}(0) = C_a - \frac{\lambda_b A_{nP}}{g_b} \quad (< C_a) \tag{S126}$$

Therefore, we have:

$$H_P(C_i < H_P^{-1}(0)) < 0 \tag{S127}$$

$$H_P(H_P^{-1}(0) < C_i) > 0 \tag{S128}$$

In the case of TPU-limited conditions, from Eqs. (7), (9), and (13), we find:

$$C_s = C_a - \frac{\lambda_b A_{nP}}{g_b} = H_P^{-1}(0) \tag{S129}$$

Since $C_s < 0$ is physically impossible, we require $C_s > 0$. Thus, we have:

$$C_a - \frac{\lambda_b A_{nP}}{g_b} > 0 \iff \frac{\lambda_b A_{nP}}{g_b} < C_a \tag{S130}$$

Eq. (S130) provides the lower limit of C_a. Using Eqs. (S126), (S129), and (S130), we obtain:

$$0 < H_P^{-1}(0) < C_a \tag{S131}$$

(b) $H_P(0)$

By substituting $C_i = 0$ into Eq. (S125), we have:

$$H_P(0) = -g_b C_a + \lambda_b A_{nP} \tag{S132}$$

Using Eq. (S130), we find:

$$H_P(0) < 0 \tag{S133}$$

(c) $H'_P(C_i)$

Differentiating $H_P(C_i)$ (Eq. (S125)) with respect to C_i, we obtain:

$$H'_P(C_i) = g_b \tag{S134}$$

SI 4.2 $F_P(C_i)$

(a) Derivative: $F'_P(C_i)$

$$F'_P(C_i) = \frac{\lambda_s g_b^2 A_{nP}}{H_P^2(C_i)} \quad (> 0) \tag{S135}$$

Thus, $F_P(C_i)$ monotonically increases with C_i, except at the singularity calculated in the next section.

(b) Singularity: $F_P^{-1}(*)$

From Eq. (S124), $F_P^{-1}(*)$ is given by:

$$F_P^{-1}(*) = H_P^{-1}(0) \tag{S136}$$

(c) $F_P(\pm\infty)$, $F_P(0)$, and $F_P(C_a)$

$$F_P(\pm\infty) = \mp\infty \tag{S137}$$

$$F_P(0) = -\frac{\lambda_s g_b A_{nP}}{H_P(0)} \quad (>0) \tag{S138}$$

$$F_P(C_a) = -\frac{\lambda_s g_b}{\lambda_b} \quad (<0) \tag{S139}$$

(d) Geometry of $F_P(C_i)$

Figure 2 and Table S21 show the curve and sign chart of $F_P(C_i)$.

Table S21: Geometry of $F_P(C_i)$

C_i	$-\infty$		0		$F_P^{-1}(*)$		C_a		$+\infty$
$F_P(C_i)$	$+0$	+	+	+	$+\infty, -\infty$	-	-	-	-0

(e) Possible range of appropriate solutions for $F_P(C_i)$

Based on the geometries of $F_P(C_i)$ identified in the previous section, the possible range of appropriate solutions can be determined by selecting the interval of C_i where $F_P(C_i) > 0$ and $C_i > 0$. Table S22 summarizes the possible range of appropriate solutions for $F_P(C_i)$. In Figure 2, the regions outside the possible ranges of appropriate solutions are shaded in gray.

Table S22: Possible range of appropriate solutions for $F_P(C_i)$

$F_P(C_i)$	Range
$F_P(C_i)$	$0 < C_i < F_P^{-1}(*)$

SI 5 $Z_R(C_i)$ [C1, $e_a < e_i$]

We analyze the geometry of $Z_R(C_i)$, which represents $Z(C_i)$ under case [C1] and $e_a < e_i$ (Table 5). It is defined as:

$$\begin{aligned} Z_R(C_i) &= F_{R1}(C_i) - G_R(C_i) \\ &= F_R(C_i) - G_R(C_i) \\ &= \frac{-\lambda_s A_{nR}(\pm\infty)\{C_i - A_{nR}^{-1}(0)\}N_R(C_i)}{H_R(C_i)K_R(C_i)}, \end{aligned} \tag{S140}$$

where $N_R(C_i)$ is expressed as:

$$N_R(C_i) = C_i\{C_i - A_{nR}^{-1}(*)\} + \tilde{g}_{slp}\sqrt{\frac{I_R(C_i)}{H_R(C_i)}}H_R(C_i), \tag{S141}$$

with $I_R(C_i)$ and $K_R(C_i)$ defined as:

$$I_R(C_i) = C_i^2 - \left\{C_a + A_{nR}^{-1}(*) - (\lambda_b - \lambda_s)\frac{A_{nR}(\pm\infty)}{g_b}\right\}C_i + C_a A_{nR}^{-1}(*) - (\lambda_b - \lambda_s)\frac{A_{nR}(\pm\infty)}{g_b}A_{nR}^{-1}(0), \tag{S142}$$

$$K_R(C_i) = \left\{C_a - \frac{\lambda_b A_{nR}(\pm\infty)}{g_b}\right\}C_i - H_R^{-1}(0)^+ H_R^{-1}(0)^-. \tag{S143}$$

The function $N_R(C_i)$ is real for C_i satisfying:

$$H_R(C_i)I_R(C_i) > 0. \tag{S144}$$

Hence, Eq. (S144) is assumed throughout this section. Additionally, [C1] is assumed for all analyses in this chapter, since case [C1] is a condition for $Z_R(C_i)$ (Table 6).

SI 5.1 $I_R(C_i)$

(a) $I_R(A_{nR}^{-1}(0))$

$I_R(A_{nR}^{-1}(0))$ is given by:

$$I_R(A_{nR}^{-1}(0)) = \{A_{nR}^{-1}(0) - C_a\}\{A_{nR}^{-1}(0) - A_{nR}^{-1}(*)\} = H_R(A_{nR}^{-1}(0)) \quad \text{[C1 to C4]} \tag{S145}$$

Since $A_{nR}^{-1}(*) < 0 < A_{nR}^{-1}(0) < C_a$ under [C1] (Table 3), we have:

$$I_R(A_{nR}^{-1}(0)) < 0 \quad \text{[C1]} \tag{S146}$$

Thus, $I_R(C_i)$ has a negative value at $C_i = A_{nR}^{-1}(0)$.

(b) Root: $I_R^{-1}(0)$

Setting $I_R(C_i) = 0$ yields a quadratic polynomial equation with respect to C_i in the form $\alpha C_i^2 + \tilde{\beta} C_i + \tilde{\gamma} = 0$, where the coefficients are given by:

$$\tilde{\beta} = -\left\{C_a + A_{nR}^{-1}(*) - (\lambda_b - \lambda_s)\frac{A_{nR}(\pm\infty)}{g_b}\right\} \quad \text{(S147)}$$

$$\tilde{\gamma} = C_a A_{nR}^{-1}(*) - (\lambda_b - \lambda_s)\frac{A_{nR}(\pm\infty)}{g_b} A_{nR}^{-1}(0) \quad \text{(S148)}$$

Note that $\alpha = 1$ (Eq. (S27)). Eqs. (S142) and (S146) show that $I_R(C_i)$ is a convex quadratic function of C_i and has a negative value at $C_i = A_{nR}^{-1}(0)$. Therefore, $I_R(C_i) = 0$ has two real solutions, expressed as:

$$I_R^{-1}(0)^L = \frac{-\tilde{\beta} + \sqrt{\tilde{\beta}^2 - 4\alpha\tilde{\gamma}}}{2\alpha} \quad \text{(S149)}$$

$$I_R^{-1}(0)^S = \frac{-\tilde{\beta} - \sqrt{\tilde{\beta}^2 - 4\alpha\tilde{\gamma}}}{2\alpha} \quad \text{(S150)}$$

The two roots satisfy the following inequality:

$$I_R^{-1}(0)^S < A_{nR}^{-1}(0) < I_R^{-1}(0)^L \quad \text{(S151)}$$

Since $A_{nR}^{-1}(0) > 0$ under [C1] (Table 3), we have:

$$0 < I_R^{-1}(0)^L \quad \text{[C1]} \quad \text{(S152)}$$

Using $I_R^{-1}(0)^S$ and $I_R^{-1}(0)^L$, $I_R(C_i)$ can be expressed as:

$$I_R(C_i) = \{C_i - I_R^{-1}(0)^L\}\{C_i - I_R^{-1}(0)^S\} \quad \text{(S153)}$$

From the relationship between solutions and coefficients for quadratic equations, we have:

$$I_R^{-1}(0)^L + I_R^{-1}(0)^S = -\tilde{\beta} \quad \text{(S154)}$$

$$I_R^{-1}(0)^L I_R^{-1}(0)^S = \tilde{\gamma} \quad \text{(S155)}$$

Note that $\alpha = 1$ (Eq. (S27)) was considered in Eqs. (S154) and (S155). From Eqs. (S152) and (S155), the sign of $I_R^{-1}(0)^S$ under [C1] depends on the sign of $\tilde{\gamma}$ as follows:

$$I_R^{-1}(0)^S \begin{cases} > 0 & [\text{C1}, \tilde{\gamma} > 0] \\ < 0 & [\text{C1}, \tilde{\gamma} < 0] \\ = 0 & [\text{C1}, \tilde{\gamma} = 0] \end{cases} \tag{S156}$$

Eq. (S156) shows that the signs of $I_R^{-1}(0)^S$ are the same as those of $\tilde{\gamma}$ under [C1], as follows:

$$\text{sgn}(I_R^{-1}(0)^S) = \text{sgn}(\tilde{\gamma}) \quad [\text{C1}] \tag{S157}$$

Considering that $I_R(C_i)$ is convex, we have:

$$I_R(C_i < I_R^{-1}(0)^S) > 0 \tag{S158}$$

$$I_R(I_R^{-1}(0)^S < C_i < I_R^{-1}(0)^L) < 0 \tag{S159}$$

$$I_R(I_R^{-1}(0)^L < C_i) > 0 \tag{S160}$$

(c) $I'_R(C_i)$

By differentiating $I_R(C_i)$ with respect to C_i, we have:

$$I'_R(C_i) = 2C_i - \left\{ C_a + A_{nR}^{-1}(*) - (\lambda_b - \lambda_s) \frac{A_{nR}(\pm\infty)}{g_b} \right\} \tag{S161}$$

Since $I_R(C_i)$ is a convex quadratic function of C_i (Eq. (S142)) and $I_R(C_i) = 0$ has two real solutions (Eqs. (S149) and (S150)), we have the following inequalities:

$$I'_R(I_R^{-1}(0)^S) \;<\; 0 \tag{S162}$$

$$I'_R(I_R^{-1}(0)^L) \;>\; 0 \tag{S163}$$

(d) $I_R(A_{nR}^{-1}(*))$, $I_R(0)$, $I_R(C_a)$, and $I_R(\pm\infty)$

$$
\begin{aligned}
I_R(A_{nR}^{-1}(*)) &= (\lambda_b - \lambda_s)\,\frac{A_{nR}(\pm\infty)}{g_b}\{A_{nR}^{-1}(*) - A_{nR}^{-1}(0)\} && \text{(S164)}\\
I_R(0) &= \tilde{\gamma} = I_R^{-1}(0)^S I_R^{-1}(0)^L && \text{(S165)}\\
I_R(C_a) &= (\lambda_b - \lambda_s)\,\frac{A_{nR}(\pm\infty)}{g_b}\{C_a - A_{nR}^{-1}(0)\} && \text{(S166)}\\
I_R(\pm\infty) &= +\infty \quad (> 0) && \text{(S167)}
\end{aligned}
$$

Since $A_{nR}(\pm\infty) > 0$ and $A_{nR}^{-1}(*) < 0 < A_{nR}^{-1}(0) < C_a$ under [C1] (Table 3), we have:

$$
\begin{aligned}
I_R(A_{nR}^{-1}(*)) &> 0 \quad [\text{C1}] && \text{(S168)}\\
I_R(C_a) &< 0 \quad [\text{C1}] && \text{(S169)}
\end{aligned}
$$

(e) Inequality relations between $I_R^{-1}(0)$ and other points

In case [C1], $H_R^{-1}(0)^- < A_{nR}^{-1}(*) < 0 < A_{nR}^{-1}(0) < H_R^{-1}(0)^+ < C_a$ (Table S7). $I_R(A_{nR}^{-1}(*)) > 0$ (Eq. (S168)) and $I_R(A_{nR}^{-1}(0)) < 0$ (Eq. (S146)), indicating opposite signs at these two points. Similarly, $I_R(C_a) < 0$ (Eq. (S169)) and $I_R(+\infty) > 0$ (Eq. (S167)), indicating opposite signs at these points. Considering the inequality between two solutions (Eq. (S151)) and the signs of $I_R^{-1}(0)^S$ (Eq. (S156)), we have the following possible inequalities:

$$H_R^{-1}(0)^- < A_{nR}^{-1}(*) < I_R^{-1}(0)^S < 0 < A_{nR}^{-1}(0) < H_R^{-1}(0)^+ < C_a < I_R^{-1}(0)^L \quad [\text{C1}, \tilde{\gamma} < 0] \tag{S170}$$

$$H_R^{-1}(0)^- < A_{nR}^{-1}(*) < I_R^{-1}(0)^S = 0 < A_{nR}^{-1}(0) < H_R^{-1}(0)^+ < C_a < I_R^{-1}(0)^L \quad [\text{C1}, \tilde{\gamma} = 0] \tag{S171}$$

$$H_R^{-1}(0)^- < A_{nR}^{-1}(*) < 0 < I_R^{-1}(0)^S < A_{nR}^{-1}(0) < H_R^{-1}(0)^+ < C_a < I_R^{-1}(0)^L \quad [\text{C1}, \tilde{\gamma} > 0] \tag{S172}$$

(f) Geometry of $I_R(C_i)$

Figure S2 shows the curve of $I_R(C_i)$ under [C1, $\tilde{\gamma} < 0$]. The curves of $I_R(C_i)$ for other cases are the same as for [C1, $\tilde{\gamma} < 0$], except for the inequality relationships between points. Tables S23 to S25 show the sign charts of $I_R(C_i)$ for each case.

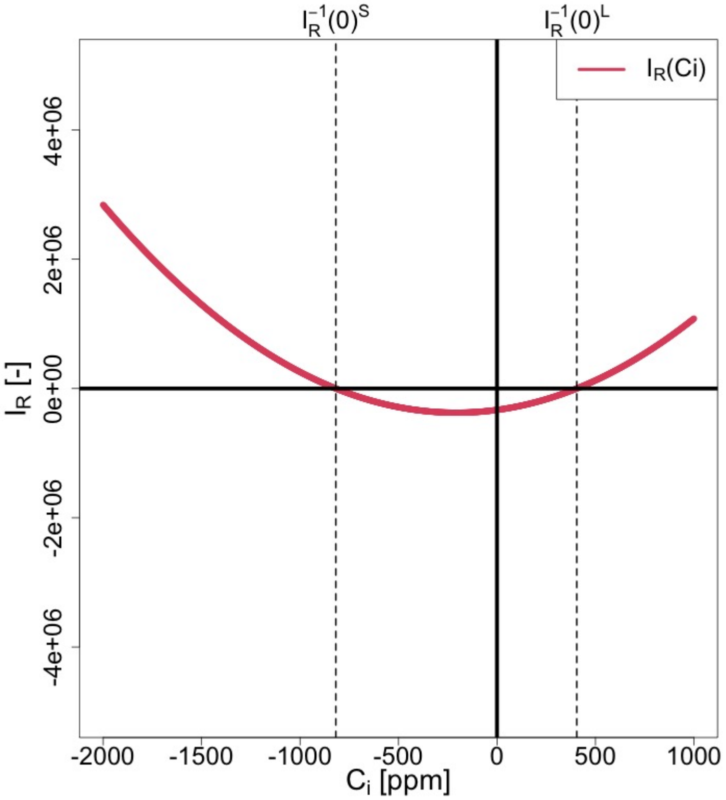

Figure S2: $I_R(C_i)$ [C1, $\tilde{\gamma} < 0$]

Table S23: Sign Chart of $I_R(C_i)$ under [C1, $\tilde{\gamma} < 0$]

C_i	$-\infty$		$H_R^{-1}(0)^-$		$A_{nR}^{-1}(*)$		$I_R^{-1}(0)^S$		0		$A_{nR}^{-1}(0)$		$H_R^{-1}(0)^+$		C_a		$I_R^{-1}(0)^L$		$+\infty$
I_R	$+\infty$	+	+	+	+	+	0	-	-	-	-	-	-	-	-	-	0	+	$+\infty$

Table S24: Sign Chart of $I_R(C_i)$ under [C1, $\tilde{\gamma} > 0$]

C_i	$-\infty$		$H_R^{-1}(0)^-$		$A_{nR}^{-1}(*)$		0		$I_R^{-1}(0)^S$		$A_{nR}^{-1}(0)$		$H_R^{-1}(0)^+$		C_a		$I_R^{-1}(0)^L$		$+\infty$
I_R	$+\infty$	+	+	+	+	+	+	+	0	-	-	-	-	-	-	-	0	+	$+\infty$

Table S25: Sign Chart of $I_R(C_i)$ under [C1, $\tilde{\gamma} = 0$]

C_i	$-\infty$		$H_R^{-1}(0)^-$		$A_{nR}^{-1}(*)$		$I_R^{-1}(0)^S = 0$		$A_{nR}^{-1}(0)$		$H_R^{-1}(0)^+$		C_a		$I_R^{-1}(0)^L$		$+\infty$
I_R	$+\infty$	+	+	+	+	+	0	-	-	-	-	-	-	-	0	+	$+\infty$

SI 5.2 $K_R(C_i)$

We examine the function $K_R(C_i)$, which is given from Eq. (S143) by:

$$K_R(C_i) = \phi C_i - H_R^{-1}(0)^+ H_R^{-1}(0)^- \tag{S173}$$

where ϕ is defined as

$$\phi = C_a - \frac{\lambda_b A_{nR}(\pm\infty)}{g_b} \tag{S174}$$

(a) Derivative: $K'_R(C_i)$

$$K'_R(C_i) = \phi \tag{S175}$$

Thus $K_R(C_i)$ is a linear function of C_i with a slope of ϕ.

(b) Root: $K_R^{-1}(0)$

If $\phi \neq 0$, $K_R(C_i) = 0$ yields:

$$K_R^{-1}(0) = \frac{H_R^{-1}(0)^+ H_R^{-1}(0)^-}{\phi} \quad [\phi \neq 0] \tag{S176}$$

Given $H_R^{-1}(0)^+ H_R^{-1}(0)^- < 0$ (Eq. (S35)), we obtain:

$$K_R^{-1}(0) \begin{cases} < 0 & [\phi > 0] \\ > 0 & [\phi < 0] \\ \text{not defined} & [\phi = 0] \end{cases} \tag{S177}$$

Since $K_R(C_i)$ is a linear function with slope ϕ, we have:

$$K_R(K_R^{-1}(0) < C_i) > 0 \quad [\phi > 0] \tag{S178}$$

$$K_R(C_i < K_R^{-1}(0)) > 0 \quad [\phi < 0] \tag{S179}$$

$$K_R(C_i) > 0 \quad [\phi = 0] \tag{S180}$$

(c) Inequality Relation between $K_R^{-1}(0)$, $H_R^{-1}(0)^+$, and $H_R^{-1}(0)^-$

If $\phi \neq 0$, then $H_R(K_R^{-1}(0))$ is given by:

$$H_R(K_R^{-1}(0)) = \left\{\frac{H_R^{-1}(0)^{+}H_R^{-1}(0)^{-}}{\phi^2}\right\}\left\{\lambda_b\frac{A_{nR}(\pm\infty)}{g_b}\right\}\{A_{nR}^{-1}(*) - A_{nR}^{-1}(0)\} \quad [\phi \neq 0] \quad \text{(S181)}$$

Given that $H_R^{-1}(0)^{+}H_R^{-1}(0)^{-} < 0$ (Eq. (S35)), along with $A_{nR}(\pm\infty) > 0$ and $A_{nR}^{-1}(*) < A_{nR}^{-1}(0)$ under [C1] (Table 3), we obtain:

$$H_R(K_R^{-1}(0)) > 0 \quad [\text{C1}, \phi \neq 0] \qquad \text{(S182)}$$

Since $H_R(C_i)$ is a convex quadratic function with two real roots $H_R^{-1}(0)^{+}$ and $H_R^{-1}(0)^{-}$, $H_R(K_R^{-1}(0)) > 0$ (Eq. (S182)) indicates that $K_R^{-1}(0)$ is located outside the region of $H_R^{-1}(0)^{-} < C_i < H_R^{-1}(0)^{+}$. Combining this with $H_R^{-1}(0)^{-} < 0 < A_{nR}^{-1}(0) < H_R^{-1}(0)^{+}$ under [C1] (Eq. (S50)) and the sings of $K_R^{-1}(0)$ (Eq. (S177)), we obtain the following inequalities:

$$K_R^{-1}(0) < H_R^{-1}(0)^{-} < 0 < A_{nR}^{-1}(0) < H_R^{-1}(0)^{+} \quad [\text{C1}, \phi > 0] \qquad \text{(S183)}$$

$$H_R^{-1}(0)^{-} < 0 < A_{nR}^{-1}(0) < H_R^{-1}(0)^{+} < K_R^{-1}(0) \quad [\text{C1}, \phi < 0] \qquad \text{(S184)}$$

By combining Eqs. (S183) and (S184) with Eqs. (S178) to (S180), we obtain:

$$K_R(A_{nR}^{-1}(0) < C_i < H_R^{-1}(0)^{+}) > 0 \quad [\text{C1}] \qquad \text{(S185)}$$

(d) $K_R(A_{nR}^{-1}(0))$

If $\phi \neq 0$, we have:

$$K_R(A_{nR}^{-1}(0)) = C_a(A_{nR}^{-1}(0) - A_{nR}^{-1}(*)) \quad [\phi \neq 0] \qquad \text{(S186)}$$

Since $A_{nR}^{-1}(*) < A_{nR}^{-1}(0)$ under [C1] (Table 3), it follows that:

$$K_R(A_{nR}^{-1}(0)) > 0 \quad [\text{C1}, \phi \neq 0] \qquad \text{(S187)}$$

By combining Eq. (S187) with Eq. (S180), we obtain:

$$K_R(A_{nR}^{-1}(0)) > 0 \quad [\text{C1}] \qquad \text{(S188)}$$

(e) Geometry of $K_R(C_i)$

Figures S3 to S5 and Tables S26 to S28 illustrate the curves and sign charts of $K_R(C_i)$.

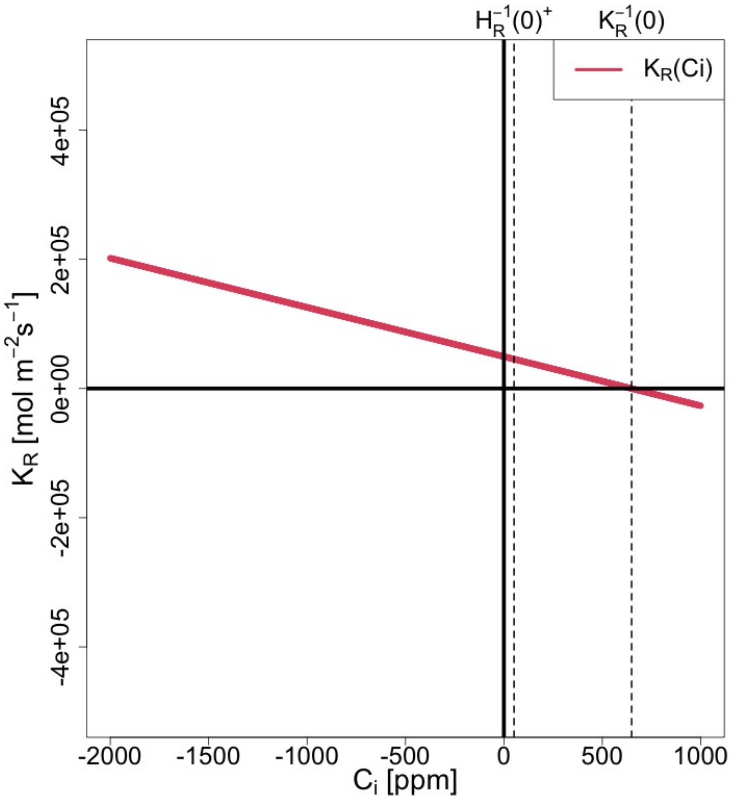

Figure S3: $K_R(C_i)$ for [C1, $\phi < 0$]

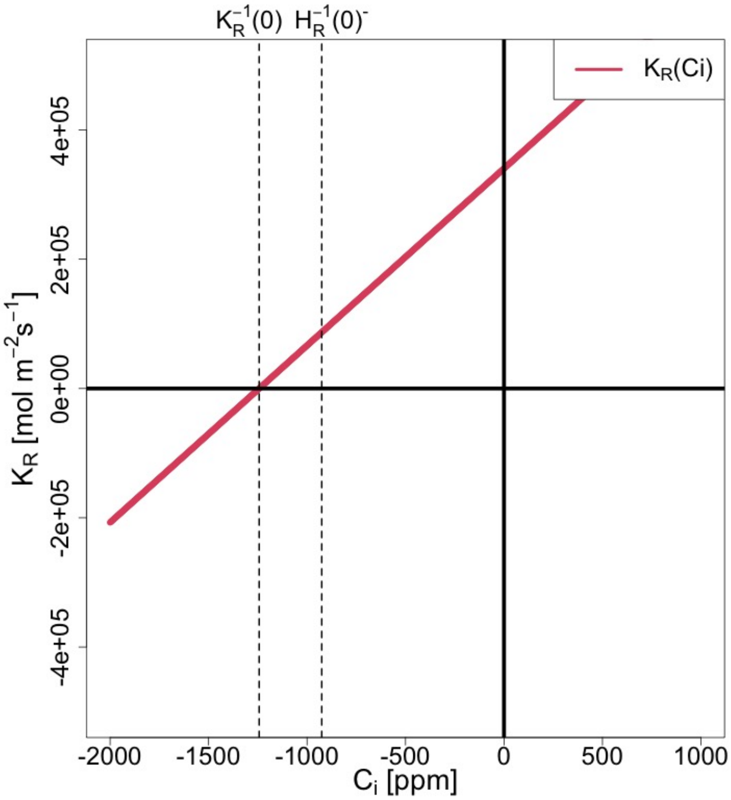

Figure S4: $K_R(C_i)$ for [C1, $\phi > 0$]

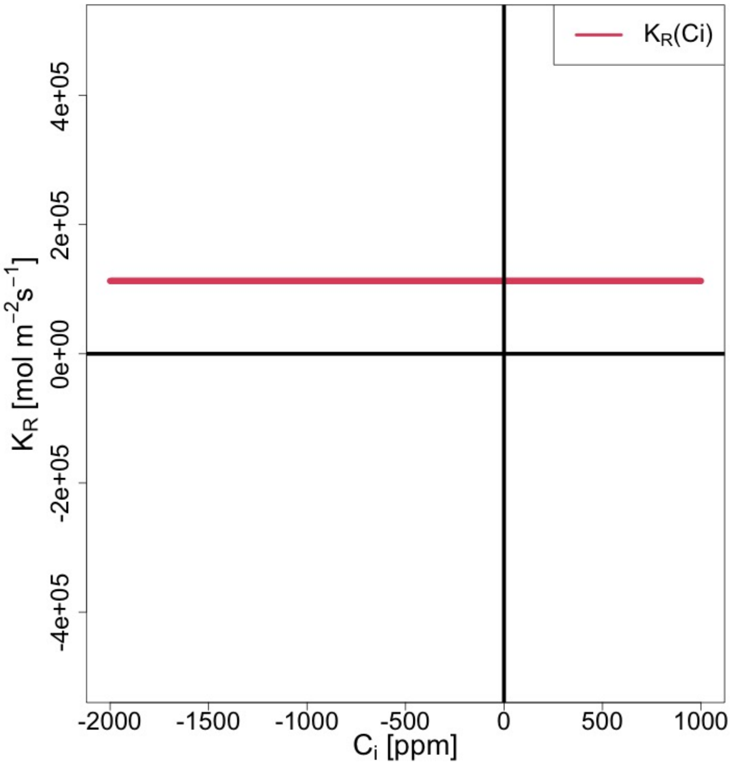

Figure S5: $K_R(C_i)$ for [C1, $\phi = 0$]

Table S26: Sign Chart of $K_R(C_i)$ [C1, $\phi < 0$]

C_i	$-\infty$		$H_R^{-1}(0)^-$		0		$H_R^{-1}(0)^+$		$K_R^{-1}(0)$		$+\infty$
K_R	$+\infty$	+	+	+	+	+	+	+	0	-	$-\infty$

Table S27: Sign Chart of $K_R(C_i)$ [C1, $\phi > 0$]

C_i	$-\infty$		$K_R^{-1}(0)$		$H_R^{-1}(0)^-$		0		$H_R^{-1}(0)^+$		$+\infty$
K_R	$-\infty$	-	0	+	+	+	+	+	+	+	$+\infty$

Table S28: Sign Chart of $K_R(C_i)$ [C1, $\phi = 0$]

C_i	$-\infty$		$H_R^{-1}(0)^-$		0		$H_R^{-1}(0)^+$		$+\infty$
K_R	+	+	+	+	+	+	+	+	+

SI 5.3 $N_R(C_i) = 0$

We explore the roots of $Z_R(C_i) = 0$ to examine the geometry of $Z_R(C_i)$. From Eq. (S140), the roots of $Z_R(C_i) = 0$ are $A_{nR}^{-1}(0)$ and those of $N_R(C_i) = 0$. Thus, we examine $N_R(C_i) = 0$. Note that [C1] is assumed for $N_R(C_i)$ since it is assumed for $Z_R(C_i)$.

$N_R(C_i)$ can be expressed as the difference between two functions, $L_R(C_i)$ and $M_R(C_i)$, as follows:

$$N_R(C_i) = L_R(C_i) - M_R(C_i) \tag{S189}$$

where $L_R(C_i)$ and $M_R(C_i)$ are given by

$$L_R(C_i) = C_i\{C_i - A_{nR}^{-1}(*)\} \tag{S190}$$

$$M_R(C_i) = -\tilde{g}_{slp}\sqrt{\frac{I_R(C_i)}{H_R(C_i)}}H_R(C_i) \tag{S191}$$

The roots of $N_R(C_i) = 0$, $N_R^{-1}(0)$, can be found as the values of C_i that satisfy $L_R(C_i) = M_R(C_i)$, which indicates the points of intersection between $L_R(C_i)$ and $M_R(C_i)$.

SI 5.3.1 $L_R(C_i)$

Eq. (S190) shows that $L_R(C_i)$ is a convex quadratic function of C_i.

(a) Roots: $L_R^{-1}(0)$

From Eq. (S190), $L_R^{-1}(0)$ is given by:

$$L_R^{-1}(0) = 0 \text{ and } A_{nR}^{-1}(*) \tag{S192}$$

(b) Geometry of $L_R(C_i)$

Figure S6 and Table S29 show the curve and sign chart of $L_R(C_i)$.

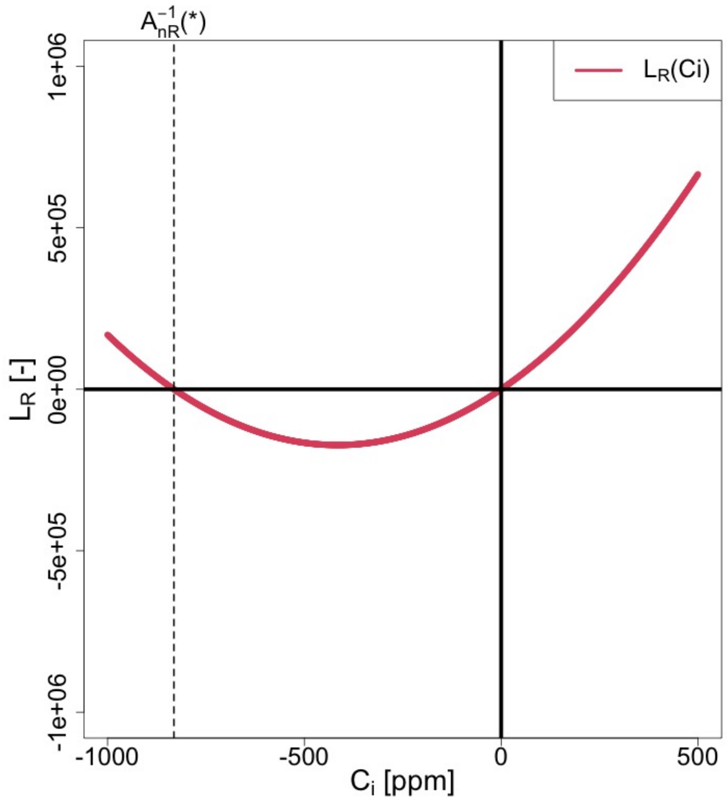

Figure S6: $L_R(C_i)$

Table S29: Sign chart of $L_R(C_i)$

C_i	$-\infty$		$A_{nR}^{-1}(*)$		0		$+\infty$
L_R	$+\infty$	+	0	-	0	+	$+\infty$

SI 5.3.2 $M_R(C_i)$

(a) Roots: $M_R^{-1}(0)$

From Eq. (S191), $M_R^{-1}(0)$ is given by:

$$M_R^{-1}(0) = H_R^{-1}(0)^+, H_R^{-1}(0)^-, I_R^{-1}(0)^L, \text{ and } I_R^{-1}(0)^S \tag{S193}$$

(b) Geometry of $M_R(C_i)$

Since there are three distinct inequalities for $I_R(C_i)$ under [C1], depending on the sign of $\tilde{\gamma}$, there are also three distinct inequalities for $M_R(C_i)$. Figure S7 illustrates the curve of $M_R(C_i)$ under the condition [C1, $\tilde{\gamma} < 0$]. Notably, the curves of $M_R(C_i)$ under [C1, $\tilde{\gamma} > 0$] and [C1, $\tilde{\gamma} = 0$] are identical to that under [C1, $\tilde{\gamma} < 0$]. Table S30 summarizes the sign chart of $M_R(C_i)$, focusing on the possible range of appropriate solutions under [C1] (see Table 4). Note that $A_{nR}^{-1}(0) = F_R^{-1}(0)$ (Eq. S93) and $H_R^{-1}(0)^+ = F_R^{-1}(*)^+$ (Eq. (S87)) are used in Table S30.

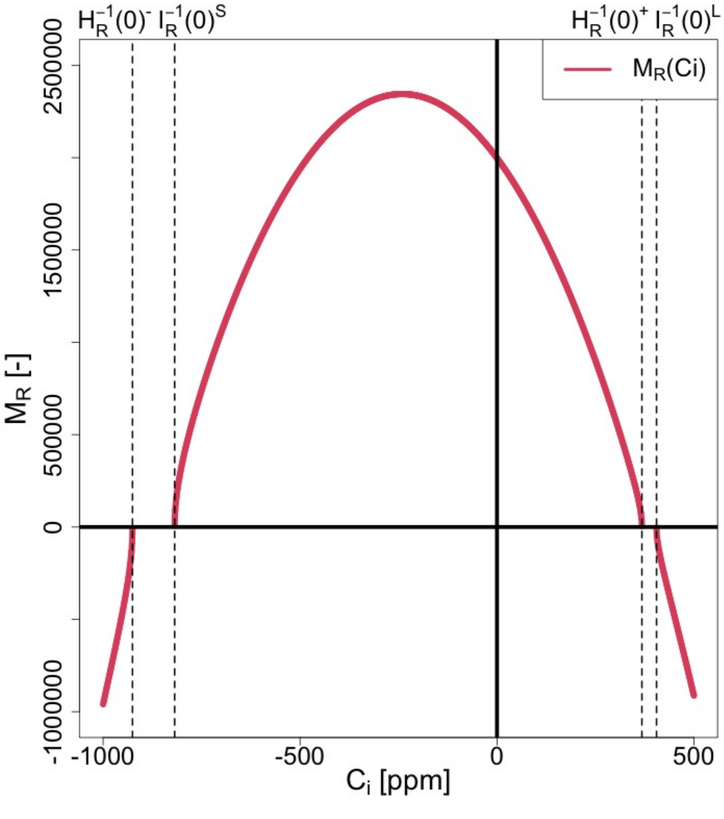

Figure S7: $M_R(C_i)$ [C1, $\tilde{\gamma} < 0$]

Table S30: Sign chart of $M_R(C_i)$ [C1]

C_i	$A_{nR}^{-1}(0)$		$H_R^{-1}(0)^+$
H_R	-	-	0
I_R	-	-	-
$\sqrt{\frac{I_R}{H_R}}$	+	+	$+\infty$
M_R	+	+	0

SI 5.3.3 Sign of $L_R(C_i)$ and $M_R(C_i)$

We focus on the interval $A_{nR}^{-1}(0) \leq C_i \leq H_{nR}^{-1}(0)^+$, which includes the open interval $A_{nR}^{-1}(0) < C_i < H_{nR}^{-1}(0)^+$ corresponding to the possible range of solutions under [C1], $F_R^{-1}(0) < C_i < F_R^{-1}(*)^+$ in Table 4 (Eqs. (S93) and (S87)). From Tables S29 and S30, it follows that both $L_R(C_i)$ and $M_R(C_i)$ are non-negative within this range:

$$L_R\left(A_{nR}^{-1}(0) \leq C_i \leq H_{nR}^{-1}(0)^+\right) > 0 \quad \text{and} \quad M_R\left(A_{nR}^{-1}(0) \leq C_i \leq H_{nR}^{-1}(0)^+\right) \geq 0 \quad \text{[C1]} \qquad \text{(S194)}$$

SI 5.4 $\tilde{N}_R(C_i)$

Since $N_R(C_i)$ involves square root terms and is therefore difficult to analyze directly, we introduce a modified function $\tilde{N}_R(C_i)$ defined as follows:

$$\tilde{N}_R(C_i) = L_R^2(C_i) - M_R^2(C_i) = \tilde{L}_R(C_i) - \tilde{M}_R(C_i) \qquad \text{(S195)}$$

where $\tilde{L}_R(C_i)$ and $\tilde{M}_R(C_i)$ represent the squares of $L_R(C_i)$ and $M_R(C_i)$, respectively, and are given by:

$$\tilde{L}_R(C_i) = L_R^2(C_i) = C_i^2\{C_i - A_{nR}^{-1}(*)\}^2 \qquad \text{(S196)}$$

$$\tilde{M}_R(C_i) = M_R^2(C_i) = \tilde{g}_{slp}^2 H_R(C_i) I_R(C_i) \qquad \text{(S197)}$$

The function $\tilde{N}_R(C_i)$ can also be expressed using $N_R(C_i)$, $L_R(C_i)$, and $M_R(C_i)$ as follows:

$$\tilde{N}_R(C_i) = N_R(C_i)\{L_R(C_i) + M_R(C_i)\} \tag{S198}$$

Eq. (S198) shows that $N_R(C_i)$ and $\tilde{N}_R(C_i)$ share the same roots and signs at C_i where $L_R(C_i) + M_R(C_i) > 0$. Therefore, we have:

$$N_R^{-1}(0) = \tilde{N}_R^{-1}(0) \quad \text{for } C_i \text{ where } L_R(C_i) + M_R(C_i) > 0 \tag{S199}$$

In fact, Eq. (S194) indicates that $L_R(C_i) + M_R(C_i) > 0$ hold true within the interval $A_{nR}^{-1}(0) \leq C_i \leq H_R^{-1}(0)^+$ under condition [C1]. Thus, we proceed to analyze $\tilde{N}_R(C_i)$ in detail in the following sections.

SI 5.4.1 $\tilde{L}_R(C_i)$

Eq. (S196) shows that $\tilde{L}_R(C_i)$ is a quartic function of C_i.

(a) Roots: $\tilde{L}_R^{-1}(0)$

From Eq. (S196), $\tilde{L}_R^{-1}(0)$ is given by:

$$\tilde{L}_R^{-1}(0) = 0 \quad \text{and} \quad A_{nR}^{-1}(*) \tag{S200}$$

(b) $\tilde{L}_R(A_{nR}^{-1}(0))$ and $\tilde{L}_R(H_R^{-1}(0)^+)$

$$\tilde{L}_R(A_{nR}^{-1}(0)) = \{A_{nR}^{-1}(0)\}^2\{A_{nR}^{-1}(0) - A_{nR}^{-1}(*)\}^2 \quad (> 0) \tag{S201}$$

$$\tilde{L}_R(H_R^{-1}(0)^+) = \{H_R^{-1}(0)^+\}^2\{A_{nR}^{-1}(0) - A_{nR}^{-1}(*)\}^2 \quad (> 0) \tag{S202}$$

(c) Geometry of $\tilde{L}_R(C_i)$

Figure S8 and Table S31 illustrate the curve and sign chart of $\tilde{L}_R(C_i)$.

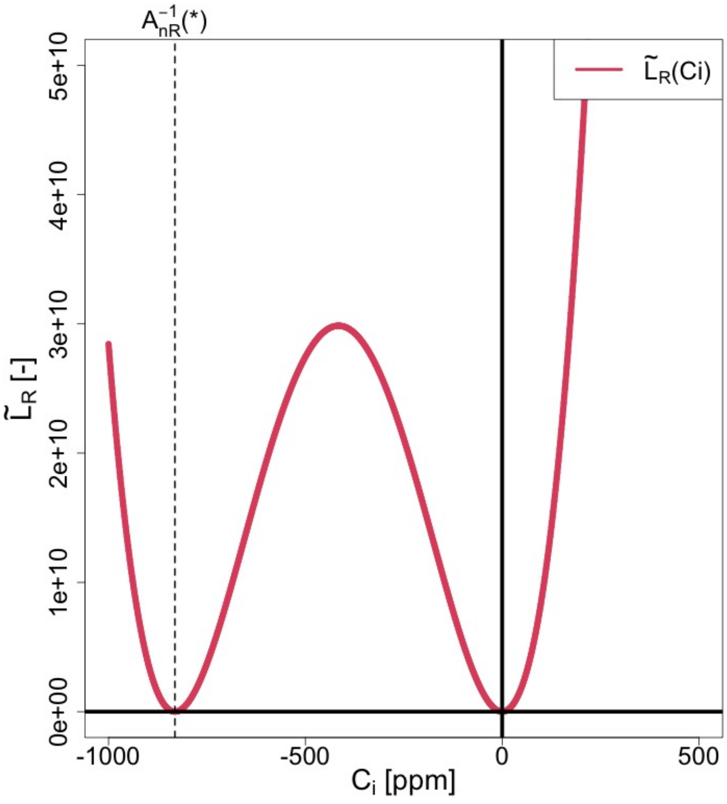

Figure S8: $\tilde{L}_R(C_i)$

Table S31: Sign chart of $\tilde{L}_R(C_i)$

C_i	$-\infty$		$A_{nR}^{-1}(*)$		0		$+\infty$
$\tilde{L}_R$	$+\infty$	$+$	0	$+$	0	$+$	$+\infty$

SI 5.4.2 $\tilde{L}'_R(C_i)$

Differentiating Eq. (S196) with respect to C_i yields:

$$\tilde{L}'_R(C_i) = 4C_i\{C_i - A_{nR}^{-1}(*)\}\{C_i - \frac{A_{nR}^{-1}(*)}{2}\} \tag{S203}$$

Eq. (S203) shows that $\tilde{L}'_R(C_i)$ is a cubic function of C_i.

(a) Roots: $\tilde{L}'^{-1}_R(0)$

From Eq. (S203), $\tilde{L}'^{-1}_R(0)$ is given by:

$$\tilde{L}_R'^{-1}(0) = 0, \quad A_{nR}^{-1}(*), \quad \text{and} \quad \frac{A_{nR}^{-1}(*)}{2} \tag{S204}$$

These values satisfy the following inequality since $A_{nR}^{-1}(*) < 0$ (Eq. (S7)):

$$A_{nR}^{-1}(*) < \frac{A_{nR}^{-1}(*)}{2} < 0 \tag{S205}$$

Since $\tilde{L}'_R(C_i)$ is a cubic function with a positive leading coefficient (Eq. S203), we have the following sign changes for $\tilde{L}'_R(C_i)$:

$$\tilde{L}'_R(C_i < A_{nR}^{-1}(*)) \quad < \quad 0 \tag{S206}$$

$$\tilde{L}'_R(A_{nR}^{-1}(*) < C_i < \frac{A_{nR}^{-1}(*)}{2}) \quad > \quad 0 \tag{S207}$$

$$\tilde{L}'_R\left(\frac{A_{nR}^{-1}(*)}{2} < C_i < 0\right) \quad < \quad 0 \tag{S208}$$

$$\tilde{L}'_R(C_i > 0) \quad > \quad 0 \tag{S209}$$

(b) $\tilde{L}'_R(A_{nR}^{-1}(0))$

$$\tilde{L}'_R(A_{nR}^{-1}(0)) = 4A_{nR}^{-1}(0)\{A_{nR}^{-1}(0) - A_{nR}^{-1}(*)\}\{A_{nR}^{-1}(0) - \frac{A_{nR}^{-1}(*)}{2}\} \tag{S210}$$

(c) Geometry of $\tilde{L}'_R(C_i)$

Figure S9 and Table S32 display the curve and sign chart of $\tilde{L}'_R(C_i)$.

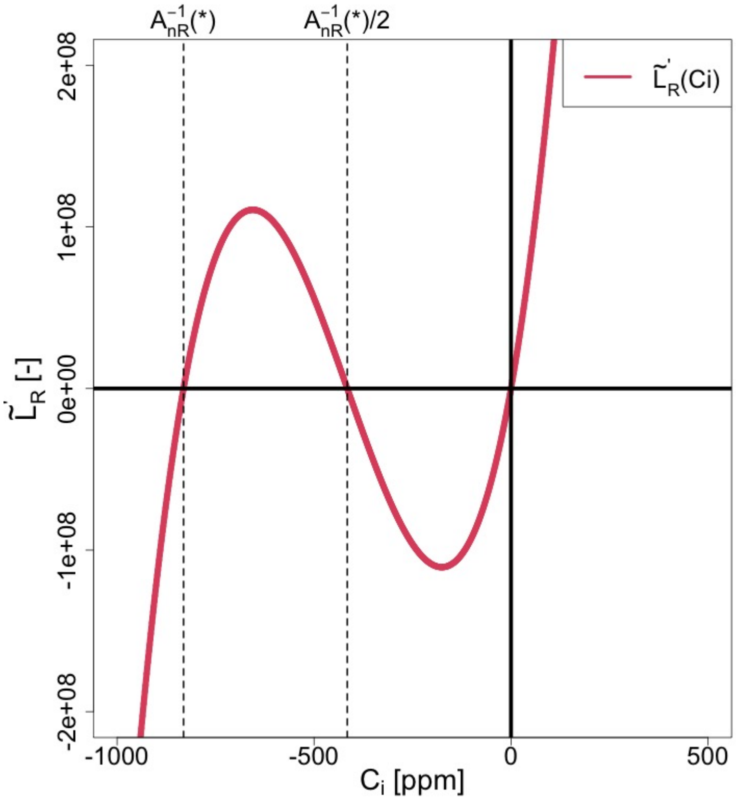

Figure S9: $\tilde{L}'_R(C_i)$

Table S32: Sign chart of $\tilde{L}'_R(C_i)$

C_i	$-\infty$		$A_{nR}^{-1}(*)$		$A_{nR}^{-1}(*)/2$		0		$+\infty$
$\tilde{L}'_R$	$-\infty$	-	0	+	0	-	0	+	$+\infty$

SI 5.4.3 $\tilde{L}''_R(C_i)$

Differentiating Eq. (S203) with respect to C_i gives:

$$\tilde{L}''_R(C_i) = 12C_i^2 - 12A_{nR}^{-1}(*)C_i + 2\{A_{nR}^{-1}(*)\}^2 \tag{S211}$$

Eq. (S211) shows that $\tilde{L}''_R(C_i)$ is a convex quadratic function of C_i.

(a) Roots: $\tilde{L}_R''^{-1}(0)$

From Eq. (S211), $\tilde{L}_R''^{-1}(0)$ is given by:

$$\tilde{L}_R''^{-1}(0)^S = \frac{3+\sqrt{3}}{6} A_{nR}^{-1}(*) \quad (<0) \tag{S212}$$

$$\tilde{L}_R''^{-1}(0)^L = \frac{3-\sqrt{3}}{6} A_{nR}^{-1}(*) \quad (<0) \tag{S213}$$

Note that $\tilde{L}_R''^{-1}(0)^S < \tilde{L}_R''^{-1}(0)^L$ since $A_{nR}^{-1}(*) < 0$ (Eq. (S7)).

Since $\tilde{L}_R''(C_i)$ is a convex quadratic function of C_i, we have:

$$\tilde{L}_R''(C_i < \tilde{L}_R''^{-1}(0)^S) > 0 \tag{S214}$$

$$\tilde{L}_R''(\tilde{L}_R''^{-1}(0)^S < C_i < \tilde{L}_R''^{-1}(0)^L) < 0 \tag{S215}$$

$$\tilde{L}_R''(\tilde{L}_R''^{-1}(0)^L < C_i) > 0 \tag{S216}$$

Since $\tilde{L}_R''^{-1}(0)^L < 0$ (Eq. (S213)), we have in particular:

$$\tilde{L}_R''(0 < C_i) > 0 \tag{S217}$$

(b) $\tilde{L}_R''(0)$

$$\tilde{L}_R''(0) = 2\{A_{nR}^{-1}(*)\}^2 \quad (>0) \tag{S218}$$

(c) Geometry of $\tilde{L}_R''(C_i)$

Figure S10 and Table S33 show the curve and sign chart of $\tilde{L}_R''(C_i)$.

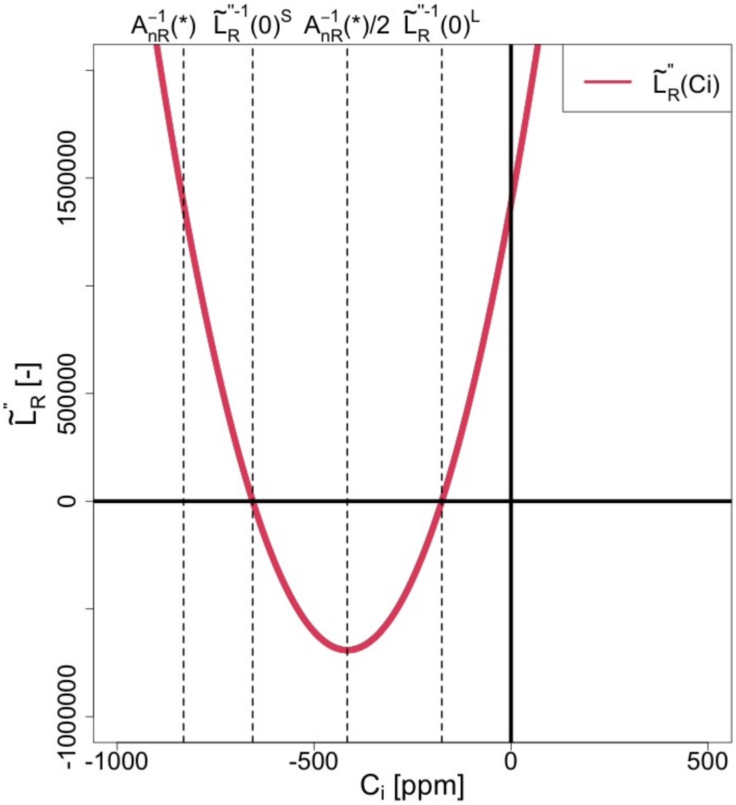

Figure S10: $\tilde{L}''_R(C_i)$

Table S33: Sign chart of $\tilde{L}''_R(C_i)$

C_i	$-\infty$		$\tilde{L}''^{-1}_R(0)^S$		$\tilde{L}''^{-1}_R(0)^L$		0		$+\infty$
$\tilde{L}''_R$	$+\infty$	+	0	-	0	+	+	+	$+\infty$

SI 5.4.4 $\tilde{L}'''_R(C_i)$

Differentiating Eq. (S211) with respect to C_i, we obtain:

$$\tilde{L}'''_R(C_i) = 24\left(C_i - \frac{A^{-1}_{nR}(*)}{2}\right) \tag{S219}$$

Eq. (S219) shows that $\tilde{L}'''_R(C_i)$ is a linear function of C_i with a positive slope.

(a) Root: $\tilde{L}_R'''^{-1}(0)$

From Eq. (S219), $\tilde{L}_R'''^{-1}(0)$ is given by:

$$\tilde{L}_R'''^{-1}(0) = \frac{A_{nR}^{-1}(*)}{2} \quad (< 0) \tag{S220}$$

Since the slope of $\tilde{L}_R'''(C_i)$ is positive (Eq. (S219)), we have the following inequalities:

$$\tilde{L}_R'''\left(C_i < \frac{A_{nR}^{-1}(*)}{2}\right) \;<\; 0 \tag{S221}$$

$$\tilde{L}_R'''\left(\frac{A_{nR}^{-1}(*)}{2} < C_i\right) \;>\; 0 \tag{S222}$$

(b) $\tilde{L}_R'''(0)$

$$\tilde{L}_R'''(0) = -12A_{nR}^{-1}(*) \quad (> 0) \tag{S223}$$

(c) Geometry of $\tilde{L}_R'''(C_i)$

Figure S11 and Table S34 show the curve and sign chart of $\tilde{L}_R'''(C_i)$.

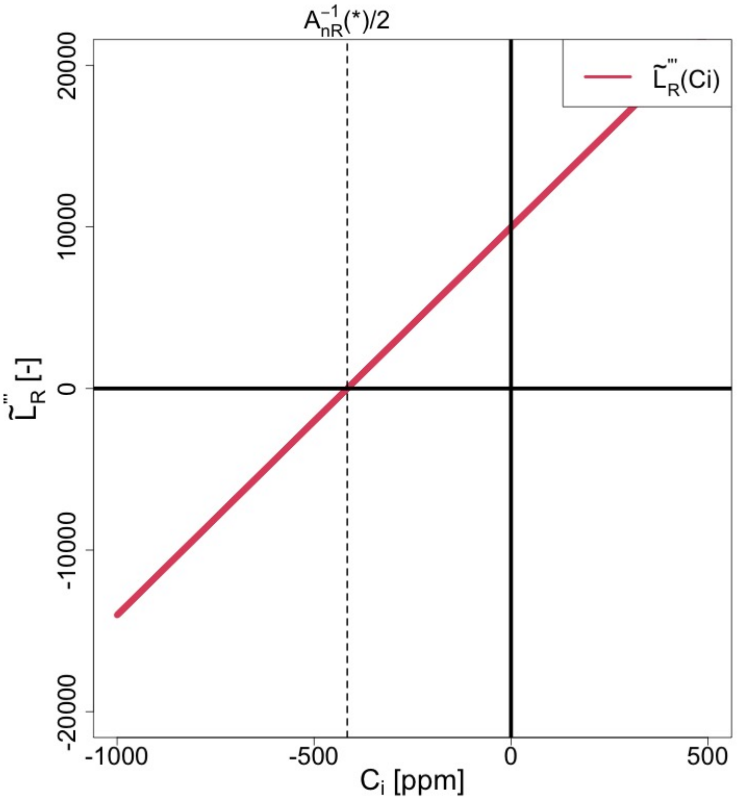

Figure S11: $\tilde{L}_R'''(C_i)$

Table S34: Sign chart of $\tilde{L}_R'''(C_i)$

C_i	$-\infty$		$\tilde{L}_R'''^{-1}(0)$		0		$+\infty$
$\tilde{L}_R'''$	$-\infty$	-	0	+	+	+	$+\infty$

SI 5.4.5 $\tilde{M}_R(C_i)$

Using Eqs. (S33) and (S153) for $H_R(C_i)$ and $I_R(C_i)$, we can rewrite $\tilde{M}_R(C_i)$ from Eq. (S197) as follows:

$$\tilde{M}_R(C_i) = \tilde{g}_{slp}^2 H_R(C_i) I_R(C_i) \tag{S224}$$

$$= \tilde{g}_{slp}^2 \{C_i - H_R^{-1}(0)^+\}\{C_i - H_R^{-1}(0)^-\}\{C_i - I_R^{-1}(0)^L\}\{C_i - I_R^{-1}(0)^S\} \tag{S225}$$

$$= \tilde{g}_{slp}^2 \{C_i^4 + \eta C_i^3 + \theta C_i^2 + \nu C_i + \xi\} \tag{S226}$$

where

$$\eta = -\left\{H_R^{-1}(0)^+ + H_R^{-1}(0)^- + I_R^{-1}(0)^L + I_R^{-1}(0)^S\right\} \tag{S227}$$

$$= -2\left\{C_a + A_{nR}^{-1}(*) - \left\{\lambda_b - \frac{\lambda_s}{2}\right\}\frac{A_{nR}(\pm\infty)}{g_b}\right\} \tag{S228}$$

$$\begin{aligned}\theta = {} & H_R^{-1}(0)^+ H_R^{-1}(0)^- + H_R^{-1}(0)^+ I_R^{-1}(0)^L + H_R^{-1}(0)^+ I_R^{-1}(0)^S \\ & + H_R^{-1}(0)^- I_R^{-1}(0)^L + H_R^{-1}(0)^- I_R^{-1}(0)^S + I_R^{-1}(0)^L I_R^{-1}(0)^S\end{aligned} \tag{S229}$$

$$\nu = -\left\{H_R^{-1}(0)^+ H_R^{-1}(0)^- \{I_R^{-1}(0)^L + I_R^{-1}(0)^S\} + I_R^{-1}(0)^L I_R^{-1}(0)^S \{H_R^{-1}(0)^+ + H_R^{-1}(0)^-\}\right\} \tag{S230}$$

$$\xi = H_R^{-1}(0)^+ H_R^{-1}(0)^- I_R^{-1}(0)^L I_R^{-1}(0)^S \tag{S231}$$

Eq. (S226) shows that $\tilde{M}_R(C_i)$ is a quartic function of C_i.

(c) Root: $\tilde{M}_R^{-1}(0)$

From Eq. (S225), $\tilde{M}_R^{-1}(0)$ is given by:

$$\tilde{M}_R^{-1}(0) = H_R^{-1}(0)^+, H_R^{-1}(0)^-, I_R^{-1}(0)^L, \text{ and } I_R^{-1}(0)^S \tag{S232}$$

(d) $\tilde{M}_R(0)$ and $\tilde{M}_R(A_{nR}^{-1}(0))$

$$\tilde{M}_R(0) = \tilde{g}_{slp}^2 H_R^{-1}(0)^+ H_R^{-1}(0)^- I_R^{-1}(0)^L I_R^{-1}(0)^S \tag{S233}$$

Since $H_R^{-1}(0)^+ H_R^{-1}(0)^- < 0$ (Eq. (S35)) and $I_R^{-1}(0)^L > 0$ under [C1] (Eq. (S152)), we have:

$$\tilde{M}_R(0)\begin{cases} > 0 & [\mathrm{C1}, I_R^{-1}(0)^S < 0] \\ < 0 & [\mathrm{C1}, I_R^{-1}(0)^S > 0] \\ = 0 & [\mathrm{C1}, I_R^{-1}(0)^S = 0]\end{cases} \tag{S234}$$

Eq. (S234) indicates that the signs of $\tilde{M}_R(0)$ are opposite to those of $I_R^{-1}(0)^S$ under [C1]. Note that the signs of $I_R^{-1}(0)^S$ are the same as those of $\tilde{\gamma}$ under [C1] (Eq. (S157)). Therefore, the signs of $\tilde{M}_R(0)$ are also opposite to those of $\tilde{\gamma}$ under [C1]. Thus, we have the following relationship under [C1]:

$$\mathrm{sgn}(\tilde{\gamma}) = \mathrm{sgn}(I_R^{-1}(0)^S) = -\mathrm{sgn}(\tilde{M}_R(0)) \quad [\mathrm{C1}] \tag{S235}$$

Using Eqs. (S41) and (S145), we have:

$$\tilde{M}_R(A_{nR}^{-1}(0)) = \tilde{g}_{slp}^2\{A_{nR}^{-1}(0) - C_a\}^2\{A_{nR}^{-1}(0) - A_{nR}^{-1}(*)\}^2 \quad (> 0) \tag{S236}$$

(e) Geometry of $\tilde{M}_R(C_i)$

Figures S12 to S14 and Tables S35 to S37 show the curves and sign charts of $\tilde{M}_R(C_i)$ for cases [C1, $I_R^{-1}(0)^S < 0$], [C1, $I_R^{-1}(0)^S > 0$], and [C1, $I_R^{-1}(0)^S = 0$],.

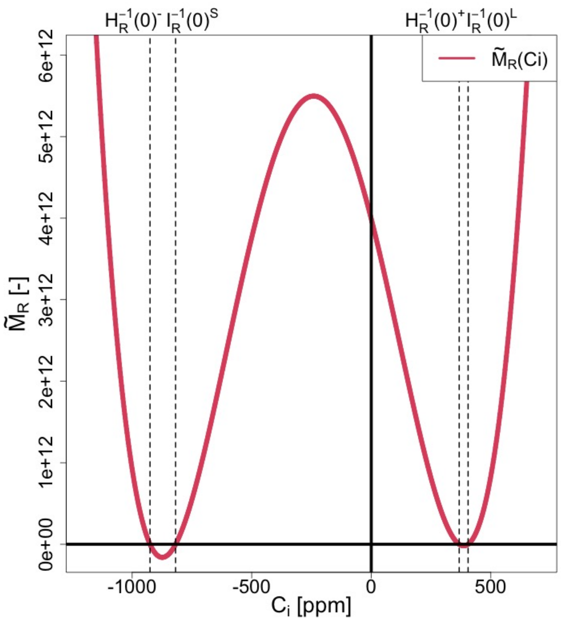

(a) $-1200 < C_i < 700$

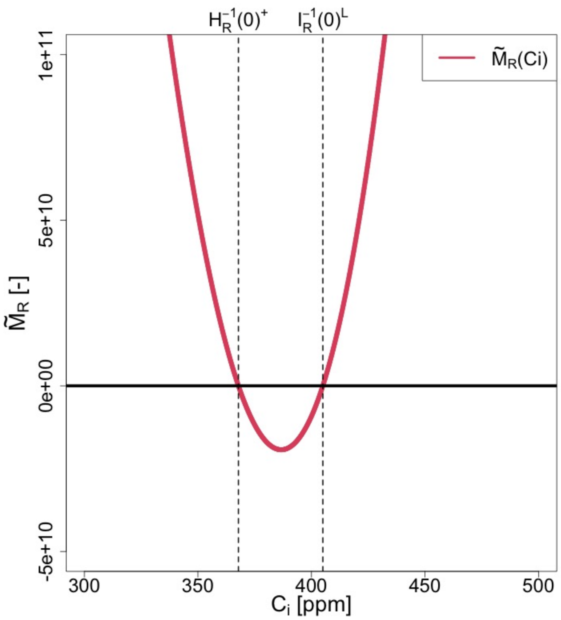

(b)$300 < C_i < 500$

Figure S12: $\tilde{M}_R(C_i)$ [C1, $I_R^{-1}(0)^S < 0$]

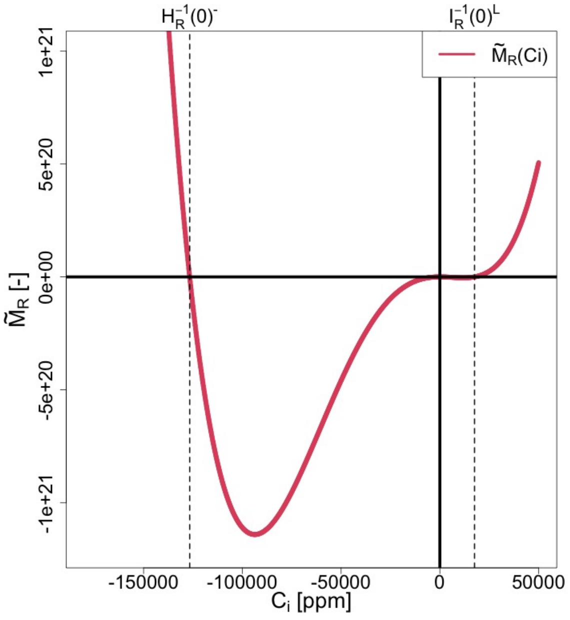

(a) $-180000 < C_i < 50000$

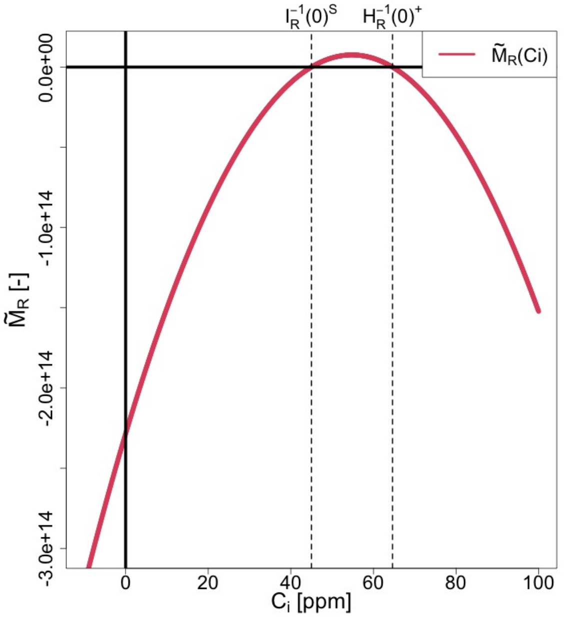

(b) $-10 < C_i < 100$

Figure S13: $\tilde{M}_R(C_i)$ [C1, $I_R^{-1}(0)^S > 0$]

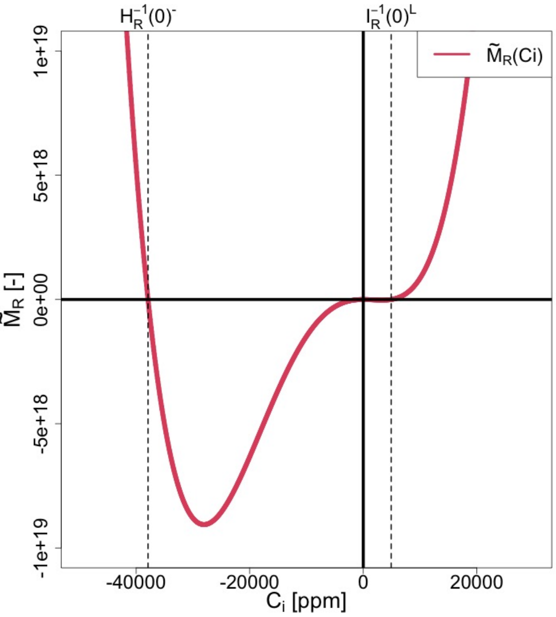

(a) $-50000 < C_i < 30000$

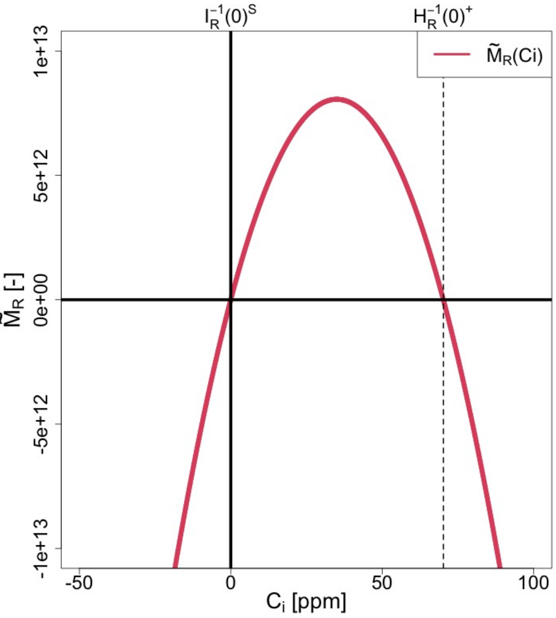

(b) $-50 < C_i < 100$

Figure S14: $\tilde{M}_R(C_i)$ [C1, $I_R^{-1}(0)^S = 0$]

Table S35: Sign chart of $\tilde{M}_R(C_i)$ [C1, $I_R^{-1}(0)^S < 0$]

C_i	$-\infty$		$H_R^{-1}(0)^-$		$A_{nR}^{-1}(*)$		$I_R^{-1}(0)^S$		0		$A_{nR}^{-1}(0)$		$H_R^{-1}(0)^+$		C_a		$I_R^{-1}(0)^L$		$+\infty$
$\tilde{M}_R$	$+\infty$	+	0	-	-	-	0	+	+	+	+	+	0	-	-	-	0	+	$+\infty$

Table S36: Sign chart of $\tilde{M}_R(C_i)$ [C1, $I_R^{-1}(0)^S > 0$]

C_i	$-\infty$		$H_R^{-1}(0)^-$		$A_{nR}^{-1}(*)$		0		$I_R^{-1}(0)^S$		$A_{nR}^{-1}(0)$		$H_R^{-1}(0)^+$		C_a		$I_R^{-1}(0)^L$		$+\infty$
$\tilde{M}_R$	$+\infty$	+	0	-	-	-	-	-	0	+	+	+	0	-	-	-	0	+	$+\infty$

Table S37: Sign chart of $\tilde{M}_R(C_i)$ [C1, $I_R^{-1}(0)^S = 0$]

C_i	$-\infty$		$H_R^{-1}(0)^-$		$A_{nR}^{-1}(*)$		$I_R^{-1}(0)^S = 0$		$A_{nR}^{-1}(0)$		$H_R^{-1}(0)^+$		C_a		$I_R^{-1}(0)^L$		$+\infty$
$\tilde{M}_R$	$+\infty$	+	0	-	-	-	0	+	+	+	0	-	-	-	0	+	$+\infty$

SI 5.4.6 $\tilde{M}_R'(C_i)$

By differentiating Eq. (S224) or (S226) with respect to C_i, we have:

$$\tilde{M}_R'(C_i) = \tilde{g}_{slp}^2 \left\{ H_R(C_i) I_R'(C_i) + H_R'(C_i) I_R(C_i) \right\} \tag{S237}$$

$$= \tilde{g}_{slp}^2 \left\{ 4C_i^3 + 3\eta C_i^2 + 2\theta C_i + \nu \right\} \tag{S238}$$

Eq. (S238) shows that $\tilde{M}_R'(C_i)$ is a cubic function of C_i.

(a) $\tilde{M}_R'(H_R^{-1}(0)^-)$, $\tilde{M}_R'(I_R^{-1}(0)^S)$, $\tilde{M}_R'(H_R^{-1}(0)^+)$, and $\tilde{M}_R'(I_R^{-1}(0)^L)$

From Eq. (S237), we have:

$$\tilde{M}_R'(H_R^{-1}(0)^-) = \tilde{g}_{slp}^2 H_R'(H_R^{-1}(0)^-) I_R(H_R^{-1}(0)^-) \tag{S239}$$

$$\tilde{M}_R'(I_R^{-1}(0)^S) = \tilde{g}_{slp}^2 H_R(I_R^{-1}(0)^S) I_R'(I_R^{-1}(0)^S) \tag{S240}$$

$$\tilde{M}_R'(H_R^{-1}(0)^+) = \tilde{g}_{slp}^2 H_R'(H_R^{-1}(0)^+) I_R(H_R^{-1}(0)^+) \tag{S241}$$

$$\tilde{M}_R'(I_R^{-1}(0)^L) = \tilde{g}_{slp}^2 H_R(I_R^{-1}(0)^L) I_R'(I_R^{-1}(0)^L). \tag{S242}$$

The signs of Eqs. (S239) to (S242) under [C1] are given as:

$$\tilde{M}_R'(H_R^{-1}(0)^-) < 0 \quad [\text{C1}] \tag{S243}$$

$$\tilde{M}_R'(I_R^{-1}(0)^S) > 0 \quad [\text{C1}] \tag{S244}$$

$$\tilde{M}_R'(H_R^{-1}(0)^+) < 0 \quad [\text{C1}] \tag{S245}$$

$$\tilde{M}_R'(I_R^{-1}(0)^L) > 0 \quad [\text{C1}] \tag{S246}$$

These signs are derived using Eqs. (S36) to (S38), (S83), (S84), (S158) to (S160), (S162), (S163), and (S170) to (S172) under [C1].

(b) Roots: $\tilde{M}_R'^{-1}(0)$

Since $H_R^{-1}(0)^- < I_R^{-1}(0)^S < H_R^{-1}(0)^+ < I_R^{-1}(0)^L$ under [C1] (Eqs. (S170) to (S172)), Eqs. (S243) to (S246) indicate that there are three roots for $\tilde{M}_R'(C_i) = 0$ at $H_R^{-1}(0)^- < C_i < I_R^{-1}(0)^S$, $I_R^{-1}(0)^S < C_i < H_R^{-1}(0)^+$, and $H_R^{-1}(0)^+ < C_i < I_R^{-1}(0)^L$. To distinguish them, these are expressed as:

$$\tilde{M}_R'^{-1}(0) = \tilde{M}_R'^{-1}(0)^S, \tilde{M}_R'^{-1}(0)^M, \text{ and } \tilde{M}_R'^{-1}(0)^L \tag{S247}$$

which follow the inequality:

$$H_R^{-1}(0)^- < \tilde{M}_R'^{-1}(0)^S < I_R^{-1}(0)^S < \tilde{M}_R'^{-1}(0)^M < H_R^{-1}(0)^+ < \tilde{M}_R'^{-1}(0)^L < I_R^{-1}(0)^L \quad [\text{C1}] \tag{S248}$$

Considering that the coefficient of C_i^3 for $\tilde{M}_R'(C_i)$ is positive (Eq. (S238)), we have:

$$\tilde{M}_R'(C_i < \tilde{M}_R'^{-1}(0)^S) < 0 \tag{S249}$$

$$\tilde{M}_R'(\tilde{M}_R'^{-1}(0)^S < C_i < \tilde{M}_R'^{-1}(0)^M) > 0 \tag{S250}$$

$$\tilde{M}_R'(\tilde{M}_R'^{-1}(0)^M < C_i < \tilde{M}_R'^{-1}(0)^L) < 0 \tag{S251}$$

$$\tilde{M}_R'(\tilde{M}_R'^{-1}(0)^L < C_i) > 0 \tag{S252}$$

(c) $\tilde{M}_R'(A_{nR}^{-1}(0))$

$$\tilde{M}_R'(A_{nR}^{-1}(0)) = 4\tilde{g}_{slp}^2\{A_{nR}^{-1}(0) - C_a\}\{A_{nR}^{-1}(0) - A_{nR}^{-1}(*)\}\{A_{nR}^{-1}(0) + \frac{\eta}{4}\} \tag{S253}$$

Since $A_{nR}^{-1}(0) < C_a$ and $A_{nR}^{-1}(0) > A_{nR}^{-1}(*)$ under [C1] (Table 3), we have:

$$\tilde{M}_R'(A_{nR}^{-1}(0)) \begin{cases} < 0 & \left[\text{C1}, A_{nR}^{-1}(0) + \frac{\eta}{4} > 0\right] \\ > 0 & \left[\text{C1}, A_{nR}^{-1}(0) + \frac{\eta}{4} < 0\right] \\ = 0 & \left[\text{C1}, A_{nR}^{-1}(0) + \frac{\eta}{4} = 0\right] \end{cases} \tag{S254}$$

Eq. (S254) shows that the signs of $\tilde{M}_R'(A_{nR}^{-1}(0))$ are opposite to those of $A_{nR}^{-1}(0) + \frac{\eta}{4}$ under [C1]. Thus, we have:

$$\text{sgn}(\tilde{M}_R'(A_{nR}^{-1}(0))) = -\text{sgn}\left(A_{nR}^{-1}(0) + \frac{\eta}{4}\right) \quad [\text{C1}] \tag{S255}$$

(d) Inequality relation between $A_{nR}^{-1}(0)$ and $\tilde{M}_R'^{-1}(0)^M$

From the signs of $\tilde{M}_R'(A_{nR}^{-1}(0))$, we can derive the inequality relation between $A_{nR}^{-1}(0)$ and $\tilde{M}_R'^{-1}(0)^M$. We focus on the range $I_R^{-1}(0)^S < C_i < H_R^{-1}(0)^+$, since both $A_{nR}^{-1}(0)$ and $\tilde{M}_R'^{-1}(0)^M$ line within the range (Eqs. (S170) to (S172), and (S248)).

For $\tilde{M}_R'(A_{nR}^{-1}(0)) < 0$, i.e., $A_{nR}^{-1}(0) + \frac{\eta}{4} > 0$ under [C1] (Eq. (S255)), we have $\tilde{M}_R'^{-1}(0)^M < A_{nR}^{-1}(0)$ from Eqs. (S250) and (S251). Similarly, we have:

$$I_R^{-1}(0)^S < \tilde{M}_R'^{-1}(0)^M < A_{nR}^{-1}(0) < H_R^{-1}(0)^+ \quad \left[\text{C1}, A_{nR}^{-1}(0) + \frac{\eta}{4} > 0\right] \tag{S256}$$

$$I_R^{-1}(0)^S < A_{nR}^{-1}(0) < \tilde{M}_R'^{-1}(0)^M < H_R^{-1}(0)^+ \quad \left[\text{C1}, A_{nR}^{-1}(0) + \frac{\eta}{4} < 0\right] \tag{S257}$$

$$I_R^{-1}(0)^S < A_{nR}^{-1}(0) = \tilde{M}_R'^{-1}(0)^M < H_R^{-1}(0)^+ \quad \left[\text{C1}, A_{nR}^{-1}(0) + \frac{\eta}{4} = 0\right] \tag{S258}$$

(e) Geometry of $\tilde{M}'_R(C_i)$

Figures S15 to S17 show the curves of $\tilde{M}'_R(C_i)$, and Tables S38 to S40 show the sign charts of $\tilde{M}'_R(C_i)$ for each inequality pattern from Eqs. (S256) to (S258) over the interval $A_{nR}^{-1}(0) < C_i < H_R^{-1}(0)^+$, which is the possible range of appropriate solutions under [C1] (Table 4). We note that $A_{nR}^{-1}(0) = F_R^{-1}(0)$ and $H_R^{-1}(0)^+ = F_R^{-1}(*)^+$ (Eqs. (S93) and (S87)).

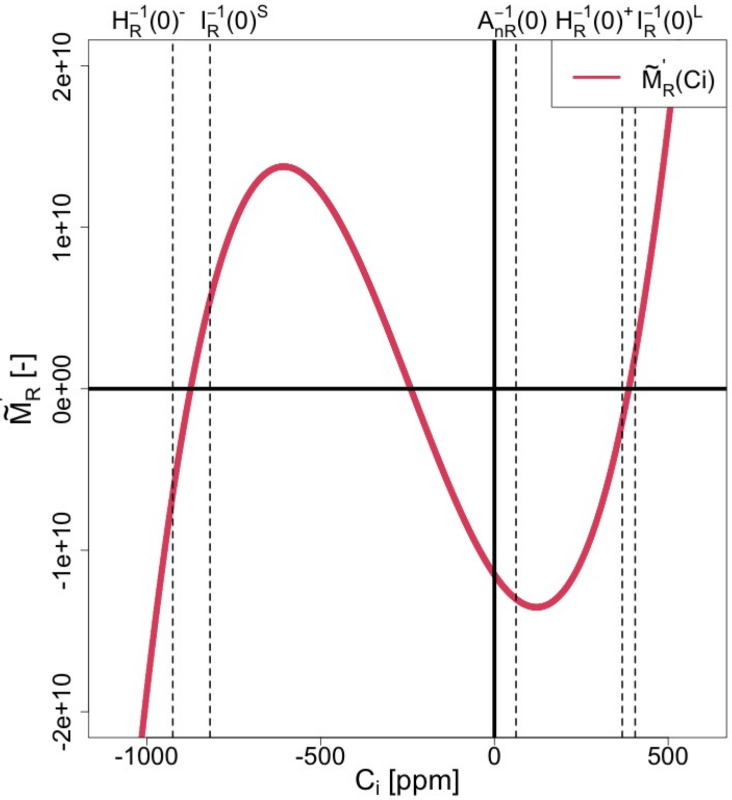

Figure S15: $\tilde{M}'_R(C_i)$ [C1, $A_{nR}^{-1}(0) + \frac{\eta}{4} > 0$]

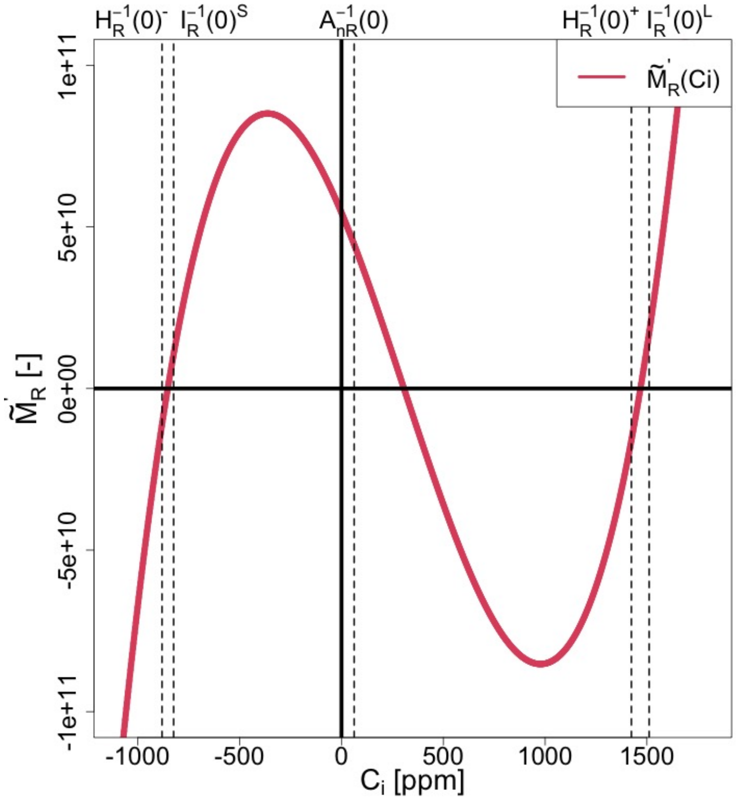

Figure S16: $\tilde{M}_R'(C_i)$ [C1, $A_{nR}^{-1}(0) + \frac{\eta}{4} < 0$]

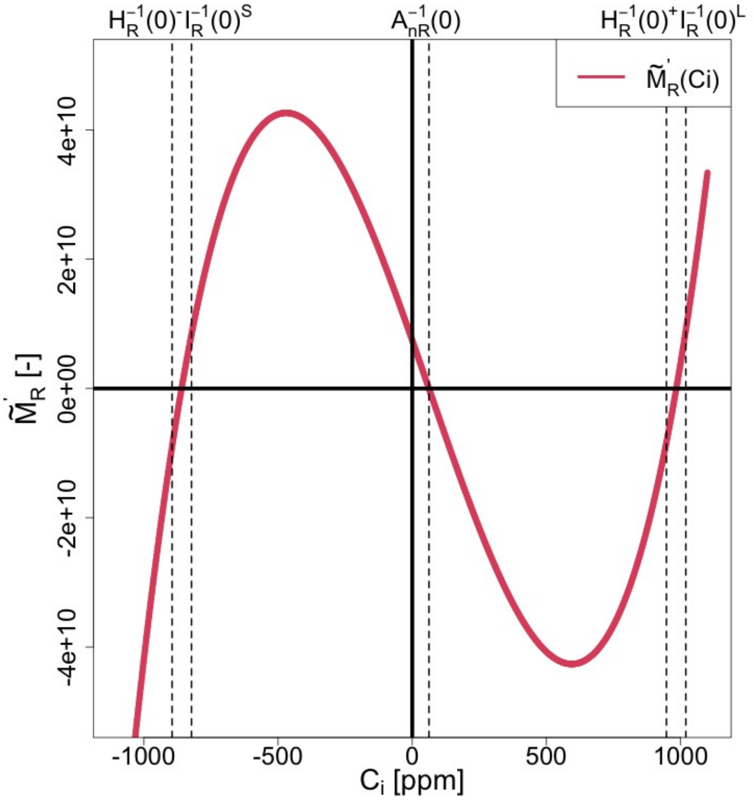

Figure S17: $\tilde{M}'_R(C_i)$ [C1, $A_{nR}^{-1}(0) + \frac{\eta}{4} = 0$]

Table S38: Sign chart of $\tilde{M}'_R(C_i)$ [C1, $A_{nR}^{-1}(0) + \frac{\eta}{4} > 0$]

C_i	$A_{nR}^{-1}(0)$		$H_R^{-1}(0)^+$
$\tilde{M}'_R$	-	-	-

Table S39: Sign chart of $\tilde{M}'_R(C_i)$ [C1, $A_{nR}^{-1}(0) + \frac{\eta}{4} < 0$]

C_i	$A_{nR}^{-1}(0)$		$\tilde{M}_R'^{-1}(0)^M$		$H_R^{-1}(0)^+$
$\tilde{M}'_R$	+	+	0	-	-

Table S40: Sign chart of $\tilde{M}'_R(C_i)$ [C1, $A_{nR}^{-1}(0) + \frac{\eta}{4} = 0$]

C_i	$\tilde{M}_R'^{-1}(0)^M = A_{nR}^{-1}(0)$		$H_R^{-1}(0)^+$
$\tilde{M}'_R$	0	-	-

(g) Sign of $\tilde{M}'_R(0)$

From Eq. (S238), we have

$$\tilde{M}'_R(0) = \tilde{g}_{slp}^2 \nu \tag{S259}$$

We aim to prove the following proposition regarding the sign of $\tilde{M}'_R(0)$.

Proposition 1. $\tilde{M}'_R(0) > 0$ *under [C1] and* $A_{nR}^{-1}(0) + \frac{\eta}{4} < 0$.

Proof. We separate this proof into two cases: $\tilde{\gamma} \leq 0$ and $\tilde{\gamma} > 0$.

(A): In the case of $\tilde{\gamma} \leq 0$

Under $A_{nR}^{-1}(0) + \frac{\eta}{4} < 0$ and [C1], Eqs. (S248) and (S257) show

$$\tilde{M}_R'^{-1}(0)^S < A_{nR}^{-1}(0) < \tilde{M}_R'^{-1}(0)^M \quad [\text{C1}, A_{nR}^{-1}(0) + \frac{\eta}{4} < 0] \tag{S260}$$

For $\tilde{\gamma} \leq 0$, Eq. (S235) shows

$$I_R^{-1}(0)^S \leq 0 \quad [\text{C1}, \tilde{\gamma} \leq 0] \tag{S261}$$

By combining $A_{nR}^{-1}(0) > 0$ under [C1] (Table 3) with Eqs. (S248), (S260) and (S261), we have

$$\tilde{M}_R'^{-1}(0)^S < I_R^{-1}(0)^S \leq 0 < A_{nR}^{-1}(0) < \tilde{M}_R'^{-1}(0)^M \quad [\text{C1}, A_{nR}^{-1}(0) + \frac{\eta}{4} < 0, \tilde{\gamma} \leq 0] \tag{S262}$$

Hence we have

$$\tilde{M}_R'^{-1}(0)^S < 0 < \tilde{M}_R'^{-1}(0)^M \quad [\text{C1}, A_{nR}^{-1}(0) + \frac{\eta}{4} < 0, \tilde{\gamma} \leq 0] \tag{S263}$$

Eqs. (S263) and (S250) show

$$\tilde{M}'_R(0) > 0 \quad [\text{C1}, A_{nR}^{-1}(0) + \frac{\eta}{4} < 0, \tilde{\gamma} \leq 0] \tag{S264}$$

(B): In the case of $\tilde{\gamma} > 0$

First, we derive an inequality relation for $A_{nR}^{-1}(0)$ and $A_{nR}^{-1}(*)$ from the conditions $\tilde{\gamma} > 0$ and $A_{nR}^{-1}(0) + \frac{\eta}{4} < 0$. Since $\tilde{\gamma} > 0$, this provides an upper limit for C_a, as follows:

$$\tilde{\gamma} > 0 \iff C_a A_{nR}^{-1}(*) - \{\lambda_b - \lambda_s\} \frac{A_{nR}(\pm\infty)}{g_b} A_{nR}^{-1}(0) > 0 \tag{S265}$$

$$\iff C_a < \{\lambda_b - \lambda_s\} \frac{A_{nR}(\pm\infty)}{g_b} \frac{A_{nR}^{-1}(0)}{A_{nR}^{-1}(*)} \tag{S266}$$

To derive Eq. (S266), $A_{nR}^{-1}(*) < 0$ was used (Eq. (S7)).

On the other hand, $A_{nR}^{-1}(0) + \frac{\eta}{4} < 0$ provides a lower limit for C_a:

$$A_{nR}^{-1}(0) + \frac{\eta}{4} < 0 \iff A_{nR}^{-1}(0) - \frac{1}{2}\left\{C_a + A_{nR}^{-1}(*) - \left\{\lambda_b - \frac{\lambda_s}{2}\right\}\frac{A_{nR}(\pm\infty)}{g_b}\right\} < 0 \tag{S267}$$

$$\iff 2A_{nR}^{-1}(0) - A_{nR}^{-1}(*) + \left\{\lambda_b - \frac{\lambda_s}{2}\right\}\frac{A_{nR}(\pm\infty)}{g_b} < C_a \tag{S268}$$

Combining Eqs. (S266) and (S268), we have

$$2A_{nR}^{-1}(0) - A_{nR}^{-1}(*) + \left\{\lambda_b - \frac{\lambda_s}{2}\right\}\frac{A_{nR}(\pm\infty)}{g_b} < \{\lambda_b - \lambda_s\}\frac{A_{nR}(\pm\infty)}{g_b}\frac{A_{nR}^{-1}(0)}{A_{nR}^{-1}(*)} \tag{S269}$$

$$\iff \left\{2A_{nR}^{-1}(0) - A_{nR}^{-1}(*)\right\}\frac{g_b}{A_{nR}(\pm\infty)} < \left\{-\lambda_b + \frac{\lambda_s}{2} + \{\lambda_b - \lambda_s\}\frac{A_{nR}^{-1}(0)}{A_{nR}^{-1}(*)}\right\} \quad [C1, A_{nR}^{-1}(0) + \frac{\eta}{4} < 0, \tilde{\gamma} > 0] \tag{S270}$$

To derive Eq. (S270), $A_{nR}(\pm\infty) > 0$ under [C1] was used (Table 3).

Since the term on the left-hand side of Eq. (S270) is positive under [C1] (Table 3), the term on the right-hand side of Eq. (S270) is also positive, as follows:

$$0 < \left\{-\lambda_b + \frac{\lambda_s}{2} + \{\lambda_b - \lambda_s\}\frac{A_{nR}^{-1}(0)}{A_{nR}^{-1}(*)}\right\} \tag{S271}$$

$$\iff \frac{A_{nR}^{-1}(0)}{A_{nR}^{-1}(*)} < -3 \quad [\mathrm{C1}, A_{nR}^{-1}(0) + \frac{\eta}{4} < 0, \tilde{\gamma} > 0] \tag{S272}$$

Eq. (S272) provides an upper limit for $\frac{A_{nR}^{-1}(0)}{A_{nR}^{-1}(*)}$.

Next, we examine the sign of ν to determine the sign of $\tilde{M}_R'(0)$ (Eq. (S259)). From Eq. (S230), we can consider ν as a quadratic function with respect to C_a, given by

$$\begin{aligned}\nu(C_a) =& -2A_{nR}^{-1}(*)C_a^2 - \left\{2\{A_{nR}^{-1}(*)\}^2 - \{2\lambda_b - \lambda_s\}\frac{A_{nR}(\pm\infty)}{g_b}\{A_{nR}^{-1}(*) + A_{nR}^{-1}(0)\}\right\}C_a \\ &+ \{2\lambda_b - \lambda_s\}\frac{A_{nR}(\pm\infty)}{g_b}\{A_{nR}^{-1}(*)A_{nR}^{-1}(0)\} - 2\lambda_b\{\lambda_b - \lambda_s\}\left\{\frac{A_{nR}(\pm\infty)}{g_b}\right\}^2 A_{nR}^{-1}(0)\end{aligned} \tag{S273}$$

Eq. (S273) shows that $\nu(C_a)$ is a convex quadratic function of C_i since $A_{nR}^{-1}(*) < 0$ (Eq. S7).

$\nu^{-1}(\mathrm{min})$ represents C_a, where $\nu(C_a)$ is minimized, given by

$$\nu^{-1}(\mathrm{min}) = -\frac{1}{2}\left\{A_{nR}^{-1}(*) - \{\lambda_b - \frac{\lambda_s}{2}\}\frac{A_{nR}(\pm\infty)}{g_b}\{1 + \frac{A_{nR}^{-1}(0)}{A_{nR}^{-1}(*)}\}\right\} \tag{S274}$$

$$< -\frac{1}{2}\left\{2\{\lambda_b - \frac{\lambda_s}{2}\}\frac{A_{nR}(\pm\infty)}{g_b} - \{-A_{nR}^{-1}(*)\}\right\} \quad [\mathrm{C1}, A_{nR}^{-1}(0) + \frac{\eta}{4} < 0, \tilde{\gamma} > 0] \tag{S275}$$

To derive the inequality in Eq. (S275), Eq. (S272) was used. To explore the sign of $\nu^{-1}(\mathrm{min})$, we compare the first and second terms in the right-hand side of Eq. (S275). From Eq. (S266), we have

$$-A_{nR}^{-1}(*) < -\{\lambda_b - \lambda_s\}\frac{A_{nR}(\pm\infty)}{g_b}\frac{A_{nR}^{-1}(0)}{C_a} \quad [\tilde{\gamma} > 0] \tag{S276}$$

$$< -\{\lambda_b - \lambda_s\}\frac{A_{nR}(\pm\infty)}{g_b} \quad [\mathrm{C1}, \tilde{\gamma} > 0] \tag{S277}$$

$$< 2\{\lambda_b - \frac{\lambda_s}{2}\}\frac{A_{nR}(\pm\infty)}{g_b} \quad [\mathrm{C1}, \tilde{\gamma} > 0] \tag{S278}$$

In deriving Eq. (S277), we used $C_a > A_{nR}^{-1}(0)$, which holds under [C1]. For Eq. (S278), we used $\lambda_b = 1.4$ and $\lambda_s = 1.6$. Eq. (S278) shows the inequality relation between the first and second terms on the right-hand side of Eq. (S275). Thus we have

$$\nu^{-1}(\mathrm{min}) < 0 \quad [\mathrm{C1}, A_{nR}^{-1}(0) + \frac{\eta}{4} < 0, \tilde{\gamma} > 0] \tag{S279}$$

Next, we examine the sign of $\nu(0)$. From Eq. (S273), $\nu(0)$ is given as

$$\nu(0) = \frac{\{2\lambda_b - \lambda_s\}A_{nR}^{-1}(0)A_{nR}^{-1}(\pm\infty)}{g_b}\left\{-\frac{2\lambda_b\{\lambda_b - \lambda_s\}}{\{2\lambda_b - \lambda_s\}}\frac{A_{nR}(\pm\infty)}{g_b} - \{-A_{nR}^{-1}(*)\}\right\} \tag{S280}$$

Following similar steps to derive Eq. (S278), we have

$$-A_{nR}^{-1}(*) < -\frac{2\lambda_b\{\lambda_b - \lambda_s\}}{\{2\lambda_b - \lambda_s\}}\frac{A_{nR}(\pm\infty)}{g_b} \quad [\mathrm{C1}, \tilde{\gamma} > 0] \tag{S281}$$

Eq. (S281) implies

$$\nu(0) > 0 \quad [\mathrm{C1}, \tilde{\gamma} > 0] \tag{S282}$$

Since $\nu(C_a)$ is a convex quadratic function of C_a (Eq. (S273)), Eqs. (S279) and (S282) lead to

$$\nu(C_a > 0) > 0 \quad [\mathrm{C1}, A_{nR}^{-1}(0) + \frac{\eta}{4} < 0, \tilde{\gamma} > 0] \tag{S283}$$

Figure S18 shows the curve of $\nu(C_a)$.

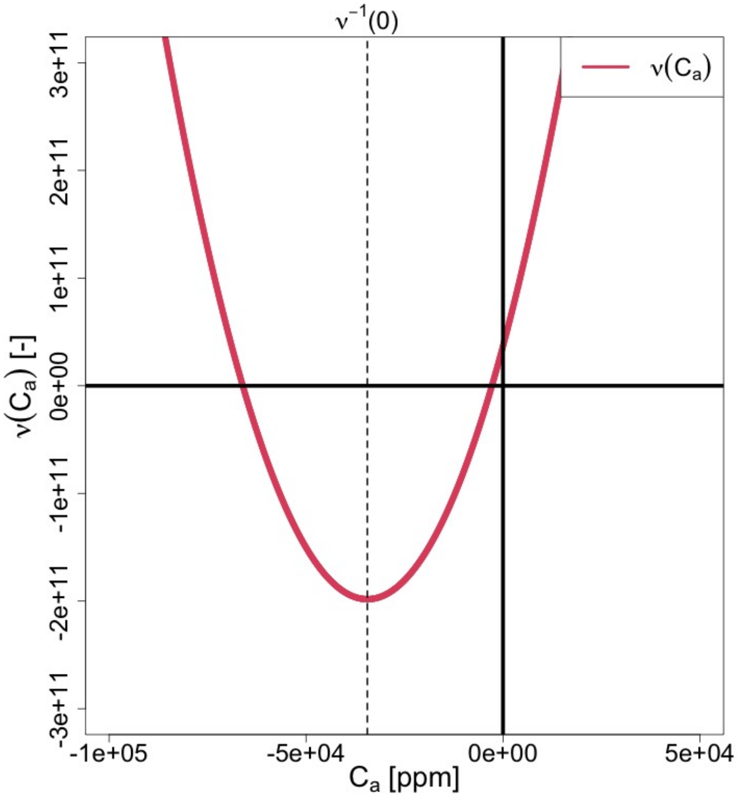

Figure S18: $\nu(C_a)$

Since $C_a > 0$, Eqs. (S259) and (S283) show

$$\tilde{M}_R'(0) > 0 \quad [\text{C1, and } A_{nR}^{-1}(0) + \frac{\eta}{4} < 0, \tilde{\gamma} > 0] \tag{S284}$$

Eqs. (S264) and (S284) show

$$\tilde{M}_R'(0) > 0 \quad [\text{C1}, A_{nR}^{-1}(0) + \frac{\eta}{4} < 0] \tag{S285}$$

Thus, the proposition is proven. □

SI 5.4.7 $\tilde{M}_R''(C_i)$

By differentiating Eq. (S237) or (S238) with respect to C_i, we have

$$\tilde{M}_R''(C_i) = 2\tilde{g}_{slp}^2\{H_R(C_i) + I_R(C_i) + H_R'(C_i)I_R'(C_i)\} \tag{S286}$$

$$= \tilde{g}_{slp}^2\{12C_i^2 + 6\eta C_i + 2\theta\} \tag{S287}$$

$$= 12\tilde{g}_{slp}^2 \left\{\left\{C_i + \frac{\eta}{4}\right\}^2 - \frac{\eta^2}{16} + 2\theta\right\} \tag{S288}$$

Eq. (S288) shows that $\tilde{M}_R''(C_i)$ is a convex quadratic function of C_i and has its minimum at $C_i = -\frac{\eta}{4}$, which is expressed as

$$\tilde{M}_R''^{-1}(\text{min}) = -\frac{\eta}{4} \tag{S289}$$

(a) Root: $\tilde{M}_R''^{-1}(0)$

Since there are three roots for $\tilde{M}_R'(C_i) = 0$ (Eq. (S247)), there must be two roots for $\tilde{M}_R''(C_i) = 0$. These roots are expressed as

$$\tilde{M}_R''^{-1}(0) = \tilde{M}_R''^{-1}(0)^S \text{ and } \tilde{M}_R''^{-1}(0)^L, \tag{S290}$$

which follow the inequality:

$$\tilde{M}_R'^{-1}(0)^S < \tilde{M}_R''^{-1}(0)^S < \tilde{M}_R'^{-1}(0)^M < \tilde{M}_R''^{-1}(0)^L < \tilde{M}_R'^{-1}(0)^L \tag{S291}$$

Since $\tilde{M}_R''(C_i)$ is a convex quadratic function of C_i (Eq. (S287)), we have

$$\tilde{M}_R''(C_i < \tilde{M}_R''^{-1}(0)^S) > 0 \tag{S292}$$

$$\tilde{M}_R''(\tilde{M}_R''^{-1}(0)^S < C_i < \tilde{M}_R''^{-1}(0)^L) < 0 \tag{S293}$$

$$\tilde{M}_R''(\tilde{M}_R''^{-1}(0)^L < C_i) > 0 \tag{S294}$$

Since $\tilde{M}_R''^{-1}(\text{min})$ must be in the range $\tilde{M}_R''^{-1}(0)^S < C_i < \tilde{M}_R''^{-1}(0)^L$, Eq. (S289) leads to:

$$\tilde{M}_R'^{-1}(0)^S < \tilde{M}_R''^{-1}(0)^S < -\frac{\eta}{4} < \tilde{M}_R''^{-1}(0)^L < \tilde{M}_R'^{-1}(0)^L \tag{S295}$$

(b) $\tilde{M}_R''(\tilde{M}_R'^{-1}(0)^M)$ and $\tilde{M}_R''\left(-\frac{\eta}{4}\right)$

From Eqs. (S291), (S293), and (S295), we have

$$\tilde{M}_R''(\tilde{M}_R'^{-1}(0)^M) \quad < \quad 0 \tag{S296}$$

$$\tilde{M}_R''\left(-\frac{\eta}{4}\right) \quad < \quad 0 \tag{S297}$$

(c) Inequality relationship between $-\frac{\eta}{4}$ and $\tilde{M}_R'^{-1}(0)^M$

Eq. (S295) shows that $-\frac{\eta}{4}$ exists within the range $\tilde{M}_R'^{-1}(0)^S < C_i < \tilde{M}_R'^{-1}(0)^L$, as follows:

$$\tilde{M}_R'^{-1}(0)^S < -\frac{\eta}{4} < \tilde{M}_R'^{-1}(0)^L \tag{S298}$$

If $0 < \tilde{M}_R'\left(-\frac{\eta}{4}\right)$, Eq. (S250) leads to

$$-\frac{\eta}{4} < \tilde{M}_R'^{-1}(0)^M \tag{S299}$$

Conversely, if $\tilde{M}_R'\left(-\frac{\eta}{4}\right) < 0$, Eq. (S251) leads to

$$\tilde{M}_R'^{-1}(0)^M < -\frac{\eta}{4} \tag{S300}$$

Furthermore, $\tilde{M}_R'\left(-\frac{\eta}{4}\right) = 0$ means

$$\tilde{M}_R'^{-1}(0)^M = -\frac{\eta}{4} \tag{S301}$$

From Eqs. (S298) to (S301) along with $\tilde{M}_R'^{-1}(0)^S < \tilde{M}_R'^{-1}(0)^M < \tilde{M}_R'^{-1}(0)^L$ under [C1] (Eq. (S248)), we have

$$\tilde{M}_R'^{-1}(0)^S < -\frac{\eta}{4} < \tilde{M}_R'^{-1}(0)^M < \tilde{M}_R'^{-1}(0)^L \quad [\mathrm{C1}, 0 < \tilde{M}_R'\left(-\frac{\eta}{4}\right)] \tag{S302}$$

$$\tilde{M}_R'^{-1}(0)^S < \tilde{M}_R'^{-1}(0)^M < -\frac{\eta}{4} < \tilde{M}_R'^{-1}(0)^L \quad [\mathrm{C1}, \tilde{M}_R'\left(-\frac{\eta}{4}\right) < 0] \tag{S303}$$

$$\tilde{M}_R'^{-1}(0)^S < \tilde{M}_R'^{-1}(0)^M = -\frac{\eta}{4} < \tilde{M}_R'^{-1}(0)^L \quad [\mathrm{C1}, \tilde{M}_R'\left(-\frac{\eta}{4}\right) = 0] \tag{S304}$$

SI 5.4.8 $\tilde{M}_R'''(C_i)$

By differentiating Eq. (S288) with respect to C_i, we have

$$\tilde{M}_R'''(C_i) = 24\tilde{g}_{slp}^2 \left\{C_i + \frac{\eta}{4}\right\} \tag{S305}$$

Eq. (S305) shows that $\tilde{M}_R'''(C_i)$ is a linear function of C_i with a positive slope.

(a) Root: $\tilde{M}_R'''^{-1}(0)$

From Eq. (S305), $\tilde{M}_R'''^{-1}(0)$ is given by

$$\tilde{M}_R'''^{-1}(0) = -\frac{\eta}{4} = \tilde{M}_R''^{-1}(\mathrm{min}) \tag{S306}$$

Note that $\tilde{M}_R'''^{-1}(0)$ is the inflection point of $\tilde{M}_R'(C_i)$ and the minimum point of $\tilde{M}_R''(C_i)$.

Since the slope of $\tilde{M}_R'''(C_i)$ is positive (Eq. (S305)), we have

$$\tilde{M}_R'''(C_i < -\frac{\eta}{4}) < 0 \tag{S307}$$

$$\tilde{M}_R'''(-\frac{\eta}{4} < C_i) > 0 \tag{S308}$$

SI 5.4.9 $\tilde{L}'_R(C_i)$ and $\tilde{M}'_R(C_i)$

We prove several propositions for $\tilde{L}'_R(C_i)$ and $\tilde{M}'_R(C_i)$.

Proposition 2. $\tilde{L}'_R(A_{nR}^{-1}(0) \le C_i \le H_R^{-1}(0)^+) > \tilde{M}'_R(A_{nR}^{-1}(0) \le C_i \le H_R^{-1}(0)^+)$ *under [C1] and* $A_{nR}^{-1}(0) + \frac{\eta}{4} \ge 0$.

Proof. Since $\tilde{M}'^{-1}_R(0)^M \le A_{nR}^{-1}(0) < H_R^{-1}(0)^+$ under the conditions [C1] and $A_{nR}^{-1}(0) + \frac{\eta}{4} \ge 0$ (Eqs. (S256) and (S258)), and $H_R^{-1}(0)^+ < \tilde{M}'^{-1}_R(0)^L$ under [C1] (Eq. (S248)), we obtain

$$\tilde{M}'^{-1}_R(0)^M \le A_{nR}^{-1}(0) < H_R^{-1}(0)^+ < \tilde{M}'^{-1}_R(0)^L. \quad [\mathrm{C1}, A_{nR}^{-1}(0) + \frac{\eta}{4} \ge 0] \tag{S309}$$

Combining Eq. (S309) with $\tilde{M}'_R(\tilde{M}'^{-1}_R(0)^M < C_i < \tilde{M}'^{-1}_R(0)^L) < 0$ (Eq. (S251)) and $\tilde{M}'_R(\tilde{M}'^{-1}_R(0)^M) = 0$ (Eq. (S247)), we obtain

$$\tilde{M}'_R(A_{nR}^{-1}(0) \le C_i \le H_R^{-1}(0)^+) \le 0. \quad [\mathrm{C1}, A_{nR}^{-1}(0) + \frac{\eta}{4} \ge 0] \tag{S310}$$

Since $\tilde{L}'_R(0 < C_i) > 0$ (Eq. (S209)) and $A_{nR}^{-1}(0) > 0$ under [C1] (Table 3), we obtain

$$\tilde{L}'_R(A_{nR}^{-1}(0) \le C_i \le H_R^{-1}(0)^+) > 0. \quad [\mathrm{C1}] \tag{S311}$$

By combining Eqs. (S310) and (S311), we have

$$\tilde{L}'_R(A_{nR}^{-1}(0) \le C_i \le H_R^{-1}(0)^+) > \tilde{M}'_R(A_{nR}^{-1}(0) \le C_i \le H_R^{-1}(0)^+). \quad [\mathrm{C1}, A_{nR}^{-1}(0) + \frac{\eta}{4} \ge 0] \tag{S312}$$

□

Proposition 3. *There is no intersection between* $\tilde{L}'_R(C_i)$ *and* $\tilde{M}'_R(C_i)$ *for* $\tilde{M}'^{-1}_R(0)^M \le C_i \le H_R^{-1}(0)^+$ *under [C1] and* $A_{nR}^{-1}(0) + \frac{\eta}{4} < 0$.

Proof. Eq. (S248) leads to

$$\tilde{M}'^{-1}_R(0)^M < H_R^{-1}(0)^+ < \tilde{M}'^{-1}_R(0)^L. \quad [\mathrm{C1}] \tag{S313}$$

Under [C1] and $A_{nR}^{-1}(0) + \frac{\eta}{4} < 0$, Eqs. (S257) and (S313) leads to

$$A_{nR}^{-1}(0) < \tilde{M}_R'^{-1}(0)^M < H_R^{-1}(0)^+ < \tilde{M}_R'^{-1}(0)^L. \quad [\mathrm{C1}, A_{nR}^{-1}(0) + \frac{\eta}{4} < 0] \tag{S314}$$

By combining $A_{nR}^{-1}(0) > 0$ under [C1] (Table 3) with Eq. (S314), we have

$$0 < \tilde{M}_R'^{-1}(0)^M < H_R^{-1}(0)^+ < \tilde{M}_R'^{-1}(0)^L. \quad [\mathrm{C1}, A_{nR}^{-1}(0) + \frac{\eta}{4} < 0] \tag{S315}$$

Since $\tilde{L}_R'(0 < C_i) > 0$ (Eq. (S209)) and $\tilde{M}_R'(\tilde{M}_R'^{-1}(0)^M \leq C_i \leq \tilde{M}_R'^{-1}(0)^L) \leq 0$ (Eqs. (S247) and (S251)), considering Eq. (S315), we have

$$\tilde{L}_R'(\tilde{M}_R'^{-1}(0)^M \leq C_i \leq H_R^{-1}(0)^+) > \tilde{M}_R'(\tilde{M}_R'^{-1}(0)^M \leq C_i \leq H_R^{-1}(0)^+). \quad [\mathrm{C1}, A_{nR}^{-1}(0) + \frac{\eta}{4} < 0] \tag{S316}$$

Therefore, there is no intersection between $\tilde{L}_R'(C_i)$ and $\tilde{M}_R'(C_i)$ for $\tilde{M}_R'^{-1}(0)^M \leq C_i \leq H_R^{-1}(0)^+$ under [C1] and $A_{nR}^{-1}(0) + \frac{\eta}{4} < 0$. □

Proposition 4. *There is only one intersection between $\tilde{L}_R'(C_i)$ and $\tilde{M}_R'(C_i)$ at $0 < C_i \leq H_R^{-1}(0)^+$ under [C1] and $A_{nR}^{-1}(0) + \frac{\eta}{4} < 0$.*

Proof. Proposition 3 shows that there is no intersection between $\tilde{L}_R'(C_i)$ and $\tilde{M}_R'(C_i)$ in the range $\tilde{M}_R'^{-1}(0)^M \leq C_i \leq H_R^{-1}(0)^+$ under [C1] and $A_{nR}^{-1}(0) + \frac{\eta}{4} < 0$. Therefore, we only need to examine the range $0 < C_i < \tilde{M}_R'^{-1}(0)^M$. Before that, several inequality relations are derived.

First, $A_{nR}^{-1}(0) + \frac{\eta}{4} < 0$, along with $A_{nR}^{-1}(0) > 0$ under [C1] (Table 3), leads to:

$$0 < A_{nR}^{-1}(0) < -\frac{\eta}{4} \quad [\mathrm{C1}, A_{nR}^{-1}(0) + \frac{\eta}{4} < 0] \tag{S317}$$

Second, Proposition 1 shows $\tilde{M}_R'(0) > 0$ under [C1] and $A_{nR}^{-1}(0) + \frac{\eta}{4} < 0$. Meanwhile, Eq. (S204) shows $\tilde{L}_R'(0) = 0$. Thus we have:

$$(0 =)\tilde{L}'_R(0) < \tilde{M}'_R(0) \quad [\text{C1}, A_{nR}^{-1}(0) + \frac{\eta}{4} < 0] \tag{S318}$$

Third, Eq. (S222) shows $\tilde{L}'''_R\left(\frac{A_{nR}^{-1}(*)}{2} < C_i\right) > 0$. Since $A_{nR}^{-1}(*) < 0$ (Eq. (S7)), we have

$$\tilde{L}'''_R(0 \le C_i) > 0 \tag{S319}$$

Eqs. (S306) and (S307) show $\tilde{M}'''_R(C_i \le -\frac{\eta}{4}) \le 0$. Since $0 < -\frac{\eta}{4}$ under [C1] and $A_{nR}^{-1}(0) + \frac{\eta}{4} < 0$ (Eq. (S317)), we have

$$\tilde{M}'''_R(0 \le C_i \le -\frac{\eta}{4}) \le 0 \quad [\text{C1}, A_{nR}^{-1}(0) + \frac{\eta}{4} < 0] \tag{S320}$$

By combining Eqs. (S319) and (S320), we obtain:

$$\tilde{L}'''_R(0 \le C_i \le -\frac{\eta}{4}) > 0 \ge \tilde{M}'''_R(0 \le C_i \le -\frac{\eta}{4}) \quad [\text{C1}, A_{nR}^{-1}(0) + \frac{\eta}{4} < 0] \tag{S321}$$

Fourth, since $\tilde{L}'_R(0 < C_i) > 0$ (Eq. (S209)) and $0 < -\frac{\eta}{4}$ under [C1] and $A_{nR}^{-1}(0) + \frac{\eta}{4} < 0$ (Eq. (S317)), we have:

$$0 < \tilde{L}'_R(-\frac{\eta}{4}) \quad [\text{C1}, A_{nR}^{-1}(0) + \frac{\eta}{4} < 0] \tag{S322}$$

Based on Eq. (S322), we split the analysis into three cases: (A): $0 < \tilde{L}'_R(-\frac{\eta}{4}) \le \tilde{M}'_R(-\frac{\eta}{4})$; (B): $0 < \tilde{M}'_R(-\frac{\eta}{4}) < \tilde{L}'_R(-\frac{\eta}{4})$; and (C): $\tilde{M}'_R(-\frac{\eta}{4}) \le 0 < \tilde{L}'_R(-\frac{\eta}{4})$.

(A): $\tilde{L}'_R(-\frac{\eta}{4}) \le \tilde{M}'_R(-\frac{\eta}{4})$

By combining $0 < \tilde{L}'_R(-\frac{\eta}{4})$ (Eq. (S322)) with $\tilde{L}'_R(-\frac{\eta}{4}) \le \tilde{M}'_R(-\frac{\eta}{4})$, we have

$$0 < \tilde{M}'_R(-\frac{\eta}{4}). \quad [\text{C1}, A^{-1}_{nR}(0) + \frac{\eta}{4} < 0, \tilde{L}'_R(-\frac{\eta}{4}) \leq \tilde{M}'_R(-\frac{\eta}{4})] \tag{S323}$$

In this case, Eq. (S302) shows

$$\tilde{M}'^{-1}_R(0)^S < -\frac{\eta}{4} < \tilde{M}'^{-1}_R(0)^M \quad [\text{C1}, A^{-1}_{nR}(0) + \frac{\eta}{4} < 0, \tilde{L}'_R(-\frac{\eta}{4}) \leq \tilde{M}'_R(-\frac{\eta}{4})] \tag{S324}$$

Combining Eq. (S324) with $0 < -\frac{\eta}{4}$ under [C1] and $A^{-1}_{nR}(0) + \frac{\eta}{4} < 0$ (Eq. (S317)), we obtain

$$0 < -\frac{\eta}{4} < \tilde{M}'^{-1}_R(0)^M \quad [\text{C1}, A^{-1}_{nR}(0) + \frac{\eta}{4} < 0, \tilde{L}'_R(-\frac{\eta}{4}) \leq \tilde{M}'_R(-\frac{\eta}{4})] \tag{S325}$$

Thus, we divide the range $0 < C_i < \tilde{M}'^{-1}_R(0)^M$ from Eq. (S325) into the following two ranges: (i) $0 < C_i < -\frac{\eta}{4}$ and (ii) $-\frac{\eta}{4} \leq C_i < \tilde{M}'^{-1}_R(0)^M$.

(i): $0 < C_i < -\frac{\eta}{4}$

From Eqs. (S318), (S321), and $\tilde{L}'_R(-\frac{\eta}{4}) \leq \tilde{M}'_R(-\frac{\eta}{4})$, which is the condition of case (A), we have:

$$\begin{aligned} \tilde{L}'_R(0) &< \tilde{M}'_R(0) \quad [\text{C1}, A^{-1}_{nR}(0) + \frac{\eta}{4} < 0] && \text{(S326)} \\ \tilde{L}'_R(-\frac{\eta}{4}) &\leq \tilde{M}'_R(-\frac{\eta}{4}) && \text{(S327)} \\ \tilde{L}'''_R(0 \leq C_i \leq -\frac{\eta}{4}) &> \tilde{M}'''_R(0 \leq C_i \leq -\frac{\eta}{4}) \quad [\text{C1}, A^{-1}_{nR}(0) + \frac{\eta}{4} < 0] && \text{(S328)} \end{aligned}$$

Therefore, there is no intersection between $\tilde{L}'_R(C_i)$ and $\tilde{M}'_R(C_i)$ at $0 < C_i < -\frac{\eta}{4}$ under [C1], $A^{-1}_{nR}(0) + \frac{\eta}{4} < 0$, and $\tilde{L}'_R(-\frac{\eta}{4}) \leq \tilde{M}'_R(-\frac{\eta}{4})$.

(ii): $-\frac{\eta}{4} \leq C_i < \tilde{M}'^{-1}_R(0)^M$

Since $\tilde{L}''_R(0 < C_i) > 0$ (Eq. (S217)) and $0 < -\frac{\eta}{4} < \tilde{M}'^{-1}_R(0)^M$ under [C1], $A^{-1}_{nR}(0) + \frac{\eta}{4} < 0$, and

$\tilde{L}'_R(-\frac{\eta}{4}) \leq \tilde{M}'_R(-\frac{\eta}{4})$(Eq. (S325)), we obtain:

$$\tilde{L}''_R(-\frac{\eta}{4} \leq C_i \leq \tilde{M}'^{-1}_R(0)^M) > 0 \quad [\text{C1}, A^{-1}_{nR}(0) + \frac{\eta}{4} < 0, \tilde{L}'_R(-\frac{\eta}{4}) \leq \tilde{M}'_R(-\frac{\eta}{4})] \tag{S329}$$

Meanwhile, $\tilde{M}''_R(\tilde{M}''^{-1}_R(0)^S < C_i < \tilde{M}''^{-1}_R(0)^L) < 0$ (Eq. (S293)) and $\tilde{M}''^{-1}_R(0)^S < -\frac{\eta}{4} < \tilde{M}'^{-1}_R(0)^M < \tilde{M}''^{-1}_R(0)^L$ under [C1], $A^{-1}_{nR}(0) + \frac{\eta}{4} < 0$, and $\tilde{L}'_R(-\frac{\eta}{4}) \leq \tilde{M}'_R(-\frac{\eta}{4})$ (Eqs. (S291), (S295), and S325) lead to:

$$\tilde{M}''_R(-\frac{\eta}{4} \leq C_i \leq \tilde{M}'^{-1}_R(0)^M) < 0 \quad [\text{C1}, A^{-1}_{nR}(0) + \frac{\eta}{4} < 0, \tilde{L}'_R(-\frac{\eta}{4}) \leq \tilde{M}'_R(-\frac{\eta}{4})] \tag{S330}$$

Eqs. (S329) and (S330) show that

$$\begin{gathered}\tilde{L}''_R(-\frac{\eta}{4} \leq C_i \leq \tilde{M}'^{-1}_R(0)^M) > 0 > \tilde{M}''_R(-\frac{\eta}{4} \leq C_i \leq \tilde{M}'^{-1}_R(0)^M) \\ [\text{C1}, A^{-1}_{nR}(0) + \frac{\eta}{4} < 0, \tilde{L}'_R(-\frac{\eta}{4}) \leq \tilde{M}'_R(-\frac{\eta}{4})]\end{gathered} \tag{S331}$$

$\tilde{L}'_R(0 < C_i) > 0$ (Eq. (S209)) and $0 < -\frac{\eta}{4} < \tilde{M}'^{-1}_R(0)^M$ under [C1], $A^{-1}_{nR}(0) + \frac{\eta}{4} < 0$, and $\tilde{L}'_R(-\frac{\eta}{4}) \leq \tilde{M}'_R(-\frac{\eta}{4})$ (Eq. (S325)) lead to:

$$\tilde{L}'_R(\tilde{M}'^{-1}_R(0)^M) > 0 \quad [\text{C1}, A^{-1}_{nR}(0) + \frac{\eta}{4} < 0, \tilde{L}'_R(-\frac{\eta}{4}) \leq \tilde{M}'_R(-\frac{\eta}{4})] \tag{S332}$$

From $\tilde{L}'_R(-\frac{\eta}{4}) \leq \tilde{M}'_R(-\frac{\eta}{4})$, Eqs. (S332) and (S331), we have:

$$\tilde{L}'_R(-\frac{\eta}{4}) \leq \tilde{M}'_R(-\frac{\eta}{4}) \tag{S333}$$

$$\begin{gathered}\tilde{L}'_R(\tilde{M}'^{-1}_R(0)^M) > \tilde{M}'_R(\tilde{M}'^{-1}_R(0)^M) \quad (= 0) \\ [\text{C1}, A^{-1}_{nR}(0) + \frac{\eta}{4} < 0, \tilde{L}'_R(-\frac{\eta}{4}) \leq \tilde{M}'_R(-\frac{\eta}{4})]\end{gathered} \tag{S334}$$

$$\begin{gathered}\tilde{L}''_R(-\frac{\eta}{4} \leq C_i \leq \tilde{M}'^{-1}_R(0)^M) > \tilde{M}''_R(-\frac{\eta}{4} \leq C_i \leq \tilde{M}'^{-1}_R(0)^M) \\ [\text{C1}, A^{-1}_{nR}(0) + \frac{\eta}{4} < 0, \tilde{L}'_R(-\frac{\eta}{4}) \leq \tilde{M}'_R(-\frac{\eta}{4})]\end{gathered} \tag{S335}$$

Therefore, there is one intersection between $\tilde{L}'_R(C_i)$ and $\tilde{M}'_R(C_i)$ in the range $-\frac{\eta}{4} \leq C_i < \tilde{M}'^{-1}_R(0)^M$ under [C1], $A^{-1}_{nR}(0) + \frac{\eta}{4} < 0$, and $\tilde{L}'_R(-\frac{\eta}{4}) \leq \tilde{M}'_R(-\frac{\eta}{4})$.

The results in cases (i) and (ii), along with Proposition 3, show that there are no intersections between $\tilde{L}'_R(C_i)$ and $\tilde{M}'_R(C_i)$ at $0 < C_i < -\frac{\eta}{4}$, one intersection at $-\frac{\eta}{4} \le C_i < \tilde{M}'^{-1}_R(0)^M$, and no intersections at $\tilde{M}'^{-1}_R(0)^M \le C_i \le H_R^{-1}(0)^+$ under [C1], $A_{nR}^{-1}(0) + \frac{\eta}{4} < 0$, and $\tilde{L}'_R(-\frac{\eta}{4}) \le \tilde{M}'_R(-\frac{\eta}{4})$. Thus, there is only one intersection between $\tilde{L}'_R(C_i)$ and $\tilde{M}'_R(C_i)$ in the range $0 < C_i \le H_R^{-1}(0)^+$ under [C1], $A_{nR}^{-1}(0) + \frac{\eta}{4} < 0$, and $\tilde{L}'_R(-\frac{\eta}{4}) \le \tilde{M}'_R(-\frac{\eta}{4})$.

(B): $0 < \tilde{M}'_R(-\frac{\eta}{4}) < \tilde{L}'_R(-\frac{\eta}{4})$

In this case, we have:

$$0 < \tilde{M}'_R(-\frac{\eta}{4}). \tag{S336}$$

By following the same steps to derive Eq. (S325) in case (A), we obtain the same inequality for case (B):

$$0 < -\frac{\eta}{4} < \tilde{M}'^{-1}_R(0)^M. \quad [\mathrm{C1}, A_{nR}^{-1}(0) + \frac{\eta}{4} < 0, 0 < \tilde{M}'_R(-\frac{\eta}{4}) < \tilde{L}'_R(-\frac{\eta}{4})] \tag{S337}$$

Thus, we separate the range into two parts as follows: (i) $0 < C_i < -\frac{\eta}{4}$ and (ii) $-\frac{\eta}{4} \le C_i < \tilde{M}'^{-1}_R(0)^M$.

(i): $0 < C_i < -\frac{\eta}{4}$

From Eqs. (S318) and (S321), along with the condition $\tilde{L}'_R(-\frac{\eta}{4}) > \tilde{M}'_R(-\frac{\eta}{4})$, we obtain:

$$\tilde{L}'_R(0) < \tilde{M}'_R(0) \quad [\mathrm{C1}, A_{nR}^{-1}(0) + \frac{\eta}{4} < 0] \tag{S338}$$

$$\tilde{L}'_R(-\frac{\eta}{4}) > \tilde{M}'_R(-\frac{\eta}{4}) \tag{S339}$$

$$\tilde{L}'''_R(0 \le C_i \le -\frac{\eta}{4}) > \tilde{M}'''_R(0 \le C_i \le -\frac{\eta}{4}) \quad [\mathrm{C1}, A_{nR}^{-1}(0) + \frac{\eta}{4} < 0]. \tag{S340}$$

These confirm that there is exactly one intersection between $\tilde{L}'_R(C_i)$ and $\tilde{M}'_R(C_i)$ in the range

$0 < C_i < -\frac{\eta}{4}$ under the conditions [C1], $A_{nR}^{-1}(0) + \frac{\eta}{4} < 0$, and $0 < \tilde{M}'_R(-\frac{\eta}{4}) < \tilde{L}'_R(-\frac{\eta}{4})$.

(ii): $-\frac{\eta}{4} \le C_i < \tilde{M}'^{-1}_R(0)^M$

Since $\tilde{L}'_R(0 < C_i) > 0$ (Eq. (S209)) and $0 < -\frac{\eta}{4} < \tilde{M}'^{-1}_R(0)^M$ (Eq. (S337)), we find:

$$\tilde{L}'_R(\tilde{M}'^{-1}_R(0)^M) > 0. \quad [\mathrm{C1}, A_{nR}^{-1}(0) + \frac{\eta}{4} < 0, 0 < \tilde{M}'_R(-\frac{\eta}{4}) < \tilde{L}'_R(-\frac{\eta}{4})] \tag{S341}$$

Following the same process as in (A) (Eqs. (S329) to (S331)), we find:

$$\begin{aligned} &\tilde{L}''_R(-\frac{\eta}{4} \le C_i \le \tilde{M}'^{-1}_R(0)^M) > 0 > \tilde{M}''_R(-\frac{\eta}{4} \le C_i \le \tilde{M}'^{-1}_R(0)^M). \\ &[\mathrm{C1}, A_{nR}^{-1}(0) + \frac{\eta}{4} < 0, 0 < \tilde{M}'_R(-\frac{\eta}{4}) < \tilde{L}'_R(-\frac{\eta}{4})] \end{aligned} \tag{S342}$$

From the conditions of (B), Eqs. (S341) and (S342), we find:

$$\tilde{L}'_R(-\frac{\eta}{4}) > \tilde{M}'_R(-\frac{\eta}{4}) \tag{S343}$$

$$\begin{aligned} &\tilde{L}'_R(\tilde{M}'^{-1}_R(0)^M) > \tilde{M}'_R(\tilde{M}'^{-1}_R(0)^M) \quad (= 0) \\ &[\mathrm{C1}, A_{nR}^{-1}(0) + \frac{\eta}{4} < 0, 0 < \tilde{M}'_R(-\frac{\eta}{4}) < \tilde{L}'_R(-\frac{\eta}{4})] \end{aligned} \tag{S344}$$

$$\begin{aligned} &\tilde{L}''_R(-\frac{\eta}{4} \le C_i \le \tilde{M}'^{-1}_R(0)^M) > \tilde{M}''_R(-\frac{\eta}{4} \le C_i \le \tilde{M}'^{-1}_R(0)^M) \\ &[\mathrm{C1}, A_{nR}^{-1}(0) + \frac{\eta}{4} < 0, 0 < \tilde{M}'_R(-\frac{\eta}{4}) < \tilde{L}'_R(-\frac{\eta}{4})]. \end{aligned} \tag{S345}$$

These indicate that there is no intersection between $\tilde{L}'_R(C_i)$ and $\tilde{M}'_R(C_i)$ in the range $-\frac{\eta}{4} \le C_i < \tilde{M}'^{-1}_R(0)^M$ under [C1], $A_{nR}^{-1}(0) + \frac{\eta}{4} < 0$, and $0 < \tilde{M}'_R(-\frac{\eta}{4}) < \tilde{L}'_R(-\frac{\eta}{4})$.

Thus, under the conditions [C1], $A_{nR}^{-1}(0) + \frac{\eta}{4} < 0$, and $0 < \tilde{M}'_R(-\frac{\eta}{4}) < \tilde{L}'_R(-\frac{\eta}{4})$, there is only one intersection between $\tilde{L}'_R(C_i)$ and $\tilde{M}'_R(C_i)$ in the range $0 < C_i \le H_R^{-1}(0)^+$.

(C): $\tilde{M}'_R(-\frac{\eta}{4}) \le 0 < \tilde{L}'_R(-\frac{\eta}{4})$

In this case, Eqs. (S303) and (S304) show that $\tilde{M}'_R(-\frac{\eta}{4}) \le 0$ implies:

$$\tilde{M}'^{-1}_R(0)^M \le -\frac{\eta}{4}. \quad [\mathrm{C1}, \tilde{M}'_R(-\frac{\eta}{4}) \le 0] \tag{S346}$$

Under $A_{nR}^{-1}(0) + \frac{\eta}{4} < 0$, Eq. (S257) shows:

$$A_{nR}^{-1}(0) < \tilde{M}_R'^{-1}(0)^M. \quad [\text{C1}, A_{nR}^{-1}(0) + \frac{\eta}{4} < 0] \tag{S347}$$

By combining Eqs. (S346) and (S347) with $A_{nR}^{-1}(0) > 0$ under [C1] (Table 3), we obtain:

$$0 < A_{nR}^{-1}(0) < \tilde{M}_R'^{-1}(0)^M \leq -\frac{\eta}{4} \quad [\text{C1}, A_{nR}^{-1}(0) + \frac{\eta}{4} < 0, \tilde{M}_R'(-\frac{\eta}{4}) \leq 0] \tag{S348}$$

From $\tilde{L}_R'(0 < C_i) > 0$ (Eq. (S209)) and Eq. (S348), we have:

$$\tilde{L}_R'(\tilde{M}_R'^{-1}(0)^M) > 0. \quad [\text{C1}, A_{nR}^{-1}(0) + \frac{\eta}{4} < 0, \tilde{M}_R'(-\frac{\eta}{4}) \leq 0] \tag{S349}$$

For the range $0 \leq C_i \leq \tilde{M}_R'^{-1}(0)^M$, from Eqs. (S318), (S349), and (S321), we find:

$$\tilde{L}_R'(0) < \tilde{M}_R'(0) \quad [\text{C1}, A_{nR}^{-1}(0) + \frac{\eta}{4} < 0] \tag{S350}$$

$$\begin{gathered}\tilde{L}_R'(\tilde{M}_R'^{-1}(0)^M) > \tilde{M}_R'(\tilde{M}_R'^{-1}(0)^M) \quad (= 0) \\ [\text{C1}, A_{nR}^{-1}(0) + \frac{\eta}{4} < 0, \tilde{M}_R'(-\frac{\eta}{4}) \leq 0]\end{gathered} \tag{S351}$$

$$\begin{gathered}\tilde{L}_R'''(0 \leq C_i \leq \tilde{M}_R'^{-1}(0)^M) > \tilde{M}_R'''(0 \leq C_i \leq \tilde{M}_R'^{-1}(0)^M). \\ [\text{C1}, A_{nR}^{-1}(0) + \frac{\eta}{4} < 0]\end{gathered} \tag{S352}$$

These show that there is only one intersection between $\tilde{L}_R'(C_i)$ and $\tilde{M}_R'(C_i)$ in the range $0 < C_i < \tilde{M}_R'^{-1}(0)^M$ under [C1], $A_{nR}^{-1}(0) + \frac{\eta}{4} < 0$, and $\tilde{M}_R'(-\frac{\eta}{4}) \leq 0 < \tilde{L}_R'(-\frac{\eta}{4})$.

Proposition 3 further confirms that there are no intersections in the range $\tilde{M}_R'^{-1}(0)^M \leq C_i \leq H_R^{-1}(0)^+$ under [C1] and $A_{nR}^{-1}(0) + \frac{\eta}{4} < 0$. Thus, there is exactly one intersection between $\tilde{L}_R'(C_i)$ and $\tilde{M}_R'(C_i)$ in the range $0 < C_i \leq H_R^{-1}(0)^+$ under [C1] and $A_{nR}^{-1}(0) + \frac{\eta}{4} < 0$, and $\tilde{M}_R'(-\frac{\eta}{4}) \leq 0 < \tilde{L}_R'(-\frac{\eta}{4})$.

This completes the proof that there is exactly one intersection between $\tilde{L}_R'(C_i)$ and $\tilde{M}_R'(C_i)$ in the range $0 < C_i \leq H_R^{-1}(0)^+$ under [C1] and $A_{nR}^{-1}(0) + \frac{\eta}{4} < 0$. □

Proposition 5. $\tilde{L}'_R(A_{nR}^{-1}(0) \le C_i \le H_R^{-1}(0)^+) > \tilde{M}'_R(A_{nR}^{-1}(0) \le C_i \le H_R^{-1}(0)^+)$ *under [C1],* $A_{nR}^{-1}(0) + \frac{\eta}{4} < 0$*, and* $\tilde{L}'_R(A_{nR}^{-1}(0)) > \tilde{M}'_R(A_{nR}^{-1}(0))$.

Proof. In the case of [C1] and $A_{nR}^{-1}(0) + \frac{\eta}{4} < 0$, Eq. (S257) leads to

$$A_{nR}^{-1}(0) < H_R^{-1}(0)^+. \quad [\text{C1}, A_{nR}^{-1}(0) + \frac{\eta}{4} < 0] \tag{S353}$$

Combining Eq. (S353) with $A_{nR}^{-1}(0) > 0$ under [C1] (Table 3), we have:

$$0 < A_{nR}^{-1}(0) < H_R^{-1}(0)^+. \quad [\text{C1}, A_{nR}^{-1}(0) + \frac{\eta}{4} < 0] \tag{S354}$$

$\tilde{L}'_R(0) < \tilde{M}'_R(0)$ under [C1] and $A_{nR}^{-1}(0) + \frac{\eta}{4} < 0$ (Eq. (S318)) and $\tilde{L}'_R(A_{nR}^{-1}(0)) > \tilde{M}'_R(A_{nR}^{-1}(0))$, which is a condition of this proposition, lead to:

$$\tilde{L}'_R(0) \quad < \quad \tilde{M}'_R(0) \quad [\text{C1}, A_{nR}^{-1}(0) + \frac{\eta}{4} < 0] \tag{S355}$$

$$\tilde{L}'_R(A_{nR}^{-1}(0)) \quad > \quad \tilde{M}'_R(A_{nR}^{-1}(0)) \tag{S356}$$

Eqs. (S355) and (S356) indicate that there is at least one intersection between $\tilde{L}'_R(C_i)$ and $\tilde{M}'_R(C_i)$ in the range $0 < C_i < A_{nR}^{-1}(0)$. Considering Proposition 4 together with Eqs. (S355) and (S356), we conclude that there is exactly one intersection between $\tilde{L}'_R(C_i)$ and $\tilde{M}'_R(C_i)$ in the range $0 < C_i < A_{nR}^{-1}(0)$, and no intersections in the range $A_{nR}^{-1}(0) \le C_i \le H_R^{-1}(0)^+$. Therefore, we have

$$\begin{gathered}\tilde{L}'_R(A_{nR}^{-1}(0) \le C_i \le H_R^{-1}(0)^+) > \tilde{M}'_R(A_{nR}^{-1}(0) \le C_i \le H_R^{-1}(0)^+). \\ [\text{C1}, A_{nR}^{-1}(0) + \frac{\eta}{4} < 0, \tilde{L}'_R(A_{nR}^{-1}(0)) > \tilde{M}'_R(A_{nR}^{-1}(0))]\end{gathered} \tag{S357}$$

□

Proposition 6. $0 < \tilde{M}'_R(A_{nR}^{-1}(0)) < \tilde{L}'_R(A_{nR}^{-1}(0))$ *under [C1],* $A_{nR}^{-1}(0) + \frac{\eta}{4} < 0$*, and* $\tilde{L}_R(A_{nR}^{-1}(0)) \ge \tilde{M}_R(A_{nR}^{-1}(0))$.

Proof. Eq. (S255) shows that $0 < \tilde{M}'_R(A_{nR}^{-1}(0))$ under [C1] and $A_{nR}^{-1}(0) + \frac{\eta}{4} < 0$. Therefore, what remains to be proven is that $\tilde{M}'_R(A_{nR}^{-1}(0)) < \tilde{L}'_R(A_{nR}^{-1}(0))$.

Using Eqs. (S201) and (S236), we can express the inequality $\tilde{M}_R(A_{nR}^{-1}(0)) \leq \tilde{L}_R(A_{nR}^{-1}(0))$, which is a condition of this proposition, as follows:

$$\tilde{M}_R(A_{nR}^{-1}(0)) \leq \tilde{L}_R(A_{nR}^{-1}(0)) \tag{S358}$$

$$\Longleftrightarrow \tilde{g}_{slp}^2\{A_{nR}^{-1}(0) - C_a\}^2\{A_{nR}^{-1}(0) - A_{nR}^{-1}(*)\}^2 \leq \{A_{nR}^{-1}(0)\}^2\{A_{nR}^{-1}(0) - A_{nR}^{-1}(*)\}^2 \tag{S359}$$

$$\Longleftrightarrow \tilde{g}_{slp}^2\{A_{nR}^{-1}(0) - C_a\}^2 \leq \{A_{nR}^{-1}(0)\}^2 \tag{S360}$$

$$\Longleftrightarrow \left\{\tilde{g}_{slp}\{A_{nR}^{-1}(0) - C_a\} - A_{nR}^{-1}(0)\right\}\left\{\tilde{g}_{slp}\{A_{nR}^{-1}(0) - C_a\} + A_{nR}^{-1}(0)\right\} \leq 0 \tag{S361}$$

Since $0 < A_{nR}^{-1}(0) < C_a$ under [C1] (Table 3), the first term of the product on the left-hand side of Eq. (S361) is negative, as follows:

$$\tilde{g}_{slp}\{A_{nR}^{-1}(0) - C_a\} - A_{nR}^{-1}(0) < 0 \quad \text{[C1]} \tag{S362}$$

From Eqs. (S361) and (S362), we have:

$$\tilde{g}_{slp}\{A_{nR}^{-1}(0) - C_a\} + A_{nR}^{-1}(0) \geq 0 \quad [\text{C1}, \tilde{M}_R(A_{nR}^{-1}(0)) \leq \tilde{L}_R(A_{nR}^{-1}(0))] \tag{S363}$$

$$\Longleftrightarrow \tilde{g}_{slp}C_a \leq \{1 + \tilde{g}_{slp}\}A_{nR}^{-1}(0) \quad [\text{C1}, \tilde{M}_R(A_{nR}^{-1}(0)) \leq \tilde{L}_R(A_{nR}^{-1}(0))] \tag{S364}$$

$$\Longleftrightarrow C_a \leq A_{nR}^{-1}(0) + \frac{A_{nR}^{-1}(0)}{\tilde{g}_{slp}} \quad [\text{C1}, \tilde{M}_R(A_{nR}^{-1}(0)) \leq \tilde{L}_R(A_{nR}^{-1}(0))] \tag{S365}$$

Eq. (S365) provides the upper limit of C_a in the case where [C1] and $\tilde{M}_R(A_{nR}^{-1}(0)) \leq \tilde{L}_R(A_{nR}^{-1}(0))$.

Next, we derive a similar inequality for C_a from $\tilde{L}'_R(A_{nR}^{-1}(0)) > \tilde{M}'_R(A_{nR}^{-1}(0))$. Using Eqs. (S210) and (S253), we rearrange the inequality $\tilde{L}'_R(A_{nR}^{-1}(0)) > \tilde{M}'_R(A_{nR}^{-1}(0))$, as follows:

$$\tilde{L}'_R(A_{nR}^{-1}(0)) > \tilde{M}'_R(A_{nR}^{-1}(0)) \tag{S366}$$

$$\begin{aligned} &\Longleftrightarrow 4A_{nR}^{-1}(0)\{A_{nR}^{-1}(0) - A_{nR}^{-1}(*)\}\{A_{nR}^{-1}(0) - \frac{A_{nR}^{-1}(*)}{2}\} \\ &\quad > 4\tilde{g}_{slp}^2\{A_{nR}^{-1}(0) - C_a\}\{A_{nR}^{-1}(0) - A_{nR}^{-1}(*)\}\{A_{nR}^{-1}(0) + \frac{\eta}{4}\} \end{aligned} \tag{S367}$$

$$\iff C_a < A_{nR}^{-1}(0) - \frac{A_{nR}^{-1}(0)\{A_{nR}^{-1}(0) - \frac{A_{nR}^{-1}(*)}{2}\}}{\tilde{g}_{slp}^2\{A_{nR}^{-1}(0) + \frac{\eta}{4}\}} \quad [A_{nR}^{-1}(0) + \frac{\eta}{4} < 0] \tag{S368}$$

$$\iff C_a < A_{nR}^{-1}(0) + \frac{A_{nR}^{-1}(0)}{\tilde{g}_{slp}} \left\{ -\frac{A_{nR}^{-1}(0) - \frac{A_{nR}^{-1}(*)}{2}}{\tilde{g}_{slp}\{A_{nR}^{-1}(0) + \frac{\eta}{4}\}} \right\} \quad [A_{nR}^{-1}(0) + \frac{\eta}{4} < 0] \tag{S369}$$

Note that $A_{nR}^{-1}(0) + \frac{\eta}{4} < 0$ is assumed to obtain Eqs. (S368) and (S369). Thus, $\tilde{L}'_R(A_{nR}^{-1}(0)) > \tilde{M}'_R(A_{nR}^{-1}(0))$ holds under $A_{nR}^{-1}(0) + \frac{\eta}{4} < 0$ if Eq. (S369) holds under $A_{nR}^{-1}(0) + \frac{\eta}{4} < 0$. By comparing the terms on the right-hand side of Eqs. (S365) and (S369), we assume the following inequality:

$$A_{nR}^{-1}(0) + \frac{A_{nR}^{-1}(0)}{\tilde{g}_{slp}} < A_{nR}^{-1}(0) + \frac{A_{nR}^{-1}(0)}{\tilde{g}_{slp}} \left\{ -\frac{A_{nR}^{-1}(0) - \frac{A_{nR}^{-1}(*)}{2}}{\tilde{g}_{slp}\{A_{nR}^{-1}(0) + \frac{\eta}{4}\}} \right\} \tag{S370}$$

Eq. (S370) holds under [C1] where $A_{nR}^{-1}(0) > 0$ if the following inequality holds:

$$-\frac{A_{nR}^{-1}(0) - \frac{A_{nR}^{-1}(*)}{2}}{\tilde{g}_{slp}\{A_{nR}^{-1}(0) + \frac{\eta}{4}\}} > 1 \tag{S371}$$

Rearranging Eq. (S371), we obtain:

$$-\frac{A_{nR}^{-1}(0) - \frac{A_{nR}^{-1}(*)}{2}}{\tilde{g}_{slp}\{A_{nR}^{-1}(0) + \frac{\eta}{4}\}} > 1 \tag{S372}$$

$$\iff A_{nR}^{-1}(0) - \frac{A_{nR}^{-1}(*)}{2} > -\tilde{g}_{slp} \left\{ A_{nR}^{-1}(0) + \frac{-1}{2} \left\{ C_a + A_{nR}^{-1}(*) - \left\{ \lambda_b - \frac{\lambda_s}{2} \right\} \frac{A_{nR}(\pm\infty)}{g_b} \right\} \right\} \quad [A_{nR}^{-1}(0) + \frac{\eta}{4} < 0] \tag{S373}$$

$$\iff \tilde{g}_{slp} C_a < 2\{1 + \tilde{g}_{slp}\}A_{nR}^{-1}(0) - \{1 + \tilde{g}_{slp}\}A_{nR}^{-1}(*) + \tilde{g}_{slp} \left\{ \lambda_b - \frac{\lambda_s}{2} \right\} \frac{A_{nR}(\pm\infty)}{g_b} \quad [A_{nR}^{-1}(0) + \frac{\eta}{4} < 0] \tag{S374}$$

Note that $A_{nR}^{-1}(0) + \frac{\eta}{4} < 0$ is assumed to derive Eq. (S373).

Considering that $A_{nR}^{-1}(0) > 0$, $A_{nR}^{-1}(*) < 0$, and $A_{nR}(\pm\infty) > 0$ under [C1] (Table 3), we can derive the following valid inequality:

$$\{1 + \tilde{g}_{slp}\}A_{nR}^{-1}(0) < 2\{1 + \tilde{g}_{slp}\}A_{nR}^{-1}(0) - \{1 + \tilde{g}_{slp}\}A_{nR}^{-1}(*) + \tilde{g}_{slp} \left(\lambda_b - \frac{\lambda_s}{2} \right) \frac{A_{nR}(\pm\infty)}{g_b} \quad [C1] \tag{S375}$$

Eq. (S375) confirms that Eq. (S374) holds if Eq. (S364) is valid. Indeed, Eq. (S364) is valid as it is derived from $\tilde{L}_R(A_{nR}^{-1}(0)) \geq \tilde{M}_R(A_{nR}^{-1}(0))$, which is one of the conditions for the proposition. Therefore, Eq. (S374) is valid under [C1], $A_{nR}^{-1}(0) + \frac{\eta}{4} < 0$, and $\tilde{L}_R(A_{nR}^{-1}(0)) \geq \tilde{M}_R(A_{nR}^{-1}(0))$.

The validity of Eq. (S374) implies that Eq. (S371) also holds under $[A_{nR}^{-1}(0)+\frac{\eta}{4} < 0]$(Eqs. (S371) to (S374)). Thus we have:

$$-\frac{A_{nR}^{-1}(0) - \frac{A_{nR}^{-1}(*)}{2}}{\tilde{g}_{slp}\{A_{nR}^{-1}(0) + \frac{\eta}{4}\}} > 1 \quad [\mathrm{C1}, A_{nR}^{-1}(0) + \frac{\eta}{4} < 0, \tilde{L}_R(A_{nR}^{-1}(0)) \geq \tilde{M}_R(A_{nR}^{-1}(0))]. \tag{S376}$$

Considering Eq. (S376), it follows that Eq. (S370) is valid. Combining Eq. (S370) with Eq. (S365), we obtain:

$$\begin{aligned} C_a \leq A_{nR}^{-1}(0) + \frac{A_{nR}^{-1}(0)}{\tilde{g}_{slp}} < A_{nR}^{-1}(0) + \frac{A_{nR}^{-1}(0)}{\tilde{g}_{slp}} \left\{ -\frac{A_{nR}^{-1}(0) - \frac{A_{nR}^{-1}(*)}{2}}{\tilde{g}_{slp}\{A_{nR}^{-1}(0) + \frac{\eta}{4}\}} \right\} \\ [\mathrm{C1}, A_{nR}^{-1}(0) + \frac{\eta}{4} < 0, \tilde{L}_R(A_{nR}^{-1}(0)) \geq \tilde{M}_R(A_{nR}^{-1}(0))]. \end{aligned} \tag{S377}$$

Eq. (S377) demonstrates that Eq. (S369), which is equivalent to Eq. (S366), is valid under [C1], $A_{nR}^{-1}(0) + \frac{\eta}{4} < 0$, and $\tilde{L}_R(A_{nR}^{-1}(0)) \geq \tilde{M}_R(A_{nR}^{-1}(0))$. Therefore, Proposition 6 is proven. □

SI 5.4.10 $\tilde{N}_R(C_i) = \tilde{L}_R(C_i) - \tilde{M}_R(C_i) = 0$

The roots of $\tilde{N}_R(C_i) = 0$, denoted as $\tilde{N}_R^{-1}(0)$, represent the intersections between $\tilde{L}_R(C_i)$ and $\tilde{M}_R(C_i)$ (Eq. (S195)). To analyze these intersections, we categorize the geometric patterns of $\tilde{L}_R(C_i)$ and $\tilde{M}_R(C_i)$ into four cases based on the relationships between $\tilde{L}_R(A_{nR}^{-1}(0))$ and $\tilde{M}_R(A_{nR}^{-1}(0))$, as well as their derivatives. These cases are labeled [P1] through [P4], and Table S41 provides the specific conditions for each pattern.

Table S41: Four patterns for $\tilde{L}_R(C_i)$ and $\tilde{M}_R(C_i)$

Case	$\tilde{L}_R(A_{nR}^{-1}(0))$ and $\tilde{M}_R(A_{nR}^{-1}(0))$	$\tilde{L}'_R(A_{nR}^{-1}(0))$ and $\tilde{M}'_R(A_{nR}^{-1}(0))$
P1	$\tilde{L}_R(A_{nR}^{-1}(0)) < \tilde{M}_R(A_{nR}^{-1}(0))$	$\tilde{M}'_R(A_{nR}^{-1}(0)) \leq 0 < \tilde{L}'_R(A_{nR}^{-1}(0))$
P2	$\tilde{L}_R(A_{nR}^{-1}(0)) \geq \tilde{M}_R(A_{nR}^{-1}(0))$	$\tilde{M}'_R(A_{nR}^{-1}(0)) \leq 0 < \tilde{L}'_R(A_{nR}^{-1}(0))$
P3	$\tilde{L}_R(A_{nR}^{-1}(0)) < \tilde{M}_R(A_{nR}^{-1}(0))$	$0 < \tilde{M}'_R(A_{nR}^{-1}(0))$
P4	$\tilde{L}_R(A_{nR}^{-1}(0)) \geq \tilde{M}_R(A_{nR}^{-1}(0))$	$0 < \tilde{M}'_R(A_{nR}^{-1}(0))(< \tilde{L}'_R(A_{nR}^{-1}(0)))$

It is important to note that Proposition 6 shows that $\tilde{M}'_R(A_{nR}^{-1}(0)) < \tilde{L}'_R(A_{nR}^{-1}(0))$ when $\tilde{L}_R(A_{nR}^{-1}(0)) \geq \tilde{M}_R(A_{nR}^{-1}(0))$ and $0 < \tilde{M}'_R(A_{nR}^{-1}(0))$ under [C1] (Eq. (S255)). Additionally, we focus on the range $A_{nR}^{-1}(0) < C_i < H_R^{-1}(0)^+$, which is the possible range of appropriate solutions under [C1] (Table 4). We note that $A_{nR}^{-1}(0) = F_R^{-1}(0)$ and $H_R^{-1}(0)^+ = F_R^{-1}(*)^+$ (Eqs. (S93) and (S87)).

(P1) $\tilde{L}_R(A_{nR}^{-1}(0)) < \tilde{M}_R(A_{nR}^{-1}(0))$ and $\tilde{M}'_R(A_{nR}^{-1}(0)) \leq 0 < \tilde{L}'_R(A_{nR}^{-1}(0))$

Eq. (S255) shows that $A_{nR}^{-1}(0) + \frac{\eta}{4} \geq 0$ holds when $\tilde{M}'_R(A_{nR}^{-1}(0)) \leq 0$ under [C1]. Thus, we assume $A_{nR}^{-1}(0) + \frac{\eta}{4} \geq 0$ in [P1].

Given $\tilde{L}_R(A_{nR}^{-1}(0)) < \tilde{M}_R(A_{nR}^{-1}(0))$, which is one of the conditions in [P1], along with $\tilde{L}_R(H_R^{-1}(0)^+) > 0$ (Eq. (S202)), $\tilde{M}_R(H_R^{-1}(0)^+) = 0$ (Eq. (S232)), and Proposition 2, we obtain:

$$\tilde{L}_R(A_{nR}^{-1}(0)) < \tilde{M}_R(A_{nR}^{-1}(0)) \tag{S378}$$

$$\tilde{L}_R(H_R^{-1}(0)^+) > \tilde{M}_R(H_R^{-1}(0)^+) = 0 \tag{S379}$$

$$\begin{aligned} \tilde{L}'_R(A_{nR}^{-1}(0) \leq C_i \leq H_R^{-1}(0)^+) > \tilde{M}'_R(A_{nR}^{-1}(0) \leq C_i \leq H_R^{-1}(0)^+) \\ [\text{C1}, A_{nR}^{-1}(0) + \frac{\eta}{4} \geq 0] \end{aligned} \tag{S380}$$

Therefore, there is only one solution between $\tilde{L}_R(C_i)$ and $\tilde{M}_R(C_i)$ in the range $A_{nR}^{-1}(0) < C_i < H_R^{-1}(0)^+$. Thus, we have:

$$\begin{aligned} \tilde{L}_R(A_{nR}^{-1}(0) < C_i < \tilde{N}_R^{-1}(0)) < \tilde{M}_R(A_{nR}^{-1}(0) < C_i < \tilde{N}_R^{-1}(0)) \\ \tilde{L}_R(\tilde{N}_R^{-1}(0)) = \tilde{M}_R(\tilde{N}_R^{-1}(0)) \\ \tilde{L}_R(\tilde{N}_R^{-1}(0) < C_i < H_R^{-1}(0)^+) > \tilde{M}_R(\tilde{N}_R^{-1}(0) < C_i < H_R^{-1}(0)^+) \\ [\text{C1}, A_{nR}^{-1}(0) + \frac{\eta}{4} \geq 0, \tilde{L}_R(A_{nR}^{-1}(0)) < \tilde{M}_R(A_{nR}^{-1}(0))] \end{aligned} \tag{S381}$$

Hence,

$$\begin{aligned} \tilde{N}_R(A_{nR}^{-1}(0) < C_i < \tilde{N}_R^{-1}(0)) < 0 \\ \tilde{N}_R(\tilde{N}_R^{-1}(0)) = 0 \\ \tilde{N}_R(\tilde{N}_R^{-1}(0) < C_i < H_R^{-1}(0)^+) > 0 \\ [\text{C1}, A_{nR}^{-1}(0) + \frac{\eta}{4} \geq 0, \tilde{L}_R(A_{nR}^{-1}(0)) < \tilde{M}_R(A_{nR}^{-1}(0))] \end{aligned} \tag{S382}$$

Figures S19 and S20 illustrate the curves of $\tilde{L}_R(C_i)$ and $\tilde{M}_R(C_i)$, as well as $\tilde{N}_R(C_i)$ for case [P1].

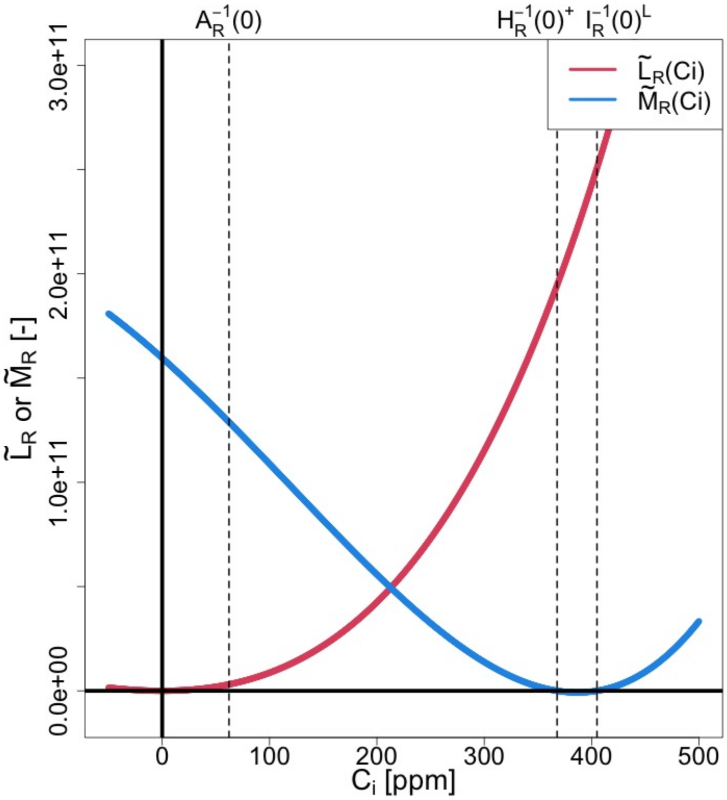

Figure S19: $\tilde{L}_R(C_i)$ and $\tilde{M}_R(C_i)$ for [P1]

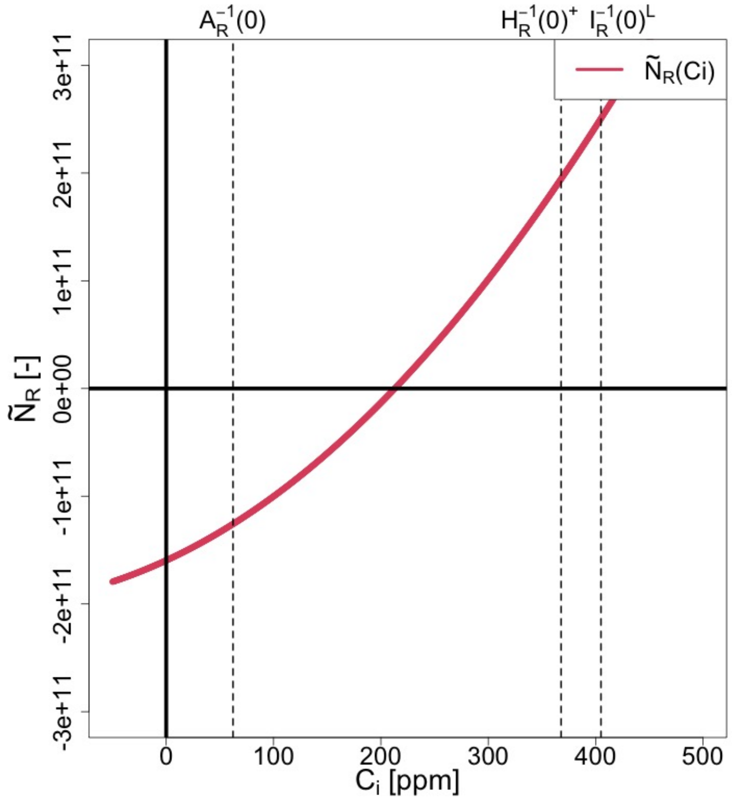

Figure S20: $\tilde{N}_R(C_i)$ for [P1]

(P2) $\tilde{L}_R(A_{nR}^{-1}(0)) \geq \tilde{M}_R(A_{nR}^{-1}(0))$ and $\tilde{M}'_R(A_{nR}^{-1}(0)) \leq 0 < \tilde{L}'_R(A_{nR}^{-1}(0))$

Similar to the case in [P1], we can assume $A_{nR}^{-1}(0) + \frac{\eta}{4} \geq 0$ from $\tilde{M}'_R(A_{nR}^{-1}(0)) \leq 0$ (Eq. (S255)).

Given $\tilde{L}_R(A_{nR}^{-1}(0)) \geq \tilde{M}_R(A_{nR}^{-1}(0))$, which is one of the conditions in (P2), along with $\tilde{L}_R(H_R^{-1}(0)^+) > 0$ (Eq. (S202)), $\tilde{M}_R(H_R^{-1}(0)^+) = 0$ (Eq. (S232)), and Proposition 2, we obtain:

$$\tilde{L}_R(A_{nR}^{-1}(0)) \geq \tilde{M}_R(A_{nR}^{-1}(0)) \quad \text{(S383)}$$

$$\tilde{L}_R(H_R^{-1}(0)^+) > \tilde{M}_R(H_R^{-1}(0)^+) = 0 \quad \text{(S384)}$$

$$\tilde{L}'_R(A_{nR}^{-1}(0) \leq C_i \leq H_R^{-1}(0)^+) > \tilde{M}'_R(A_{nR}^{-1}(0) \leq C_i \leq H_R^{-1}(0)^+) \quad [\text{C1}, A_{nR}^{-1}(0) + \frac{\eta}{4} \geq 0] \quad \text{(S385)}$$

Therefore, there is no solution between $\tilde{L}_R(C_i)$ and $\tilde{M}_R(C_i)$ in the range $A_{nR}^{-1}(0) < C_i <$

$H_R^{-1}(0)^+$. Thus, we have:

$$\tilde{L}_R(A_{nR}^{-1}(0) < C_i < H_R^{-1}(0)^+) > \tilde{M}_R(A_{nR}^{-1}(0) < C_i < H_R^{-1}(0)^+)$$
$$[\text{C1}, A_{nR}^{-1}(0) + \frac{\eta}{4} \geq 0, \tilde{L}_R(A_{nR}^{-1}(0)) \geq \tilde{M}_R(A_{nR}^{-1}(0))] \quad \text{(S386)}$$

Hence,

$$\tilde{N}_R(A_{nR}^{-1}(0) < C_i < H_R^{-1}(0)^+) > 0$$
$$[\text{C1}, A_{nR}^{-1}(0) + \frac{\eta}{4} \geq 0, \tilde{L}_R(A_{nR}^{-1}(0)) \geq \tilde{M}_R(A_{nR}^{-1}(0))] \quad \text{(S387)}$$

Figures S21 and S22 illustrate the curves of $\tilde{L}_R(C_i)$ and $\tilde{M}_R(C_i)$, as well as $\tilde{N}_R(C_i)$ for case [P2].

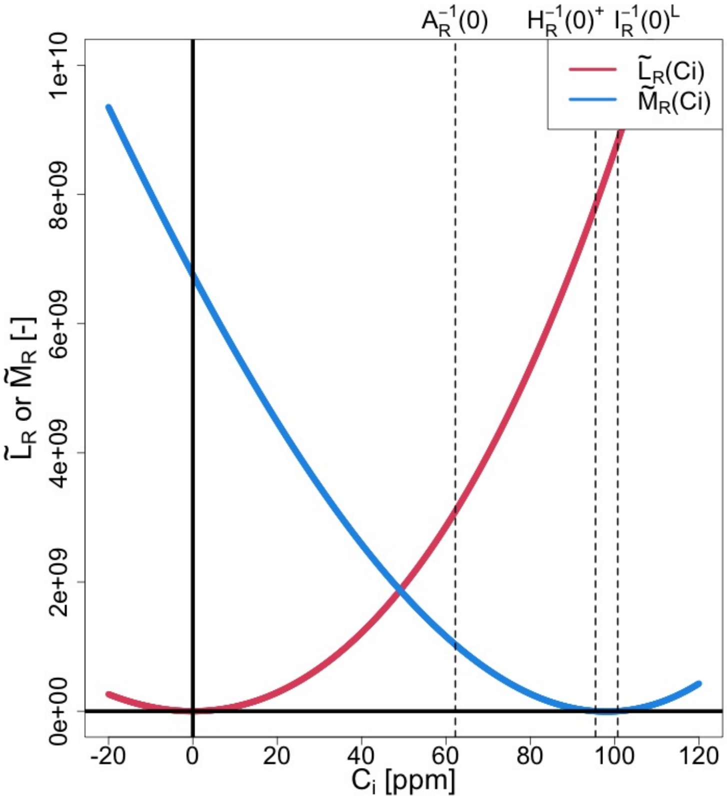

Figure S21: $\tilde{L}_R(C_i)$ and $\tilde{M}_R(C_i)$ for [P2]

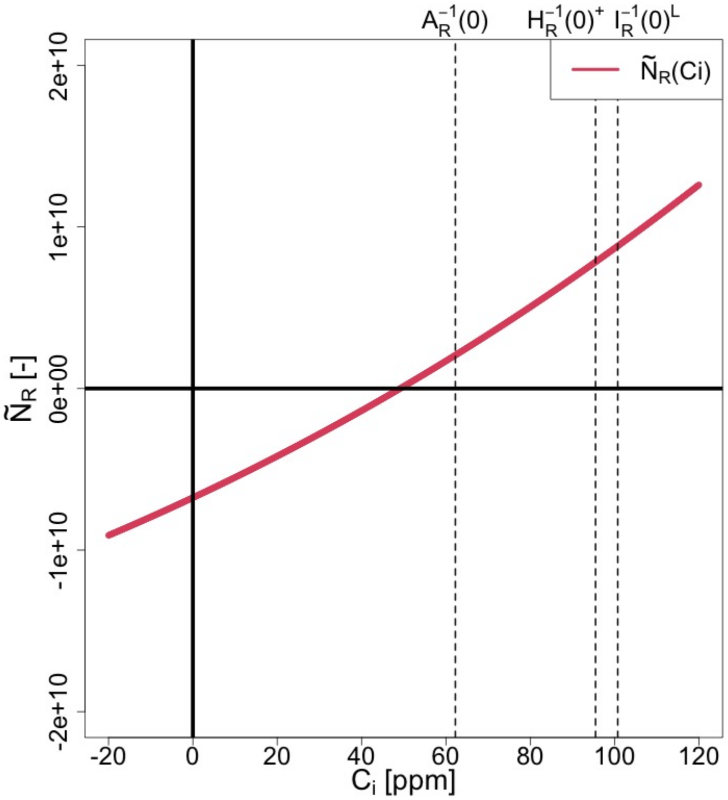

Figure S22: $\tilde{N}_R(C_i)$ for [P2]

(P3) $\tilde{L}_R(A_{nR}^{-1}(0)) < \tilde{M}_R(A_{nR}^{-1}(0))$ and $0 < \tilde{M}'_R(A_{nR}^{-1}(0))$

Eq. (S255) shows that $0 < \tilde{M}'_R(A_{nR}^{-1}(0))$ corresponds to $A_{nR}^{-1}(0) + \frac{\eta}{4} < 0$ under [C1]. Thus, [C1] and $A_{nR}^{-1}(0) + \frac{\eta}{4} < 0$ are assumed in case [P3].

From $\tilde{L}_R(H_R^{-1}(0)^+) > 0$ (Eq. (S202)) and $\tilde{M}_R(H_R^{-1}(0)^+) = 0$ (Eq. (S232)), combined with $\tilde{L}_R(A_{nR}^{-1}(0)) < \tilde{M}_R(A_{nR}^{-1}(0))$, which is the first condition of [P3], we obtain:

$$\tilde{L}_R(A_{nR}^{-1}(0)) < \tilde{M}_R(A_{nR}^{-1}(0)), \quad (S388)$$

$$\tilde{L}_R(H_R^{-1}(0)^+) > \tilde{M}_R(H_R^{-1}(0)^+) = 0. \quad (S389)$$

Therefore, there are one or more intersections between $\tilde{L}_R(C_i)$ and $\tilde{M}_R(C_i)$ within $A_{nR}^{-1}(0) < C_i < H_R^{-1}(0)^+$.

Proposition 4 demonstrates that there is exactly one intersection between $\tilde{L}'_R(C_i)$ and $\tilde{M}'_R(C_i)$ within $0 < C_i \le H_R^{-1}(0)^+$ under [C1] and $A_{nR}^{-1}(0) + \frac{\eta}{4} < 0$. This intersection is denoted as

$\tilde{N}'^{-1}(0)$. From $\tilde{L}'_R(0) < \tilde{M}'_R(0)$ (Eq. (S318)) and $\tilde{L}'_R(H_R^{-1}(0)^+) > \tilde{M}'_R(H_R^{-1}(0)^+)$ (Eq. (S316)) under [C1] and $A_{nR}^{-1}(0) + \frac{\eta}{4} < 0$, we have:

$$
\begin{aligned}
\tilde{L}'_R(0 \leq C_i < \tilde{N}'^{-1}(0)) < \tilde{M}'_R(0 \leq C_i < \tilde{N}'^{-1}(0)), \\
\tilde{L}'_R(\tilde{N}'^{-1}(0)) = \tilde{M}'_R(\tilde{N}'^{-1}(0)), \\
\tilde{L}'_R(\tilde{N}'^{-1}(0) < C_i \leq H_R^{-1}(0)^+) > \tilde{M}'_R(\tilde{N}'^{-1}(0) < C_i \leq H_R^{-1}(0)^+). \\
[\mathrm{C1}, A_{nR}^{-1}(0) + \frac{\eta}{4} < 0]
\end{aligned}
\tag{S390}
$$

We split the analysis into two cases: (A): $\tilde{N}'^{-1}(0) < A_{nR}^{-1}(0)$, and (B): $A_{nR}^{-1}(0) \leq \tilde{N}'^{-1}(0)$.

(A): $\tilde{N}'^{-1}(0) < A_{nR}^{-1}(0)$

In this case, Eq. (S390) implies:

$$
\begin{aligned}
\tilde{L}'_R(A_{nR}^{-1}(0) \leq C_i \leq H_R^{-1}(0)^+) > \tilde{M}'_R(A_{nR}^{-1}(0) \leq C_i \leq H_R^{-1}(0)^+). \\
[\mathrm{C1}, A_{nR}^{-1}(0) + \frac{\eta}{4} < 0, \tilde{N}'^{-1}(0) < A_{nR}^{-1}(0)]
\end{aligned}
\tag{S391}
$$

Combining this with Eqs. (S388) and (S389), we find:

$$\tilde{L}_R(A_{nR}^{-1}(0)) \quad < \quad \tilde{M}_R(A_{nR}^{-1}(0)), \tag{S392}$$

$$\tilde{L}_R(H_R^{-1}(0)^+) \quad > \quad \tilde{M}_R(H_R^{-1}(0)^+) = 0, \tag{S393}$$

$$
\begin{aligned}
\tilde{L}'_R(A_{nR}^{-1}(0) \leq C_i \leq H_R^{-1}(0)^+) > \tilde{M}'_R(A_{nR}^{-1}(0) \leq C_i \leq H_R^{-1}(0)^+). \\
[\mathrm{C1}, A_{nR}^{-1}(0) + \frac{\eta}{4} < 0, \tilde{N}'^{-1}(0) < A_{nR}^{-1}(0)]
\end{aligned}
\tag{S394}
$$

Thus, there is exactly one intersection between $\tilde{L}_R(C_i)$ and $\tilde{M}_R(C_i)$ within $A_{nR}^{-1}(0) < C_i < H_R^{-1}(0)^+$ under [C1], $A_{nR}^{-1}(0) + \frac{\eta}{4} < 0$, $\tilde{L}_R(A_{nR}^{-1}(0)) < \tilde{M}_R(A_{nR}^{-1}(0))$, and $\tilde{N}'^{-1}(0) < A_{nR}^{-1}(0)$.

(B): $A_{nR}^{-1}(0) \leq \tilde{N}'^{-1}(0)$

We divide the range $A_{nR}^{-1}(0) \leq C_i \leq H_R^{-1}(0)^+$ into two sub-ranges: (i) $A_{nR}^{-1}(0) \leq C_i \leq \tilde{N}'^{-1}(0)$, and (ii) $\tilde{N}'^{-1}(0) < C_i \leq H_R^{-1}(0)^+$.

(i): $A_{nR}^{-1}(0) \leq C_i \leq \tilde{N}'^{-1}(0)$

From Eqs. (S388), and (S390), we have:

$$\tilde{L}_R(A_{nR}^{-1}(0)) < \tilde{M}_R(A_{nR}^{-1}(0)), \tag{S395}$$

$$\begin{aligned} \tilde{L}'_R(A_{nR}^{-1}(0) \leq C_i \leq \tilde{N}'^{-1}(0)) \leq \tilde{M}'_R(A_{nR}^{-1}(0) \leq C_i \leq \tilde{N}'^{-1}(0)). \\ [\mathrm{C1}, A_{nR}^{-1}(0) + \frac{\eta}{4} < 0, A_{nR}^{-1}(0) \leq \tilde{N}'^{-1}(0)] \end{aligned} \tag{S396}$$

Therefore, there is no intersection between $\tilde{L}_R(C_i)$ and $\tilde{M}_R(C_i)$ within $A_{nR}^{-1}(0) \leq C_i \leq \tilde{N}'^{-1}(0)$ under [C1], $A_{nR}^{-1}(0) + \frac{\eta}{4} < 0$, $\tilde{L}_R(A_{nR}^{-1}(0)) < \tilde{M}_R(A_{nR}^{-1}(0))$, and $A_{nR}^{-1}(0) \leq \tilde{N}'^{-1}(0)$. Additionally, we find:

$$\begin{aligned} \tilde{L}_R(\tilde{N}'^{-1}(0)) < \tilde{M}_R(\tilde{N}'^{-1}(0)) \\ [\mathrm{C1}, A_{nR}^{-1}(0) + \frac{\eta}{4} < 0, \tilde{L}_R(A_{nR}^{-1}(0)) < \tilde{M}_R(A_{nR}^{-1}(0)), A_{nR}^{-1}(0) \leq \tilde{N}'^{-1}(0)]. \end{aligned} \tag{S397}$$

(ii): $\tilde{N}'^{-1}(0) < C_i \leq H_R^{-1}(0)^+$

From Eqs. (S397), (S389), and (S390), we have:

$$\begin{aligned} \tilde{L}_R(\tilde{N}'^{-1}(0)) < \tilde{M}_R(\tilde{N}'^{-1}(0)), \\ [\mathrm{C1}, A_{nR}^{-1}(0) + \frac{\eta}{4} < 0, \tilde{L}_R(A_{nR}^{-1}(0)) < \tilde{M}_R(A_{nR}^{-1}(0)), A_{nR}^{-1}(0) \leq \tilde{N}'^{-1}(0)] \end{aligned} \tag{S398}$$

$$\tilde{L}_R(H_R^{-1}(0)^+) > \tilde{M}_R(H_R^{-1}(0)^+) = 0, \tag{S399}$$

$$\begin{aligned} \tilde{L}'_R(\tilde{N}'^{-1}(0) \leq C_i \leq H_R^{-1}(0)^+) \geq \tilde{M}'_R(\tilde{N}'^{-1}(0) \leq C_i \leq H_R^{-1}(0)^+). \\ [\mathrm{C1}, A_{nR}^{-1}(0) + \frac{\eta}{4} < 0] \end{aligned} \tag{S400}$$

Therefore, there is one intersection between $\tilde{L}_R(C_i)$ and $\tilde{M}_R(C_i)$ within $\tilde{N}'^{-1}(0) < C_i < H_R^{-1}(0)^+$ under [C1], $A_{nR}^{-1}(0) + \frac{\eta}{4} < 0$, $\tilde{L}_R(A_{nR}^{-1}(0)) < \tilde{M}_R(A_{nR}^{-1}(0))$, and $A_{nR}^{-1}(0) \leq \tilde{N}'^{-1}(0)$.

The results of the two sub-ranges (i) and (ii) show that there is one intersection between $\tilde{L}_R(C_i)$ and $\tilde{M}_R(C_i)$ within $A_{nR}^{-1}(0) < C_i < H_R^{-1}(0)^+$ under [C1], $A_{nR}^{-1}(0) + \frac{\eta}{4} < 0$, $\tilde{L}_R(A_{nR}^{-1}(0)) < \tilde{M}_R(A_{nR}^{-1}(0))$, and $A_{nR}^{-1}(0) < \tilde{N}'^{-1}(0)$.

Consequently, the results of cases (A) and (B) show that there is exactly one intersection between $\tilde{L}_R(C_i)$ and $\tilde{M}_R(C_i)$ within $A_{nR}^{-1}(0) < C_i < H_R^{-1}(0)^+$ under [C1], $A_{nR}^{-1}(0) + \frac{\eta}{4} < 0$,

and $\tilde{L}_R(A_{nR}^{-1}(0)) < \tilde{M}_R(A_{nR}^{-1}(0))$. Thus, we find:

$$\begin{aligned} \tilde{L}_R(A_{nR}^{-1}(0) < C_i < \tilde{N}_R^{-1}(0)) &< \tilde{M}_R(A_{nR}^{-1}(0) < C_i < \tilde{N}_R^{-1}(0)) \\ \tilde{L}_R(\tilde{N}_R^{-1}(0)) &= \tilde{M}_R(\tilde{N}_R^{-1}(0)) \\ \tilde{L}_R(\tilde{N}_R^{-1}(0) < C_i < H_R^{-1}(0)^+) &> \tilde{M}_R(\tilde{N}_R^{-1}(0) < C_i < H_R^{-1}(0)^+) \\ [\text{C1}, A_{nR}^{-1}(0) + \frac{\eta}{4} < 0, \tilde{L}_R(A_{nR}^{-1}(0)) &< \tilde{M}_R(A_{nR}^{-1}(0))] \end{aligned} \tag{S401}$$

Hence,

$$\begin{aligned} \tilde{N}_R(A_{nR}^{-1}(0) < C_i < \tilde{N}_R^{-1}(0)) &< 0 \\ \tilde{N}_R(\tilde{N}_R^{-1}(0)) &= 0 \\ \tilde{N}_R(\tilde{N}_R^{-1}(0) < C_i < H_R^{-1}(0)^+) &> 0 \\ [\text{C1}, A_{nR}^{-1}(0) + \frac{\eta}{4} < 0, \tilde{L}_R(A_{nR}^{-1}(0)) < \tilde{M}_R(A_{nR}^{-1}(0))] \end{aligned} \tag{S402}$$

Figures S23 and S24 show the curves of $\tilde{L}_R(C_i)$ and $\tilde{M}_R(C_i)$, as well as $\tilde{N}_R(C_i)$ for case [P3].

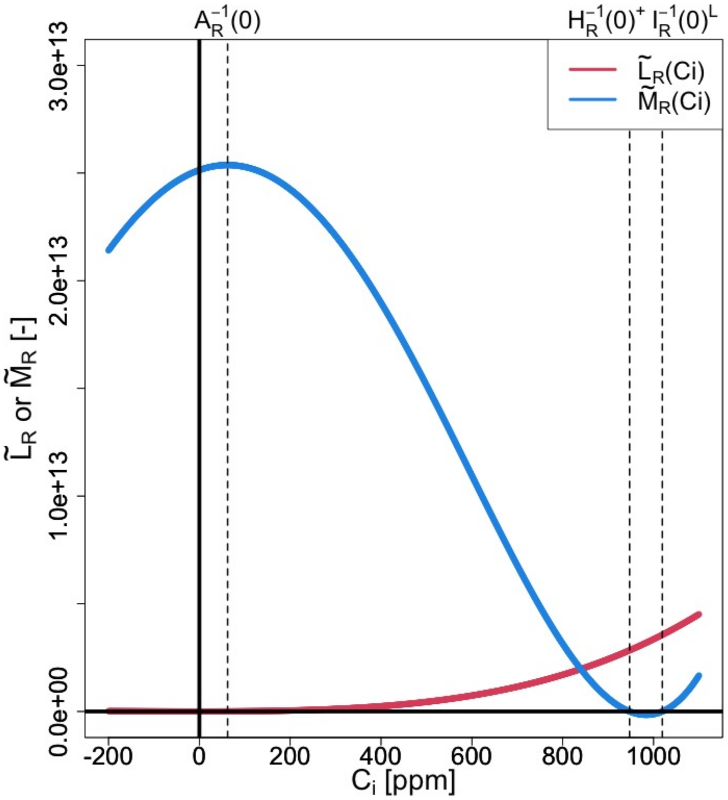

Figure S23: $\tilde{L}_R(C_i)$ and $\tilde{M}_R(C_i)$ for [P3]

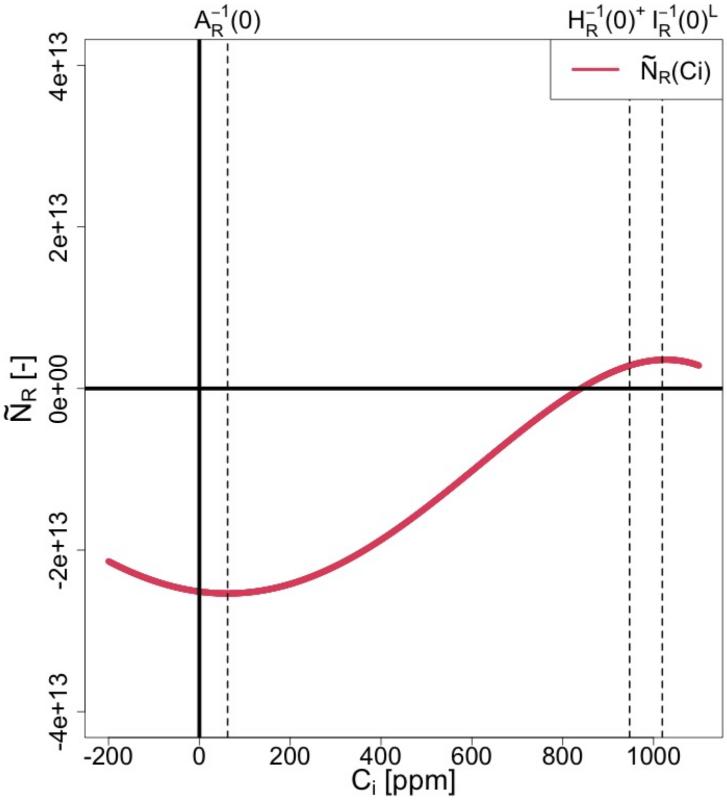

Figure S24: $\tilde{N}_R(C_i)$ for [P3]

(P4) $\tilde{L}_R(A_{nR}^{-1}(0)) \geq \tilde{M}_R(A_{nR}^{-1}(0))$ and $0 < \tilde{M}_R'(A_{nR}^{-1}(0)) < \tilde{L}_R'(A_{nR}^{-1}(0))$

Similar to case [P3], [C1] and $A_{nR}^{-1}(0) + \frac{\eta}{4} < 0$ are assumed since $0 < \tilde{M}_R'(A_{nR}^{-1}(0))$ (Eq. (S255)).

Applying $\tilde{L}_R'(A_{nR}^{-1}(0) \leq C_i \leq H_R^{-1}(0)^+) > \tilde{M}_R'(A_{nR}^{-1}(0) \leq C_i \leq H_R^{-1}(0)^+)$ under [C1], $A_{nR}^{-1}(0)+\frac{\eta}{4} < 0, \tilde{M}_R'(A_{nR}^{-1}(0)) < \tilde{L}_R'(A_{nR}^{-1}(0))$ (Proposition 5), and $\tilde{L}_R(A_{nR}^{-1}(0)) \geq \tilde{M}_R(A_{nR}^{-1}(0))$, along with $\tilde{L}_R(H_R^{-1}(0)^+) > 0$ (Eq. (S202)) and $\tilde{M}_R(H_R^{-1}(0)^+) = 0$ (Eq. (S232)), we have:

$$\tilde{L}_R(A_{nR}^{-1}(0)) \geq \tilde{M}_R(A_{nR}^{-1}(0)) \tag{S403}$$

$$\tilde{L}_R(H_R^{-1}(0)^+) > \tilde{M}_R(H_R^{-1}(0)^+) = 0 \tag{S404}$$

$$\begin{gathered} \tilde{L}_R'(A_{nR}^{-1}(0) \leq C_i \leq H_R^{-1}(0)^+) > \tilde{M}_R'(A_{nR}^{-1}(0) \leq C_i \leq H_R^{-1}(0)^+) \\ [\text{C1}, A_{nR}^{-1}(0) + \frac{\eta}{4} < 0, \tilde{M}_R'(A_{nR}^{-1}(0)) < \tilde{L}_R'(A_{nR}^{-1}(0))] \end{gathered} \tag{S405}$$

Therefore, there is no intersection between $\tilde{L}_R(C_i)$ and $\tilde{M}_R(C_i)$ within the range $A_{nR}^{-1}(0) < C_i < H_R^{-1}(0)^+$ under [C1], $A_{nR}^{-1}(0) + \frac{\eta}{4} < 0$, $\tilde{L}_R(A_{nR}^{-1}(0)) \geq \tilde{M}_R(A_{nR}^{-1}(0))$ and $\tilde{M}_R'(A_{nR}^{-1}(0)) <$

$\tilde{L}'_R(A_{nR}^{-1}(0))$. Thus, we have:

$$\begin{gathered}\tilde{L}_R(A_{nR}^{-1}(0) < C_i < H_R^{-1}(0)^+) > \tilde{M}_R(A_{nR}^{-1}(0) < C_i < H_R^{-1}(0)^+) \\ [\mathrm{C1}, A_{nR}^{-1}(0) + \frac{\eta}{4} < 0, \tilde{L}_R(A_{nR}^{-1}(0)) \geq \tilde{M}_R(A_{nR}^{-1}(0)), \tilde{M}'_R(A_{nR}^{-1}(0)) < \tilde{L}'_R(A_{nR}^{-1}(0))]\end{gathered} \tag{S406}$$

Hence,

$$\begin{gathered}\tilde{N}_R(A_{nR}^{-1}(0) < C_i < H_R^{-1}(0)^+) > 0 \\ [\mathrm{C1}, A_{nR}^{-1}(0) + \frac{\eta}{4} < 0, \tilde{L}_R(A_{nR}^{-1}(0)) \geq \tilde{M}_R(A_{nR}^{-1}(0)), \tilde{M}'_R(A_{nR}^{-1}(0)) < \tilde{L}'_R(A_{nR}^{-1}(0))]\end{gathered} \tag{S407}$$

Figures S25 to S27 show the curves of $\tilde{L}_R(C_i)$, $\tilde{M}_R(C_i)$, and $\tilde{N}_R(C_i)$ for case [P4]

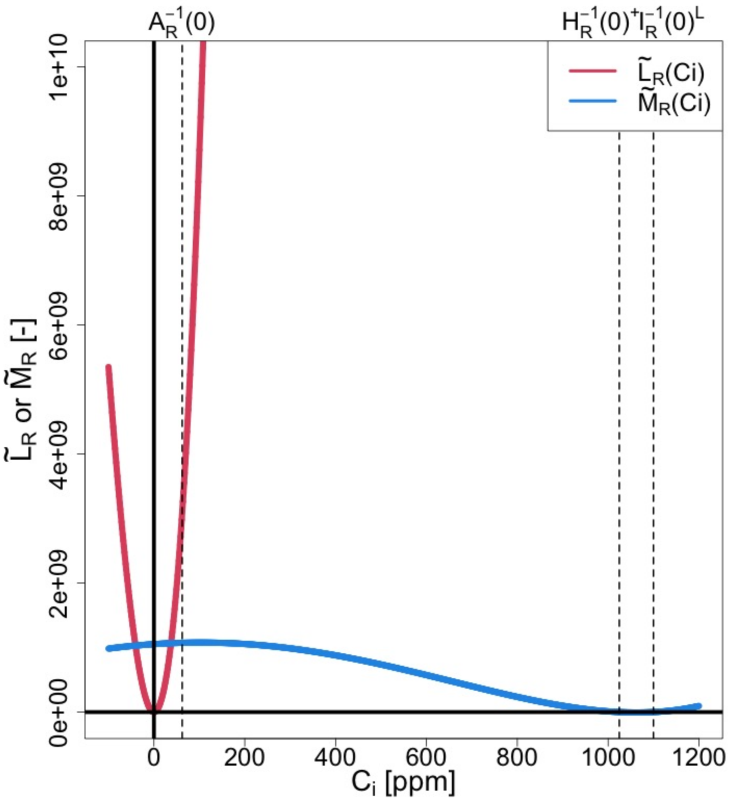

Figure S25: $\tilde{L}_R(C_i)$ and $\tilde{M}_R(C_i)$ for [P4]

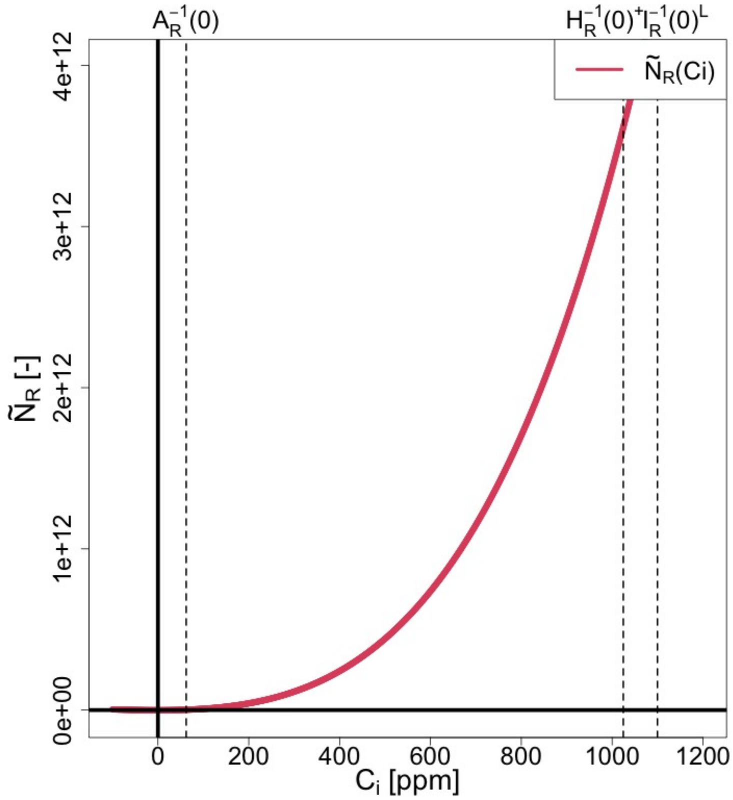

Figure S26: $\tilde{N}_R(C_i)$ for [P4]

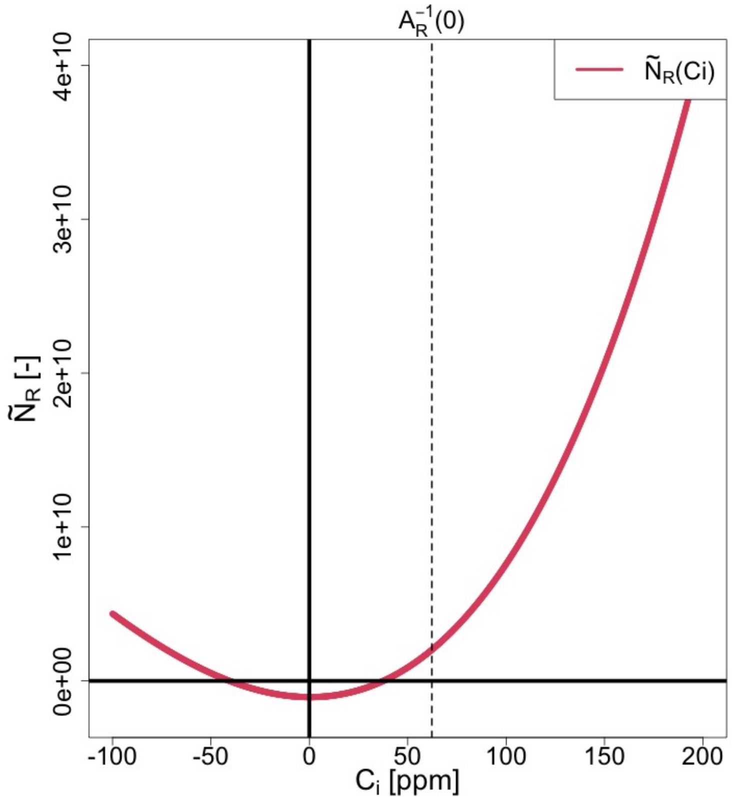

Figure S27: $\tilde{N}_R(C_i)$ at $-100 < C_i < 200$ for [P4]

1853 Table S42 summarizes the number of intersections between $\tilde{L}_R(C_i)$ and $\tilde{M}_R(C_i)$ within the range $A_{nR}^{-1}(0) < C_i < H_R^{-1}(0)^+$ for all four geometric patterns from P1 to P4.

Table S42: The number of intersections between $\tilde{L}_R(C_i)$ and $\tilde{M}_R(C_i)$ at $A_{nR}^{-1}(0) < C_i < H_R^{-1}(0)^+$

Cases	# of Intersections	Type (ZI or ZII)
P1	1	ZI
P2	0	ZII
P3	1	ZI
P4	0	ZII

1855 Table S42 reveals two distinct geometric patterns: one pattern has one intersection, while the other has none within the range $A_{nR}^{-1}(0) < C_i < H_R^{-1}(0)^+$. We denote these patterns as [ZI] for the case with one intersection and [ZII] for the case with none. Table S42 also categorizes each case as

either [ZI] or [ZII]. We can summarize the sign behavior of $\tilde{N}_R(C_i)$ within $A_{nR}^{-1}(0) < C_i < H_R^{-1}(0)^+$ for cases [ZI] and [ZII], as follows:

$$\begin{cases} \tilde{N}_R(A_{nR}^{-1}(0) < C_i < \tilde{N}_R^{-1}(0)) < 0 \\ \tilde{N}_R(\tilde{N}_R^{-1}(0)) = 0 & \text{[ZI]} \\ \tilde{N}_R(\tilde{N}_R^{-1}(0) < C_i < H_R^{-1}(0)^+) > 0 \end{cases} \tag{S408}$$

and,

$$\tilde{N}_R(A_{nR}^{-1}(0) < C_i < H_R^{-1}(0)^+) > 0 \quad \text{[ZII]} \tag{S409}$$

Since $\tilde{L}_R(A_{nR}^{-1}(0)) < \tilde{M}_R(A_{nR}^{-1}(0))$ under [ZI] and $\tilde{L}_R(A_{nR}^{-1}(0)) \geq \tilde{M}_R(A_{nR}^{-1}(0))$ under [ZII] (Table S41), we obtain the following inequalities:

$$\begin{cases} \tilde{N}_R(A_{nR}^{-1}(0)) < 0 \quad \text{[ZI]} \\ \tilde{N}_R(A_{nR}^{-1}(0)) \geq 0 \quad \text{[ZII]} \end{cases} \tag{S410}$$

In case [C1], Eqs. (S358) to (S365) lead to:

$$\text{sgn}(\tilde{N}_R(A_{nR}^{-1}(0))) = -\text{sgn}(C_a - \left\{A_{nR}^{-1}(0) + \frac{A_{nR}^{-1}(0)}{\tilde{g}_{slp}}\right\}) \quad \text{[C1]} \tag{S411}$$

Therefore, Eqs. (S410) and (S411) give conditions for cases [ZI] and [ZII] as:

$$\begin{cases} C_a - \left\{A_{nR}^{-1}(0) + \frac{A_{nR}^{-1}(0)}{\tilde{g}_{slp}}\right\} > 0 \quad \text{[ZI]} \\ C_a - \left\{A_{nR}^{-1}(0) + \frac{A_{nR}^{-1}(0)}{\tilde{g}_{slp}}\right\} \leq 0 \quad \text{[ZII]} \end{cases} \tag{S412}$$

Note that Eq. (S412) was used for the conditions for [Z1] and [Z2] in Table 6.

Finally, given that $\tilde{L}_R(H_R^{-1}(0)^+) > 0$ (Eq. (S202)) and $\tilde{M}_R(H_R^{-1}(0)^+) = 0$ (Eq. (S232)), we obtain:

$$\tilde{N}_R(H_R^{-1}(0)^+) > 0 \tag{S413}$$

SI 5.5 $N_R(C_i)$

$N_R(C_i)$ is given from Eq. (S141) as follows:

$$N_R(C_i) = C_i\{C_i - A_{nR}^{-1}(*)\} + \tilde{g}_{slp}\sqrt{\frac{I_R(C_i)}{H_R(C_i)}}H_R(C_i) \tag{S414}$$

Since the roots and signs of $N_R(C_i)$ are the same as those of $\tilde{N}_R(C_i)$ at $A_{nR}^{-1}(0) \le C_i \le H_R^{-1}(0)^+$ under [C1] (Eqs. (S198) and (S194)), Eqs. (S408) and (S409) lead to:

$$\begin{cases} N_R(A_{nR}^{-1}(0) < C_i < N_R^{-1}(0)) < 0 \\ N_R(N_R^{-1}(0)) = 0 \qquad\qquad\qquad\qquad \text{[ZI]} \\ N_R(N_R^{-1}(0) < C_i < H_R^{-1}(0)^+) > 0 \end{cases} \tag{S415}$$

and,

$$N_R(A_{nR}^{-1}(0) < C_i < H_R^{-1}(0)^+) > 0 \quad \text{[ZII]} \tag{S416}$$

Since $N_R^{-1}(0)$ exist at $A_{nR}^{-1}(0) < C_i < H_R^{-1}(0)^+$ under [ZI], we have

$$A_{nR}^{-1}(0) < N_R^{-1}(0) < H_R^{-1}(0)^+ \quad \text{[ZI]} \tag{S417}$$

In addition, Eqs. (S410) and (S413) lead to:

$$\begin{cases} N_R(A_{nR}^{-1}(0)) < 0 \quad \text{[ZI]} \\ N_R(A_{nR}^{-1}(0)) \ge 0 \quad \text{[ZII]} \end{cases} \tag{S418}$$

$$N_R(H_R^{-1}(0)^+) > 0 \quad \text{[ZI and ZII]} \tag{S419}$$

Figures S28 and S29 show the curves of $N_R(C_i)$ for [ZI] and [ZII]. Tables S43 and S44 show the sign charts of $N_R(C_i)$ for [ZI] and [ZII].

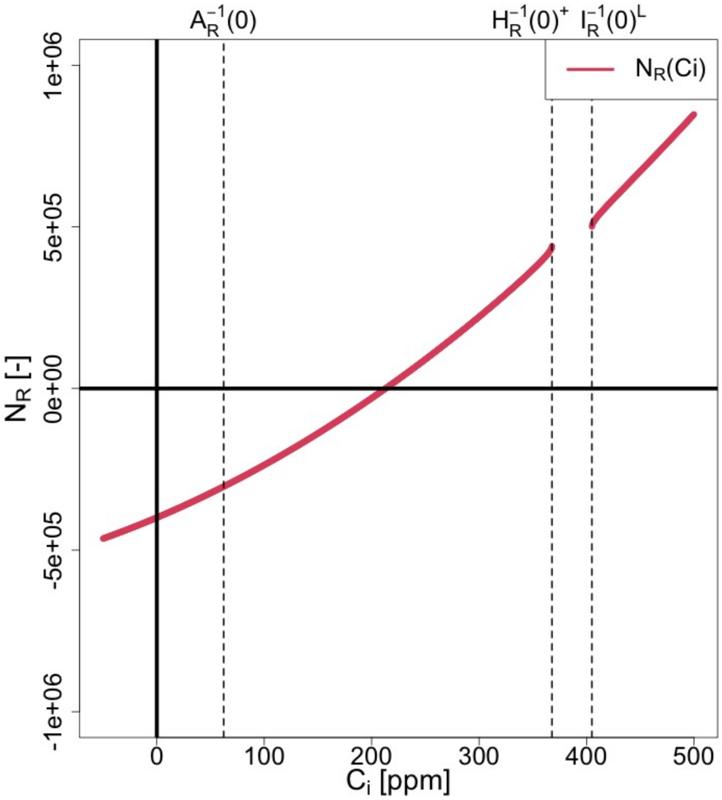

Figure S28: $N_R(C_i)$ for [ZI]

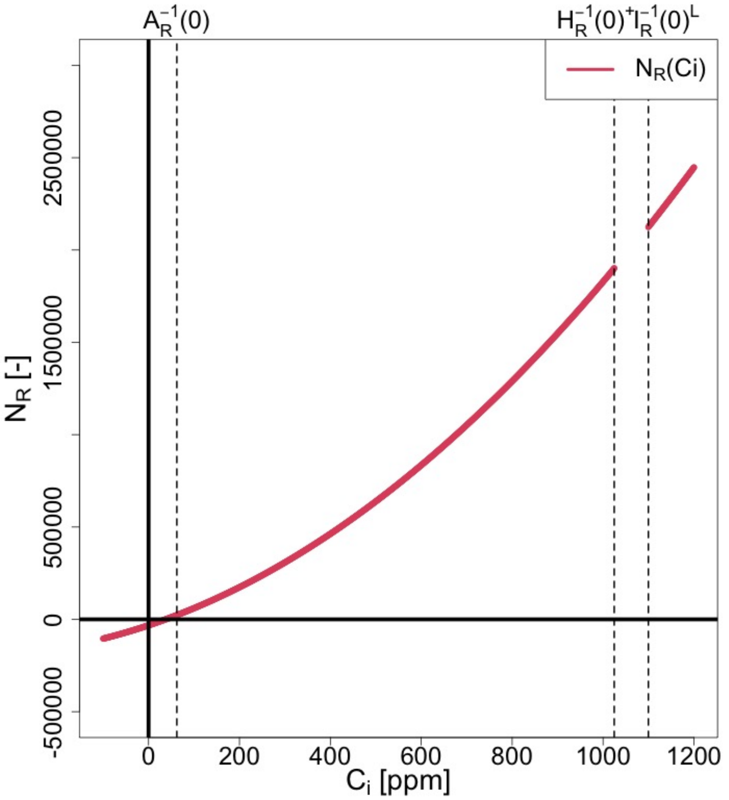

Figure S29: $N_R(C_i)$ for [ZII]

Table S43: Sign chart of $N_R(C_i)$ for ZI

	$A_{nR}^{-1}(0)$		$N_R^{-1}(0)$		$H_R^{-1}(0)^+$
$N_R(C_i)$	-	-	0	+	+

Table S44: Sign chart of $N_R(C_i)$ for ZII

	$A_{nR}^{-1}(0)$		$H_R^{-1}(0)^+$
$N_R(C_i)$	0 or +	+	+

SI 5.6 $N'_R(C_i)$ and $N''_R(C_i)$

In this section, we focus on the region $A_{nR}^{-1}(0) < C_i < H_R^{-1}(0)^+$, which is the possible range of solutions under [C1] (Table 4). Within this range, using $H_R(H_R^{-1}(0)^- < C_i < H_R^{-1}(0)^+) < 0$ (Eq. (S37))

and $H_R^{-1}(0)^- < A_{nR}^{-1}(0) < H_R^{-1}(0)^+$ under [C1] (Eq. (S50)), we derive the following inequality:

$$H_R(A_{nR}^{-1}(0) < C_i < H_R^{-1}(0)^+) < 0 \tag{S420}$$

Additionally, from $I_R(I_R^{-1}(0)^S < C_i < I_R^{-1}(0)^L) < 0$ (Eq. (S159)) and $I_R^{-1}(0)^S < A_{nR}^{-1}(0) < I_R^{-1}(0)^L$ under [C1] (Eqs. (S170) to (S172)), we derive the following inequality:

$$I_R(A_{nR}^{-1}(0) < C_i < H_R^{-1}(0)^+) < 0 \tag{S421}$$

By differentiating Eq. (S141) with respect to C_i, we obtain

$$N_R'(C_i) = 2C_i - A_{nR}^{-1}(*) + \frac{\tilde{g}_{slp}}{2}\left\{I_R'(C_i)\sqrt{\frac{H_R(C_i)}{I_R(C_i)}} + H_R'(C_i)\sqrt{\frac{I_R(C_i)}{H_R(C_i)}}\right\} \tag{S422}$$

In deriving Eq. (S422), Eq. (S144) is used.

Differentiating Eq. (S422) and taking into account Eqs. (S420) and (S421), we obtain:

$$N_R''(C_i) = 2 + \frac{\tilde{g}_{slp}}{2}\left\{\frac{\{H_R(C_i)I_R'(C_i) - H_R'(C_i)I_R(C_i)\}^2}{H_R(C_i)I_R(C_i)\sqrt{H_R(C_i)I_R(C_i)}} + 2\left(\sqrt{\frac{H_R(C_i)}{I_R(C_i)}} + \sqrt{\frac{I_R(C_i)}{H_R(C_i)}}\right)\right\}, \tag{S423}$$

(a) Geometry of $N_R''(C_i)$

From Eq. (S423), we have

$$N_R''(A_{nR}^{-1}(0) < C_i < H_R^{-1}(0)^+) > 0 \tag{S424}$$

Figures S30 and S31 illustrate the curves of $N_R''(C_i)$ for [ZI] and [ZII].

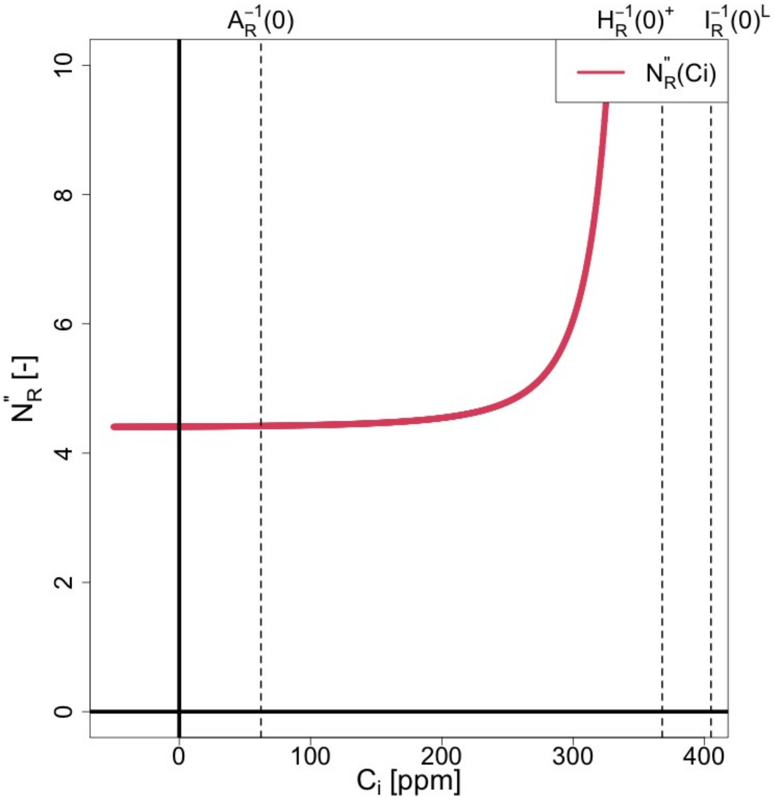

Figure S30: $N_R''(C_i)$ for [ZI]

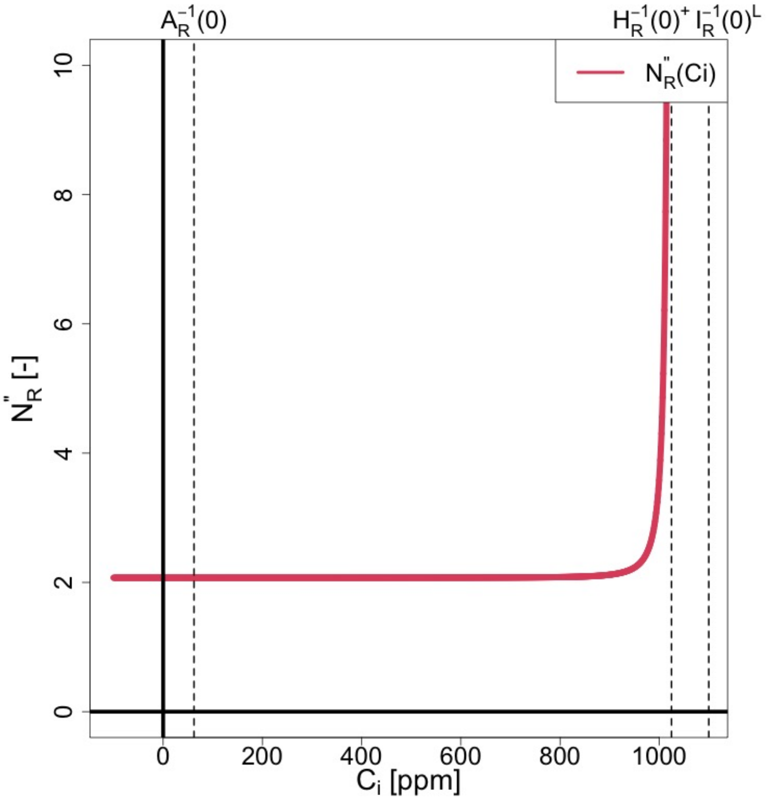

Figure S31: $N_R''(C_i)$ for [ZII]

(b) Singularity: $N_R'^{-1}(*)$

From Eq. (S422), $N_R'^{-1}(*)$ is given by:

$$N_R'^{-1}(*) = H_R^{-1}(0)^+, H_R^{-1}(0)^-, I_R^{-1}(0)^L, \text{ and } I_R^{-1}(0)^S \tag{S425}$$

(c) $N_R'(N_R^{-1}(0))$

Since the sign of $N_R(N_R^{-1}(0))$ changes from negative to positive at $N_R^{-1}(0)$ under [ZI] (Table S43), along with $A_{nR}^{-1}(0) < N_R^{-1}(0) < H_R^{-1}(0)^+$ under [ZI] (Eq. (S417)) and $N_R''(A_{nR}^{-1}(0) < C_i < H_R^{-1}(0)^+) > 0$ (Eq. (S424)), $N_R^{-1}(0)$ must be positive. Thus, we obtain:

$$N_R'(N_R^{-1}(0)) > 0 \quad \text{[ZI]} \tag{S426}$$

(d) $N'_R(A^{-1}_{nR}(0))$

$$N'_R(A^{-1}_{nR}(0)) = 2A^{-1}_{nR}(0) - A^{-1}_{nR}(*) + \tilde{g}_{slp}\frac{H'_R(A^{-1}_{nR}(0)) + I'_R(A^{-1}_{nR}(0))}{2} \tag{S427}$$

$$= 2A^{-1}_{nR}(0) - A^{-1}_{nR}(*) + 2\tilde{g}_{slp}\left\{A^{-1}_{nR}(0) + \frac{\eta}{4}\right\} \tag{S428}$$

Since $A^{-1}_{nR}(0) > 0$ under [C1] (Table 3) and $A^{-1}_{nR}(*) < 0$ (Eq. S7), Eq. (S428) under [C1] and $A^{-1}_{nR}(0) + \frac{\eta}{4} \geq 0$ implies:

$$N'_R(A^{-1}_{nR}(0)) > 0 \quad [\text{C1}, A^{-1}_{nR}(0) + \frac{\eta}{4} \geq 0] \tag{S429}$$

In the case where [C1], $A^{-1}_{nR}(0) + \frac{\eta}{4} < 0$ and $\tilde{L}_R(A^{-1}_{nR}(0)) \geq \tilde{M}_R(A^{-1}_{nR}(0))$, Eq. (S376) is valid. Using Eq. (S376), Eq. (S428) leads to:

$$N'_R(A^{-1}_{nR}(0)) > 0 \quad [\text{C1}, A^{-1}_{nR}(0) + \frac{\eta}{4} < 0 \text{ and } \tilde{L}_R(A^{-1}_{nR}(0)) \geq \tilde{M}_R(A^{-1}_{nR}(0))] \tag{S430}$$

The conditions for Eq. (S429) correspond to [P1] and [P2], while those for Eq. (S430) correspond to [P4] (Table S41 and Eq. (S255)). Thus, we have:

$$N'_R(A^{-1}_{nR}(0)) > 0 \quad [\text{P1, P2, and P4; or P1 and ZII}] \tag{S431}$$

Then, we prove the following proposition:

Proposition 7. *$N'_R(A^{-1}_{nR}(0)) > A^{-1}_{nR}(0)$ under [C1] and $\tilde{L}_R(A^{-1}_{nR}(0)) \geq \tilde{M}_R(A^{-1}_{nR}(0))$.*

Proof. Similar to Eq. (S375), we can derive the following inequality under [C1]:

$$\{1+\tilde{g}_{slp}\}A^{-1}_{nR}(0) < \{1+2\tilde{g}_{slp}\}A^{-1}_{nR}(0) - \{1+\tilde{g}_{slp}\}A^{-1}_{nR}(*) + \tilde{g}_{slp}\{\lambda_b - \frac{\lambda_s}{2}\}\frac{A_{nR}(\pm\infty)}{g_b} \quad [\text{C1}] \tag{S432}$$

In the case where $\tilde{L}_R(A_{nR}^{-1}(0)) \geq \tilde{M}_R(A_{nR}^{-1}(0))$, $\tilde{g}_{slp}C_a \leq \{1+\tilde{g}_{slp}\}A_{nR}^{-1}(0)$ holds under [C1] (Eq. (S364)). Therefore, we obtain:

$$\begin{aligned}
&\tilde{g}_{slp}C_a < \{1+2\tilde{g}_{slp}\}A_{nR}^{-1}(0) - \{1+\tilde{g}_{slp}\}A_{nR}^{-1}(*) + \tilde{g}_{slp}\{\lambda_b - \frac{\lambda_s}{2}\}\frac{A_{nR}(\pm\infty)}{g_b} \\
\Longleftrightarrow\ &A_{nR}^{-1}(0) - A_{nR}^{-1}(*) + 2\tilde{g}_{slp}\left\{A_{nR}^{-1}(0) - \frac{1}{2}\left\{C_a + A_{nR}^{-1}(*) - \left\{\lambda_b - \frac{\lambda_s}{2}\right\}\frac{A_{nR}(\pm\infty)}{g_b}\right\}\right\} > 0 \\
\Longleftrightarrow\ &2A_{nR}^{-1}(0) - A_{nR}^{-1}(*) + 2\tilde{g}_{slp}\left\{A_{nR}^{-1}(0) + \frac{\eta}{4}\right\} - A_{nR}^{-1}(0) > 0 \\
\Longleftrightarrow\ &N_R'(A_{nR}^{-1}(0)) - A_{nR}^{-1}(0) > 0 \\
&[\text{C1}, \tilde{L}_R(A_{nR}^{-1}(0)) \geq \tilde{M}_R(A_{nR}^{-1}(0))]
\end{aligned} \tag{S433}$$

□

(e) Geometry of $N_R'(C_i)$

Figures S32 and S33 illustrate the curves of $N_R'(C_i)$ for [ZI] and [ZII].

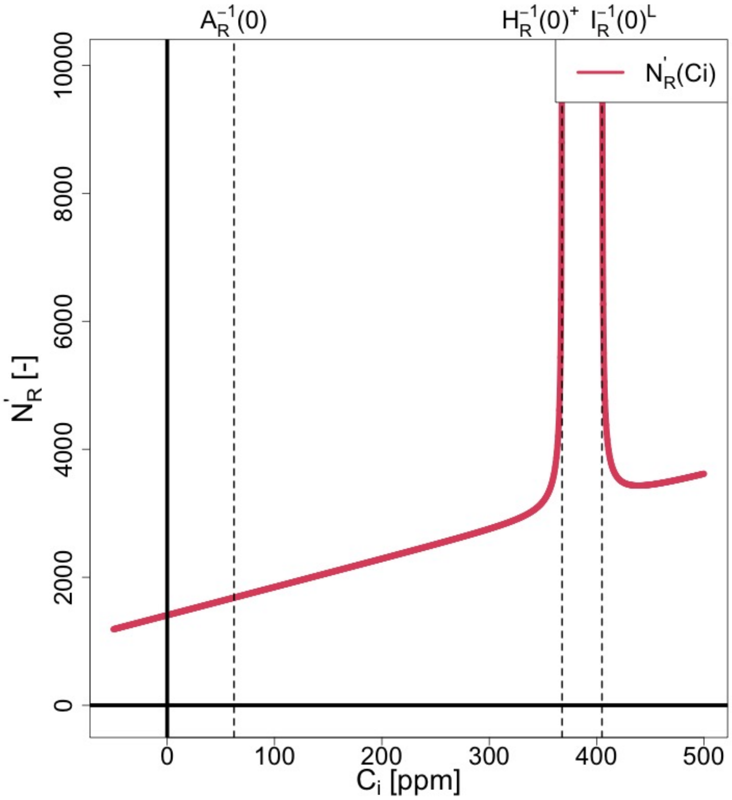

Figure S32: $N'_R(C_i)$ for [ZI]

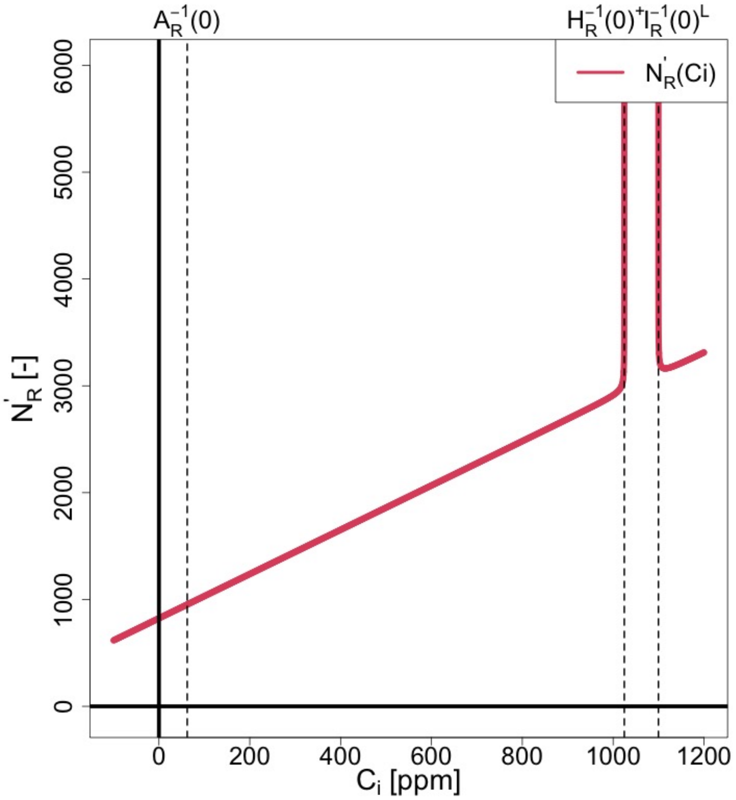

Figure S33: $N'_R(C_i)$ for [ZII]

SI 5.7 $Z_R(C_i)$

$Z_R(C_i)$ is rewritten from Eq. (S140) as follows:

$$Z_R(C_i) = \frac{-\lambda_s A_{nR}(\pm\infty)\{C_i - A_{nR}^{-1}(0)\}N_R(C_i)}{H_R(C_i)K_R(C_i)} \tag{S434}$$

(a) Singularity: $Z_R^{-1}(*)$

From Eq. (S434), we have $Z_R^{-1}(*)$ as follows:

$$Z_R^{-1}(*) = \begin{cases} H_R^{-1}(0)^+, H_R^{-1}(0)^-, \text{ and } K_R^{-1}(0) \\ H_R^{-1}(0)^+ \text{ and } H_R^{-1}(0)^- \end{cases} = \begin{cases} F_R^{-1}(*)^+, F_R^{-1}(*)^-, \text{ and } K_R^{-1}(0) & [\phi \neq 0] \\ F_R^{-1}(0)^+ \text{ and } F_R^{-1}(0)^- & [\phi = 0], \end{cases} \tag{S435}$$

where $F_R^{-1}(*)^{\pm} = H_R^{-1}(0)^{\pm}$ (Eqs. (S87) and (S88)) is used.

To evaluate $Z_R(C_i)$ near $C_i = H_R^{-1}(0)^+$, we note the following: $A_{nR}(\pm\infty) > 0$ under [C1] (Table 3); $H_R^{-1}(0)^+ - \epsilon > A_{nR}^{-1}(0)$ under [C1] (Eq. (S50)); $N_R(H_R^{-1}(0)^+ - \epsilon) > 0$ (Eqs. (S415) and (S416)); $H_R(H_R^{-1}(0)^+ - \epsilon) < 0$ (Eq. (S37)); and $K_R(H_R^{-1}(0)^+ - \epsilon) > 0$ (Eq. (S185)). Since the numerator is positive and the denominator is negative at this point, it follows that

$$Z_R(H_R^{-1}(0)^+ - \epsilon) = +\infty. \tag{S436}$$

(c) Root: $Z_R^{-1}(0)$

From Eq. (S434), we find two roots of $Z_R(C_i)$ under [ZI] and one root under [ZII], as follows:

$$Z_R^{-1}(0) = \begin{cases} A_{nR}^{-1}(0) \text{ and } N_R^{-1}(0) & \text{[ZI]} \\ A_{nR}^{-1}(0) & \text{[ZII]} \end{cases} \tag{S437}$$

To distinguish between the two roots, we denote the roots corresponding to $A_{nR}^{-1}(0)$ and $N_R^{-1}(0)$ as follows:

$$Z_R^{-1}(0)^A = A_{nR}^{-1}(0) = F_R^{-1}(0), \tag{S438}$$

$$Z_R^{-1}(0)^N = N_R^{-1}(0), \tag{S439}$$

where $F_R^{-1}(0) = A_{nR}^{-1}(0)$ (Eq. (S93)) is used.

(d) Geometry: $Z_R(C_i)$

Figure 3 shows the curves of $Z_{RI}(C_i)$ and $Z_{RII}(C_i)$, and Tables S45 and S46 show their sign charts.

Table S45: Sign chart of $Z_R(C_i)$ for [ZI]

	$A_{nR}^{-1}(0)$		$N_R^{-1}(0)$		$H_R^{-1}(0)^+$
$N_R(C_i)$	-	-	0	+	+
$H_R(C_i)$	-	-	-	-	-0
$K_R(C_i)$	+	+	+	+	+
$-\lambda_s A_{nR}(\pm\infty)\{C_i - A_{nR}^{-1}(0)\}$	0	-	-	-	-
$Z_R(C_i)$	0	-	0	+	$+\infty$

Table S46: Sign chart of $Z_R(C_i)$ for [ZII]

	$A_{nR}^{-1}(0)$		$N_R^{-1}(0)$		$H_R^{-1}(0)^+$
$N_R(C_i)$	+	+	+	+	+
$H_R(C_i)$	-	-	-	-	-0
$K_R(C_i)$	+	+	+	+	+
$-\lambda_s A_{nR}(\pm\infty)\{C_i - A_{nR}^{-1}(0)\}$	0	-	-	-	-
$Z_R(C_i)$	0	+	+	+	$+\infty$

SI 5.8 $Z'_R(C_i)$

By differentiating Eq. (S434) with respect to C_i, we have

$$
\begin{aligned}
Z'_R(C_i) =& \frac{-\lambda_s A_{nR}(\pm\infty)}{H_R^2(C_i)K_R^2(C_i)}\Big\{N_R(C_i)H_R(C_i)\big\{K_R(C_i) - \{C_i - A_{nR}^{-1}(0)\}K'_R(C_i)\big\} \\
&+ \{C_i - A_{nR}^{-1}(0)\}K_R(C_i)\big\{N'_R(C_i)H_R(C_i) - N_R(C_i)H'_R(C_i)\big\}\Big\}
\end{aligned}
\quad \text{(S440)}
$$

$$
= \frac{-\lambda_s A_{nR}(\pm\infty)}{H_R^2(C_i)K_R^2(C_i)}\Big\{X_{R1}(C_i) + X_{R2}(C_i)\Big\}, \quad \text{(S441)}
$$

where $X_{R1}(C_i)$ and $X_{R2}(C_i)$ are given by

$$
X_{R1}(C_i) = N_R(C_i)H_R(C_i)\big\{K_R(C_i) - \{C_i - A_{nR}^{-1}(0)\}K'_R(C_i)\big\} \quad \text{(S442)}
$$

$$
X_{R2}(C_i) = \{C_i - A_{nR}^{-1}(0)\}K_R(C_i)\big\{N'_R(C_i)H_R(C_i) - N_R(C_i)H'_R(C_i)\big\} \quad \text{(S443)}
$$

SI 5.8.1 $X_{R1}(C_i)$

$$
\begin{aligned}
X_{R1}(C_i) &= N_R(C_i)H_R(C_i)\big\{K_R(C_i) - \{C_i - A_{nR}^{-1}(0)\}K'_R(C_i)\big\} && \text{(S444)} \\
&= N_R(C_i)H_R(C_i)K_R(A_{nR}^{-1}(0)) && \text{(S445)}
\end{aligned}
$$

Given that $A_{nR}^{-1}(0) < H_R^{-1}(0)^+$ under [C1] (Eq. S50); $H_R(A_{nR}^{-1}(0) < C_i < H_R^{-1}(0)^+) < 0$ (Eq. (S37)); $K_R(A_{nR}^{-1}(0)) > 0$ under [C1] (Eq. (S188)); $N_R(N_R^{-1}(0) < C_i < H_R^{-1}(0)^+) > 0$ under [ZI] (Eq. (S415)); and $N_R(A_{nR}^{-1}(0) < C_i < H_R^{-1}(0)^+) > 0$ under [ZII] (Eq. (S416)), we find:

$$X_{R1}(N_R^{-1}(0) < C_i < H_R^{-1}(0)^+) \quad < \quad 0 \quad [\text{ZI}] \tag{S446}$$

$$X_{R1}(A_{nR}^{-1}(0) < C_i < H_R^{-1}(0)^+) \quad < \quad 0 \quad [\text{ZII}] \tag{S447}$$

We note that the conditions for [C1] are the subset of those for [Z1] and [Z2] (Table 6).

SI 5.8.2 $X_{R2}(C_i)$

$$\begin{aligned} X_{R2}(C_i) &= \{C_i - A_{nR}^{-1}(0)\}K_R(C_i)\left\{N_R'(C_i)H_R(C_i) - N_R(C_i)H_R'(C_i)\right\} && \text{(S448)} \\ &= \tilde{X}_{R1}(C_i)\tilde{X}_{R2}(C_i) && \text{(S449)} \end{aligned}$$

where $\tilde{X}_{R1}(C_i)$ and $\tilde{X}_{R2}(C_i)$ are given by

$$\begin{aligned} \tilde{X}_{R1}(C_i) &= \{C_i - A_{nR}^{-1}(0)\}K_R(C_i) && \text{(S450)} \\ \tilde{X}_{R2}(C_i) &= N_R'(C_i)H_R(C_i) - N_R(C_i)H_R'(C_i) && \text{(S451)} \end{aligned}$$

SI 5.8.3 $\tilde{X}_{R1}(C_i)$

Since $K_R(A_{nR}^{-1}(0) < C_i < H_R^{-1}(0)^+) > 0$ under [C1] (Eq. (S185)), Eq. (S450) leads to:

$$\tilde{X}_{R1}(A_{nR}^{-1}(0) < C_i < H_R^{-1}(0)^+) > 0 \quad [\text{C1}] \tag{S452}$$

SI 5.8.4 $\tilde{X}_{R2}'(C_i)$

By differentiating $\tilde{X}_{R2}(C_i)$ (Eq. (S451)) with respect to C_i, we obtain:

$$\tilde{X}_{R2}'(C_i) = N_R''(C_i)H_R(C_i) - 2N_R(C_i) \tag{S453}$$

In case of [ZI], since $H_R^{-1}(0)^- < A_{nR}^{-1}(0) < N_R^{-1}(0) < H_R^{-1}(0)^+$ (Eqs. (S50) and (S417)), $N_R''(N_R^{-1}(0) < C_i < H_R^{-1}(0)^+) > 0$ (Eq. (S424)), $H_R(N_R^{-1}(0) < C_i < H_R^{-1}(0)^+) < 0$ (Eq. (S37)), and $N_R(N_R^{-1}(0) < C_i < H_R^{-1}(0)^+) > 0$ (Eq. (S415)), Eq. (S453) leads to:

$$\tilde{X}_{R2}'(N_R^{-1}(0) < C_i < H_R^{-1}(0)^+) < 0 \quad \text{[ZI]} \tag{S454}$$

In case of [ZII], since $H_R^{-1}(0)^- < A_{nR}^{-1}(0) < H_R^{-1}(0)^+$ (Eq. (S50)), $N_R''(A_{nR}^{-1}(0) < C_i < H_R^{-1}(0)^+) > 0$ (Eq. (S424)), $H_R(A_{nR}^{-1}(0) < C_i < H_R^{-1}(0)^+) < 0$ (Eq. (S37)), and $N_R(A_{nR}^{-1}(0) < C_i < H_R^{-1}(0)^+) > 0$ (Eq. (S416)), Eq. (S453) leads to:

$$\tilde{X}_{R2}'(A_{nR}^{-1}(0) < C_i < H_R^{-1}(0)^+) < 0 \quad \text{[ZII]} \tag{S455}$$

SI 5.8.5 $\tilde{X}_{R2}(C_i)$

$\tilde{X}_{R2}(C_i)$ is given from Eq. (S451) as:

$$\tilde{X}_{R2}(C_i) = N_R'(C_i)H_R(C_i) - N_R(C_i)H_R'(C_i) \tag{S456}$$

(a) $\tilde{X}_{R2}(H_R^{-1}(0)^+)$

$$\tilde{X}_{R2}(H_R^{-1}(0)^+) = -N_R(H_R^{-1}(0)^+)H_R'(H_R^{-1}(0)^+) \tag{S457}$$

Given that $N_R(H_R^{-1}(0)^+) > 0$ (Eq. (S419)) and $H_R'(H_R^{-1}(0)^+) > 0$ (Eq. (S83)), we obtain:

$$\tilde{X}_{R2}(H_R^{-1}(0)^+) < 0 \tag{S458}$$

(b) $\tilde{X}_{R2}(N_R^{-1}(0))$

[ZI] is assumed to calculate $\tilde{X}_{R2}(N_R^{-1}(0))$, since $N_R^{-1}(0)$ exists under [ZI]. From Eq. (S456), $\tilde{X}_{R2}(N_R^{-1}(0))$ is given by:

$$\tilde{X}_{R2}(N_R^{-1}(0)) = N_R'(N_R^{-1}(0))H_R(N_R^{-1}(0)) \quad \text{[ZI]} \tag{S459}$$

Since $A_{nR}^{-1}(0) < N_R^{-1}(0) < H_R^{-1}(0)^+$ under [ZI] (Eq. (S417)), $N_R'(N_R^{-1}(0)) > 0$ (Eq. (S426)) and $H_R(A_{nR}^{-1}(0) < C_i < H_R^{-1}(0)^+) < 0$ (Eq. (S37)), we obtain:

$$\tilde{X}_{R2}(N_R^{-1}(0)) < 0 \quad \text{[ZI]} \tag{S460}$$

(c) $\tilde{X}_{R2}(A_{nR}^{-1}(0))$ under [ZII]

In this section, [ZII] is assumed to calculate $\tilde{X}_{R2}(A_{nR}^{-1}(0))$. From Eq. (S456), $\tilde{X}_{R2}(A_{nR}^{-1}(0))$ is given by

$$\tilde{X}_{R2}(A_{nR}^{-1}(0)) = N_R'(A_{nR}^{-1}(0))H_R(A_{nR}^{-1}(0)) - N_R(A_{nR}^{-1}(0))H_R'(A_{nR}^{-1}(0)) \tag{S461}$$

$$= \{A_{nR}^{-1}(0) - A_{nR}^{-1}(*)\}\psi(C_a) \tag{S462}$$

In Eq. (S462), we introduce $\psi(C_a)$, a linear function of C_a given by

$$\psi(C_a) = \psi_{C_a}C_a + \psi_{A_{nR}^{-1}(0)}A_{nR}^{-1}(0). \tag{S463}$$

where

$$\psi_{C_a} = -\{\tilde{\psi} - A_{nR}^{-1}(0)\}, \tag{S464}$$

$$\psi_{A_{nR}^{-1}(0)} = \tilde{\psi} - \tilde{C}_a, \tag{S465}$$

$$\tilde{\psi} = N_R'(A_{nR}^{-1}(0)) - \tilde{g}_{slp}H_R'(A_{nR}^{-1}(0)) \tag{S466}$$

$$\tilde{C}_a = 2A_{nR}^{-1}(0) - A_{nR}^{-1}(*) + \lambda_b\frac{A_{nR}(\pm\infty)}{g_b}. \tag{S467}$$

Since $A_{nR}^{-1}(*) < 0$ (Eq. (S7)) and $A_{nR}(\pm\infty) > 0$ under [C1] (Table 3), Eq. (S467) leads to:

$$A_{nR}^{-1}(0) < \tilde{C}_a \quad \text{[C1]} \tag{S468}$$

We consider two cases for $H_R'(A_{nR}^{-1}(0))$: $H_R'(A_{nR}^{-1}(0)) \geq 0$ and $H_R'(A_{nR}^{-1}(0)) < 0$.

(i) $H_R'(A_{nR}^{-1}(0)) \geq 0$

Since $N_R'(A_{nR}^{-1}(0)) > 0$ under [ZII] (Eq. (S431)), $H_R(A_{nR}^{-1}(0)) < 0$ (Eq. (S37)), and $N_R(A_{nR}^{-1}(0)) \geq 0$ under [ZII] (Eq. (S418)), Eq. (S461) leads to:

$$\tilde{X}_{R2}(A_{nR}^{-1}(0)) < 0 \quad [\text{ZII, and } H_R'(A_{nR}^{-1}(0)) \geq 0] \tag{S469}$$

(ii) $H_R'(A_{nR}^{-1}(0)) < 0$

$$H_R'(A_{nR}^{-1}(0)) < 0 \tag{S470}$$

$$\Longleftrightarrow \quad 2A_{nR}^{-1}(0) - \{C_a + A_{nR}^{-1}(*) - \lambda_b \tfrac{A_{nR}(\pm\infty)}{g_b}\} < 0 \tag{S471}$$

$$\Longleftrightarrow \quad 2A_{nR}^{-1}(0) - A_{nR}^{-1}(*) + \lambda_b \tfrac{A_{nR}(\pm\infty)}{g_b} < C_a \tag{S472}$$

$$\Longleftrightarrow \quad \tilde{C}_a < C_a, \tag{S473}$$

Eq. (S473) shows that $\tilde{C}_a$ represents the lower limit of C_a for the case $H_R'(A_{nR}^{-1}(0)) < 0$.

$A_{nR}^{-1}(0) + \frac{\eta}{4}$ can be represented as a linear function of C_a with a negative slope, as follows:

$$A_{nR}^{-1}(0) + \frac{\eta}{4} = \Phi(C_a) = -\frac{1}{2}C_a + A_{nR}^{-1}(0) - \frac{1}{2}\left\{A_{nR}^{-1}(*) - \left(\lambda_b - \frac{\lambda_s}{2}\right)\frac{A_{nR}(\pm\infty)}{g_b}\right\}. \tag{S474}$$

Here $\Phi(C_a)$ was introduced. $\Phi(\tilde{C}_a)$ is the maximum under $H_R'(A_{nR}^{-1}(0)) < 0$, given by:

$$\Phi(\tilde{C}_a) = -\frac{\lambda_s A_{nR}(\pm\infty)}{4g_b}. \tag{S475}$$

Since $A_{nR}(\pm\infty) > 0$ under [C1] (Table 3), we have

$$\Phi(\tilde{C}_a) < 0 \quad [\text{C1}, H'_R(A_{nR}^{-1}(0)) < 0] \tag{S476}$$

Thus we have:

$$\Phi(\tilde{C}_a < C_a) < 0 \quad [\text{C1}, H'_R(A_{nR}^{-1}(0)) < 0] \tag{S477}$$

Combining Eqs. (S473), (S474), and (S477), we obtain:

$$A_{nR}^{-1}(0) + \frac{\eta}{4} < 0 \quad [\text{C1}, H'_R(A_{nR}^{-1}(0)) < 0] \tag{S478}$$

Since $\text{sgn}(A_{nR}^{-1}(0) + \frac{\eta}{4}) = -\text{sgn}(\tilde{M}'_R(A_{nR}^{-1}(0)))$ under [C1] (Eq. (S255)), Eq. (S478) leads to

$$\tilde{M}'_R(A_{nR}^{-1}(0)) > 0 \quad [\text{C1}, H'_R(A_{nR}^{-1}(0)) < 0] \tag{S479}$$

$\tilde{M}'_R(A_{nR}^{-1}(0)) > 0$ represents the case [P4] under [ZII] (Table S41). Thus only [P4] is considered in the case $H'_R(A_{nR}^{-1}(0)) < 0$. Since $N'_R(A_{nR}^{-1}(0)) > 0$ under [P4] (Eq. (S431)), Eq. (S466) shows:

$$\tilde{\psi} > 0 \quad [\text{ZII and } H'_R(A_{nR}^{-1}(0)) < 0] \tag{S480}$$

Using Eqs. (S464) and (S466), we have:

$$\psi_{C_a} = -\left\{N'_R(A_{nR}^{-1}(0)) - A_{nR}^{-1}(0) - \tilde{g}_{slp}H'_R(A_{nR}^{-1}(0))\right\} \tag{S481}$$

From $N'_R(A_{nR}^{-1}(0)) > A_{nR}^{-1}(0)$ under [ZII] (Proposition 7) and $H'_R(A_{nR}^{-1}(0)) < 0$, we obtain:

$$\psi_{C_a} < 0 \quad [\text{ZII and } H'_R(A_{nR}^{-1}(0)) < 0] \tag{S482}$$

Therefore, the slope of $\psi(C_a)$ is negative. Hence, $\psi(\tilde{C}_a)$ is maximized under [ZII] and $H'_R(A_{nR}^{-1}(0)) < 0$ as follows:

$$\psi(\tilde{C}_a) = -\tilde{\psi}\{\tilde{C}_a - A_{nR}^{-1}(0)\} \quad \text{(S483)}$$

Since $A_{nR}^{-1}(0) < \tilde{C}_a$ under [C1] (Eq. (S468)) and $\tilde{\psi} > 0$ under [ZII] and $H'_R(A_{nR}^{-1}(0)) < 0$ (Eq. (S480)), we have:

$$\psi(\tilde{C}_a) < 0 \quad [\text{ZII and } H'_R(A_{nR}^{-1}(0)) < 0] \quad \text{(S484)}$$

Thus, we obtain:

$$\psi(\tilde{C}_a < C_a) < 0 \quad [\text{ZII and } H'_R(A_{nR}^{-1}(0)) < 0] \quad \text{(S485)}$$

Since $A_{nR}^{-1}(0) > A_{nR}^{-1}(*)$ under [C1] (Table 3) and $\psi(\tilde{C}_a < C_a) < 0$ (Eq. (S485)), Eq. (S462) yields:

$$\tilde{X}_{R2}(A_{nR}^{-1}(0)) < 0 \quad [\text{ZII and } H'_R(A_{nR}^{-1}(0)) < 0] \quad \text{(S486)}$$

(d) $\tilde{X}_{R2}(N_R^{-1}(0) < C_i < H_R^{-1}(0)^+)$ and $\tilde{X}_{R2}(A_{nR}^{-1}(0) < C_i < H_R^{-1}(0)^+)$

From $\tilde{X}'_{R2}(N_R^{-1}(0) < C_i < H_R^{-1}(0)^+) < 0$ under [ZI] (Eq. (S454)) and $\tilde{X}_{R2}(N_R^{-1}(0)) < 0$ under [ZI] (Eq. (S460)), we obtain:

$$\tilde{X}_{R2}(N_R^{-1}(0) < C_i < H_R^{-1}(0)^+) < 0 \quad [\text{ZI}] \quad \text{(S487)}$$

From $\tilde{X}'_{R2}(A_{nR}^{-1}(0) < C_i < H_R^{-1}(0)^+) < 0$ under [ZII] (Eq. (S455)) and $\tilde{X}_{R2}(A_{nR}^{-1}(0)) < 0$

under [ZII] (Eqs. (S469) and (S486)), we obtain:

$$\tilde{X}_{R2}(A_{nR}^{-1}(0) < C_i < H_R^{-1}(0)^+) < 0 \quad \text{[ZII]} \tag{S488}$$

SI 5.8.6 Sign of $Z'_R(C_i)$

From $\tilde{X}_{R1}(A_{nR}^{-1}(0) < C_i < H_R^{-1}(0)^+) > 0$ (Eq. (S452)), $A_{nR}^{-1}(0) < N_R^{-1}(0) < H_R^{-1}(0)^+$ under [Z1] (Eq. (S417)), $\tilde{X}_{R2}(N_R^{-1}(0) < C_i < H_R^{-1}(0)^+) < 0$ under [ZI] (Eq. (S487)), and $\tilde{X}_{R2}(A_{nR}^{-1}(0) < C_i < H_R^{-1}(0)^+) < 0$ under [ZII] (Eq. (S488)), Eq. (S449) gives:

$$\begin{cases} X_{R2}(N_R^{-1}(0) < C_i < H_R^{-1}(0)^+) < 0 \quad \text{[ZI]} \\ X_{R2}(A_{nR}^{-1}(0) < C_i < H_R^{-1}(0)^+) < 0 \quad \text{[ZII]} \end{cases} \tag{S489}$$

From $A_{nR}(\pm\infty) > 0$ (Table 3); $X_{R1}(N_R^{-1}(0) < C_i < H_R^{-1}(0)^+) < 0$ and $X_{R2}(N_R^{-1}(0) < C_i < H_R^{-1}(0)^+) < 0$ under [ZI] (Eqs. (S446) and (S489)); $X_{R1}(A_{nR}^{-1}(0) < C_i < H_R^{-1}(0)^+) < 0$ and $X_{R2}(A_{nR}^{-1}(0) < C_i < H_R^{-1}(0)^+) < 0$ under [ZII] (Eqs. (S447) and (S489)), Eq. (S441) gives:

$$\begin{cases} Z'_R(N_R^{-1}(0) < C_i < H_R^{-1}(0)^+) > 0 \quad \text{[ZI]} \\ Z'_R(A_{nR}^{-1}(0) < C_i < H_R^{-1}(0)^+) > 0 \quad \text{[ZII]} \end{cases} \tag{S490}$$

Therefore, $Z_R(C_i)$ increases monotonically for $N_R^{-1}(0) < C_i < H_R^{-1}(0)^+$ under [ZI], and for $A_{nR}^{-1}(0) < C_i < H_R^{-1}(0)^+$ under [ZII]. By using $N_R^{-1}(0) = Z_R^{-1}(0)^N$ (Eq. (S439)), $A_{nR}^{-1}(0) = F_R^{-1}(0)$ (Eq. (S93)), and $H_R^{-1}(0)^+ = F_R^{-1}(*)^+$ (Eq. (S87)), we have:

$$\begin{cases} Z'_R(Z_R^{-1}(0)^N < C_i < F_R^{-1}(*)^+) > 0 \quad \text{[ZI]} \\ Z'_R(F_R^{-1}(0) < C_i < F_R^{-1}(*)^+) > 0 \quad \text{[ZII]} \end{cases} \tag{S491}$$

SI 6 $Z_P(C_i)$ [TPU-limited]

The possible range of appropriate solutions for $F_P(C_i)$ is $0 < C_i < H_P^{-1}(0)$ (Table 4). Therefore, in this chapter, we focus on the range $0 < C_i < H_P^{-1}(0)$.

Using Eq. (22) and Eq. (S124), $Z_P(C_i)$, defined by $F_P(C_i) - G_P(C_i)$ (Table 5), can be expressed as:

$$Z_P(C_i) = F_P(C_i) - G_P(C_i) \tag{S492}$$

$$= \frac{\lambda_s g_b A_{nP}}{H_P(0) H_P(C_i)} \left\{ g_b C_i + \tilde{g}_{slp} \sqrt{\frac{I_P(C_i)}{H_P(C_i)}} H_P(C_i) \right\} \tag{S493}$$

$$= \frac{\lambda_s g_b A_{nP}}{H_P(0) H_P(C_i)} \left\{ g_b C_i - \tilde{g}_{slp} \sqrt{H_P(C_i) I_P(C_i)} \right\} \tag{S494}$$

$$= \frac{\lambda_s g_b A_{nP} N_P(C_i)}{H_P(0) H_P(C_i)} \tag{S495}$$

where $I_P(C_i)$ and $N_P(C_i)$ are given by

$$I_P(C_i) = g_b \{C_i - C_a\} + \{\lambda_b - \lambda_s\} A_{nP} \tag{S496}$$

$$N_P(C_i) = \left\{ g_b C_i - \tilde{g}_{slp} \sqrt{H_P(C_i) I_P(C_i)} \right\} \tag{S497}$$

Note that $H_P(C_i < H_P^{-1}(0)) < 0$ (Eq. (S127)) is used to obtain Eq. (S494).

SI 6.1 $I_P(C_i)$

(a) $I_P(H_P^{-1}(0))$

$$I_P(H_P^{-1}(0)) = -\lambda_s A_{nP} \quad (< 0) \tag{S498}$$

Since $I_P(C_i)$ is a linear function of C_i with a positive slope, Eq. (S498) leads to:

$$I_P(0 < C_i < H_P^{-1}(0)) < 0 \tag{S499}$$

SI 6.2 $N_P(C_i)$

(a) $N_P(0)$ and $N_P(H_P^{-1}(0))$

$$N_P(0) = -\tilde{g}_{slp}\sqrt{H_P(0)I_P(0)} \quad (< 0) \tag{S500}$$

$$N_P(H_P^{-1}(0)) = g_b H_P^{-1}(0) \quad (> 0) \tag{S501}$$

We used $0 < H_P^{-1}(0)$ (Eq. (S131)) for the inequality in Eq. (S501).

(b) $N'_P(C_i)$

The derivative of $N_P(C_i)$ is given by

$$N'_P(C_i) = g_b - \frac{g_b\tilde{g}_{slp}\{H_P(C_i) + I_P(C_i)\}}{2\sqrt{H_P(C_i)I_P(C_i)}} \tag{S502}$$

Since $H_P(0 < C_i < H_P^{-1}(0)) < 0$ (Eq. (S127)) and $I_P(0 < C_i < H_P^{-1}(0)) < 0$ (Eq. (S499)), Eq. (S502) leads to:

$$N'_P(0 < C_i < H_P^{-1}(0)) > 0 \tag{S503}$$

(c) Root: $N_P^{-1}(0)$

Proposition 8. *Only one root for $N_P(C_i) = 0$ exists at $0 < C_i < H_P^{-1}(0)$.*

Proof. From $N_P(0) < 0$ (Eq. (S500)), $N_P(H_P^{-1}(0)) > 0$ (Eq. (S501)), and $N'_P(0 < C_i < H_P^{-1}(0)) > 0$ (Eq. (S503)), there is only one root for $N_P(C_i) = 0$ at $0 < C_i < H_P^{-1}(0)$. This proves the proposition. □

Proposition 8 shows that the root, $N_P^{-1}(0)$, satisfies the following inequality:

$$0 < N_P^{-1}(0) < H_P^{-1}(0) \tag{S504}$$

In addition, from $N_P(0) < 0$ (Eq. (S500)) and $N_P(H_P^{-1}(0)) > 0$ (Eq. (S501)), we have

$$N_P(N_P^{-1}(0) < C_i < H_P^{-1}(0)) > 0 \tag{S505}$$

$$N_P(0 < C_i < N_P^{-1}(0)) < 0 \tag{S506}$$

SI 6.3 $Z_P(C_i)$

$Z_P(C_i)$ can be rewritten from Eq. (S495) as follows:

$$Z_P(C_i) = \frac{\lambda_s g_b A_{nP} N_P(C_i)}{H_P(0) H_P(C_i)} \tag{S507}$$

(a) Singularity: $Z_P^{-1}(*)$

Eq. (S507) shows that the singularity of $Z_P(C_i)$ is given by

$$Z_P^{-1}(*) = H_P^{-1}(0) = F_P^{-1}(*), \tag{S508}$$

where $F_P^{-1}(*) = H_P^{-1}(0)$ (Eq. (S136)) is used.

(b) Root: $Z_P^{-1}(0)$

Eq. (S507) shows that the roots of $Z_P(C_i)$ are the same as those of $N_P(C_i)$, as follows:

$$Z_P^{-1}(0) = N_P^{-1}(0) \tag{S509}$$

Proposition 8 shows that only one $N_P^{-1}(0)$ exists at $0 < C_i < H_P^{-1}(0)$. Since $Z_P^{-1}(0) = N_P^{-1}(0)$ (Eq. (S509)), there is only one $Z_P^{-1}(0)$ in the range $0 < C_i < H_P^{-1}(0)$.

Given that $A_{nP} > 0$ (Eq. (16)), $H_P(0) < 0$ (Eq. (S133)), $H_P(C_i < H_P^{-1}(0)) < 0$ (Eq. (S127)), and $N_P(C_i < N_P^{-1}(0)) < 0$ (Eq. (S506)), Eq. (S507) implies:

$$Z_P(0 < C_i < N_P^{-1}(0)) < 0 \tag{S510}$$

SI 6.4 $Z'_P(C_i)$

$$Z'_P(C_i) = \frac{\lambda_s g_b A_{nP}}{H_P(0)} \left\{ \frac{N'_P(C_i) H_P(C_i) - g_b N_P(C_i)}{H_P^2(C_i)} \right\} \quad \text{(S511)}$$

From $A_{nP} > 0$ (Eq. (16)), $H_P(0) < 0$ (Eq. (S133)), $N'_P(N_P^{-1}(0) < C_i < H_P^{-1}(0)) > 0$ (Eq. (S503)), $H_P(N_P^{-1}(0) < C_i < H_P^{-1}(0)) < 0$ (Eq. (S127)), and $N_P(N_P^{-1}(0) < C_i < H_P^{-1}(0)) > 0$ (Eq. (S505)), Eq. (S511) implies:

$$Z'_P(N_P^{-1}(0) < C_i < H_P^{-1}(0)) > 0 \quad \text{(S512)}$$

By using $N_P^{-1}(0) = Z_P^{-1}(0)$ (Eq. (S509)) and $H_P^{-1}(0) = F_P^{-1}(*)$ (Eq. (S508)), it follows from Eq. (S512):

$$Z'_P(Z_P^{-1}(0) < C_i < F_P^{-1}(*)) > 0 \quad \text{(S513)}$$

SI 7 Two criteria $C_i > 0$ and $g_s > 0$

The requirement of two criteria ($C_i > 0$ and $g_s > 0$) for ensuring the existence and uniqueness of a solution is far from trivial. This is because it is possible that either condition alone might suffice, or that additional conditions could be necessary. Let us first consider the case where only $C_i > 0$ is assumed. In this case, as shown in Figure 4 (a), there are two intersections satisfying $Z(C_i) = Z_c$, which means the uniqueness of the solution cannot be guaranteed. By introducing the condition $g_s > 0$, the range of solutions is restricted, and it can be demonstrated that there is always a single solution within this range. Similarly, if we were to assume only $g_s > 0$, we would consider the region where $F(C_i) > 0$. For example, as shown in Figure 2 (d), there exists a solution satisfying $Z(C_i) = F_{R4}(C_i) = Z_c$ in the region where $C_i < 0$. Therefore, it is only with the combination of these two criteria ($C_i > 0$ and $g_s > 0$) that the existence and uniqueness of the solution can be proven. Furthermore, this proof also demonstrates that no additional conditions are necessary beyond these two. It should also be noted that, by these two criteria, the other non-negative internal variables e_s and C_s are also guaranteed to be positive as well ($e_s = \frac{g_s e_i + g_b e_a}{g_s + g_b}$ and $C_s = \frac{\lambda_b g_s C_i + \lambda_s g_b C_a}{\lambda_b g_s + \lambda_s g_b}$).

Under typical atmospheric conditions, more precisely when the ambient CO_2 concentration (C_a) is greater than the CO_2 compensation point of net photosynthesis $A_{nR}^{-1}(0)$, that is, $C_a > A_{nR}^{-1}(0)$, the unique solution derived in this study always satisfies $C_i > A_{nR}^{-1}(0)$, as shown in Table 4. Because $A_{nR}^{-1}(0) > \Gamma^*$, the solution necessarily satisfies $C_i \leq \Gamma^*$. Therefore, even if the stricter physiological constraint $C_i \leq \Gamma^*$ is imposed, the existence and uniqueness of the solution can still be rigorously proven under the condition $C_a > A_{nR}^{-1}(0)$. However, when C_a becomes extremely small–for example, $C_a < \Gamma*$, during experimental measurements such as A–C_i response curves, where C_a is artificially reduced–solutions with $C_i < \Gamma^*$may occur. For this reason, the criterion adopted in this study is $C_i > 0$, which guarantees the existence and uniqueness of solutions for arbitrary $C_a > 0$.

SI 8 Non-existence of Solutions for $A_{np}(C_i)$

According to [von Caemmerer, 2000, Busch et al., 2018], the net photosynthesis rate limited by TPU, $A_{np}(C_i)$, is expressed as:

$$A_{np}(C_i) = \frac{a\left\{C_i - \Gamma^*\right\}}{C_i - p\Gamma^*} - R_L, \quad (p\Gamma^* < C_i) \tag{S514}$$

where $a = 3T_p$ and $1 \leq p \leq 4$. Note that $A_{np}(C_i)$ is defined only for $C_i > p\Gamma^*$. Using this formulation, $F_p(C_i)$, the function $F(C_i)$ for the TPU-limited case, is given by:

$$F_p(C_i) = -\frac{\lambda_s A_{np}(\pm\infty)\left\{C_i - A_{np}^{-1}(0)\right\}}{H_p(C_i)}, \tag{S515}$$

where $H_p(C_i)$ is defined as:

$$H_p(C_i) = C_i^2 - \left\{C_a + A_{np}^{-1}(*) - \lambda_b \frac{A_{np}(\pm\infty)}{g_b}\right\} C_i + C_a A_{np}^{-1}(*) - \lambda_b \frac{A_{np}(\pm\infty)}{g_b} A_{np}^{-1}(0), \tag{S516}$$

and $A_{np}(\pm\infty)$, $A_{np}^{-1}(0)$, and $A_{np}^{-1}(*)$ are given by:

$$A_{np}(\pm\infty) = a - R_L, \tag{S517}$$

$$A_{np}^{-1}(0) = \frac{\left\{a - pR_L\right\}\Gamma^*}{a - R_L}, \tag{S518}$$

$$A_{np}^{-1}(*) = p\Gamma^*. \tag{S519}$$

The determinant of $H_p(C_i)$, D_{H_p}, can be expressed as a function of C_a as follows:

$$D_{H_p}(C_a) = \left\{C_a - \left\{p\Gamma^* + \frac{\lambda_b\left\{a - R_L\right\}}{g_b}\right\}\right\}^2 + \frac{4\lambda_b\Gamma^*}{g_b} a\left\{1 - p\right\}. \tag{S520}$$

It is straightforward to identify environmental conditions, such as certain values of a, R_L, C_a, Γ^*, p, and g_b, that lead to $A_{np}(\pm\infty) = a - R_L > 0$ and $D_{H_p}(C_a) < 0$. In such cases, since $D_{H_p}(C_a) < 0$ and the leading coefficient of H_p is positive, we have $H_p(C_i) > 0$ for all C_i. Combining this with Eq. (S515), it follows that $F_p(C_i) < 0$ for $C_i > A_{np}^{-1}(0)$, which implies $g_s < 0$ in that range. Therefore, under these environmental conditions, no biologically or physically meaningful solutions exist at $C_i > A_{np}^{-1}(0)$. Note that if $A_{np}(\pm\infty) = a - R_L > 0$, then $A_{np}^{-1}(0) < p\Gamma^*$, while $A_{np}(C_i)$ is defined only for $C_i > p\Gamma^*$.

In the case where no solutions exist under TPU-limited conditions, any numerical attempt to obtain such a solution will necessarily fail. Therefore, identifying the environmental conditions

under which this situation occurs is of great significance. On the other hand, when no solutions exist under TPU-limited conditions, it is likely that the smaller of the solutions under the Rubisco-limited or RuBP-limited conditions is realized in nature; however, this point requires further investigation.
